# Identification of Differentially-Expressed Genes in Response to *Mycosphaerella fijiensis* in the Resistant *Musa* Accession ‘Calcutta-4’ Using Suppression Subtractive Hybridization

**DOI:** 10.1371/journal.pone.0160083

**Published:** 2016-08-03

**Authors:** Eduardo Sánchez Timm, Lisette Hidalgo Pardo, Ricardo Pacheco Coello, Tatiana Chávez Navarrete, Oscar Navarrete Villegas, Efrén Santos Ordóñez

**Affiliations:** 1 Escuela Superior Politécnica del Litoral, ESPOL, Centro de Investigaciones Biotecnológicas del Ecuador, Campus Gustavo Galindo Km 30.5 Vía Perimetral, P.O. Box 09-01-5863, Guayaquil, Ecuador; 2 Escuela Superior Politécnica del Litoral, ESPOL, Facultad de Ciencias de la Vida, Campus Gustavo Galindo Km 30.5 Vía Perimetral, P.O. Box 09-01-5863, Guayaquil, Ecuador; College of Agricultural Sciences, UNITED STATES

## Abstract

Bananas and plantains are considered an important crop around the world. Banana production is affected by several constraints, of which Black Sigatoka Disease, caused by the fungus *Mycosphaerella fijiensis*, is considered one of the most important diseases in banana plantations. The banana accession ‘Calcutta-4’ has a natural resistance to Black Sigatoka; however, the fruit is not valuable for commercialization. Gene identification and expression studies in ‘Calcutta-4’ might reveal possible gene candidates for resistant to the disease and elucidate mechanisms for resistance. A subtracted cDNA library was generated from leaves after 6, 9 and 12 days inoculated with *M*. *fijiensis* conidia on greenhouse banana plants of the accession ‘Calcutta-4’. Bioinformatic analysis revealed 99 good quality sequences. Blast2go analysis revealed that 31% of the sequences could not be categorized and, according to the Biological Process Category, 32 and 28 ESTs are related to general metabolic and cellular processes, respectively; while 10 ESTs response to stimulus. Seven sequences were redundant and one was similar to genes that may be involved in pathogen resistance including the putative disease resistance protein RGA1. Genes encoding zinc finger domains were identified and may play an important role in pathogen resistance by inducing the expression of downstream genes. Expression analysis of four selected genes was performed using RT-qPCR during the early stage of the disease development at 6, 9, 12 and 15 days post inoculation showing a peak of up regulation at 9 or 12 days post inoculation. Three of the four genes showed an up-regulation of expression in ‘Calcutta-4’ when compared to ‘Williams’ after inoculation with *M*. *fijiensis*, suggesting a fine regulation of specific gene candidates that may lead to a resistance response. The genes identified in early responses in a plant-pathogen interaction may be relevant for the resistance response of ‘Calcutta-4’ to Black Sigatoka. Genes with different functions may play a role in plant response to the disease. The present study suggests a fine up regulation of these genes that might be needed to perform an incompatible interaction. Further gene functional studies need to be performed to validate their use as candidate resistance genes in susceptible banana cultivars.

## Introduction

From 2009 until 2011 more than 18000 tons of bananas were marketed. Most of the exporter banana production is generated in development countries such Ecuador, Colombia, Costa Rica and Guatemala, where all the production chain provides incomes to the local economy [1]. The European Union and the United States of America represent the biggest and most significant importers in the world, still the demand of the fruit will increase in China and other emerging countries until 2019 [2]. This crop represents an important market worldwide with a promising raise of production and trade. However, Black Sigatoka Disease (BSD) is a serious constraint in the banana and plantain industry since main commercial banana and plantain cultivars are susceptible to the disease. In Ecuador, important banana and plantain cultivars are threatened by BSD, especially in smallholders who could not control the disease chemically or in an integrated management. These cultivars include ‘Williams’ (genotype AAA), ‘Gran Enano’ (AAA), ‘Barraganete’ (AAB), and ‘Dominico’ (AAB).

The BSD is caused by the fungal pathogen *Mycosphaerella fijiensis*. The fungus penetrates the stomata of leaves causing necrotic damage, decreasing the photosynthetic capacity and, therefore, reducing the amount and value of the product [3]. Conventional control of the disease implies several fungicide applications, which is a critical problem for the environment, the health of persons in the surroundings of the plantations, and the profitability of the producers [4]. Furthermore, chemical control may develop fungicide resistance of the pathogen [5].

The molecular host-pathogen interaction between banana and *M*. *fijiensis* is not well understood yet. On the other hand, some accessions of *Musa* spp. are highly resistant to the BSD, as the mortality of the host cells occurs fast after infection avoiding and preventing the spread of the pathogen into the rest of the plant [6]. Therefore, resistance of *M*. *fijiensis* in *Musa* is obtained after stomata penetration by the production of plant compounds [6, 7, 8]. Consequently, genes from resistant accessions could be used for genetic engineering of susceptible bananas and plantain cultivars. For instance, the wild diploid accession ‘Calcutta-4’ (*Musa acuminata* ssp. *burmaniccoides*) have been used in breeding programs offering resistance to fungal pathogens including *M*. *fijiensis* [9]. The study of the interaction at a deeper level is necessary to understand the genes involved in this process.

Biotic stress gene expression analysis has been limited in banana. Studies have been focused in susceptible, tolerant or resistant banana varieties to *Fusarium oxysporum* [10, 11, 12, 13], *M*. *musicola* [14], and susceptible bananas for *M*. *fijiensis* [15]. Recently, identification and gene expression analysis have been performed in the resistant accession ‘Calcutta-4’ under *M*. *fijiensis* infection, where a full length cDNA library was performed on *in vitro* leaf disc assays collected from greenhouse plants [16]. Although, gene identification on such full-length cDNA library suggest gene expression under biotic stress, similar gene expression may also be encountered in mock inoculation, and therefore, further confirmation of genes identified should be performed for instance with RT-qPCR, for the hundreds of genes generated in the library. The suppression subtractive hybridization (SSH) allows enrichment of gene expressed under specific conditions, narrowing the number of genes expressed for further analysis. Therefore, SSH technique allows the generation of cDNA libraries through the amplification of fragments or tester that are differentially expressed from a control or driver [17]. One of the main applications of this technique is the identification of changes in gene expression during infections process like the attack of pathogens. In addition, SSH is useful to contrasts between dissimilar tissues [18].

This work presents the generation and sequencing of a cDNA subtracted library in an incompatible plant-pathogen interaction. Enriched expressed sequence tags (ESTs) were obtained from‘Calcutta-4’ leaf samples which were inoculated with conidia of *M*. *fijiensis* after 6, 9 and 12 days post inoculation (DPI) on a greenhouse assay on plants. Up-regulation of selected sequences was confirmed by RT-qPCR from samples collected in an independent bioassay on greenhouse plants. Therefore, a set of sequences was obtained for the discovery of candidate sequences suitable for genetic engineering in banana and to elucidate plant-pathogen interaction in a resistant banana accession like ‘Calcutta-4’.

## Materials and Methods

### Fungal and plant growth conditions

Plants of the accession‘Calcutta-4’ (AA, ITC.0249) and ‘Williams’ (AAA) were obtained from the International Network for the Improvement of Banana and Plantain Transit Center and the Tissue Culture department of the Biotechnology Research Center of Ecuador, respectively. ‘Calcutta-4’ and ‘Williams’ were micropropagated, and the *in vitro* plants were then grown at least for 6 months prior the bioassay in soil in bags inside of a greenhouse where the photoperiod was 12 hours of natural light. The estimated temperature was 28°C during the day, and the relative humidity range was between 80–90% during the bioassay, which was obtained by maintaining the plants in a transparent plastic aluminum-framed box. Fungal material of the species *Mycosphaerella fijiensis* local strains RSSB46, were obtained as conidia [19].

### Plant inoculation

Plant inoculation was performed according to Jiménez *et al*. [19] in two different and independent bioassays. The first bioassay (FB) was performed for the development of the SHH–cDNA library and the second bioassay (SB) was performed for gene expression analysis with RT-qPCR (see below). During both assays the leaves were sprayed with a 1% gelatin suspension containing 2x10^4^conidia/ml of *M*. *fijiensis*, two biological leaf samples were considered for the posterior analysis. Mock inoculation was performed for the “Control” plants using gelatin suspension lacking conidia.

### Sample collection

The third unfolded leaf of each plant was collected at 6, 9 and 12 DPI for the FB. For the SB, the third unfolded leaf of each plant was collected at 6, 9, 12 and 15 DPI. For both bioassays the selected tissue was submerged in liquid nitrogen and maintained at -80°C until RNA extraction. An additional evaluation of disease severity was performed in the FB during the collection days and 15, 30, 45 and 60 DPI according to Alvarado *et al*. [20] for confirmation of Black Sigatoka disease development in the susceptible banana cultivar ‘Williams’ to ensure success of the bioassay. Finally the evaluation of the SB disease severity was performed weekly after 15 DPI of *M*. *fijiensis* up to the 7^th^ evaluation.

### Total RNA extraction and SSH-cDNA library

Total RNA was isolated with Spectrum^TM^ Plant Total RNA kit (Sigma-Aldrich, United States). Approximated 100 mg of initial material was used for each reaction. The RNA was quantified using the Synergy HT Multi-Mode Microplate Reader (BIOTEK, United States). The extracted RNA was treated with RQ1 RNase-Free DNase (Promega, United States). Finally, to verify the absence of genomic DNA, a PCR was performed using the DNase-Treated RNA as template and primers amplifying the Actin gene (GeneBank No. AF285176; ActinF3, CCCAAGGCAAACCGAGAGAAG; ActinR2, GTGGCTCACACCATCACCAG, [21]. The reaction mix used as final concentrations was 1X of GoTaq®Green Master Mix (Promega, United States) and 200 nM of primers. The PCR program was as follows: 95°C 2min, then 45 cycles of 95°C 30seg, 60°C 30seg and 72°C 45seg, and a final cycle of 72°C 5min.

From 50–100 μg of total RNA, mRNA isolation was performed with the Oligotex mRNA Mini kit (Qiagen, United States). The resulted mRNA was concentrated and used in the PCR-Select^TM^ cDNA Subtraction kit (Clontech, United States) according to the manufacturer. The cDNA of inoculated ‘Calcutta-4’ leaves harvested during 6, 9 and 12 DPI were used as “tester” and the cDNA from non-inoculated ‘Calcutta-4’ leaves (mock inoculation) were used as “driver”. The resulting subtracted cDNA was cloned into the pGEM vector using the pGEM®-T and pGEM®-T Easy Vector Systems (Promega, United States) and transformed into chemical competent cells of TOP10® *Escherichia coli* (Invitrogen^TM^, United States). In a 96-well plate, the bacterial clones were grown in lysogeny broth medium with Ampicillin (50 μg/μl) and stored at -80°C in glycerol stocks. The presence and size of inserts of all clones were checked by colony PCR.

### EST sequencing and sequence analysis

An alkaline plasmid extraction was performed to all the selected *E*. *coli* colonies. The extracted plasmids were diluted as required by Macrogen Sequencing Services (Macrogen Inc, United States). Raw sequences were clear up, from vector and cloning adaptors, with the program CLC Genomics Workbench (CLC bio, Denmark). The sequences are available in Supporting Information (S1 File). The primer design from the ESTs was done with the program Primer3 version 2.2.3. Nucleotide homology was based in blastn in the banana genome [22]. Blast2Go was used to characterize the ESTs and to select the most KEGG pathways affected. Selected ESTs for RT-qPCR were analyzed by identification of GO terms to define KEGG orthologs. Blastx analysis was performed on selected sequences on the KEGG database [23]. Description of coding protein was queried from the gene name identified in the KEGG database or the translated protein sequence in the Interpro database [24]. These sequences were queried in the banana genome [22] and further analyzed for metabolic pathway [25].

### Relative quantification of ESTs through RTqPCR

Total RNA was isolated from the SB samples using the PowerPlant® RNA isolation kit (Mo Bio Laboratories, Inc, United States). Up to 50 mg of initial material was used for each sample. The cDNA synthesis was achieved with the SuperScript® III First-Strand Synthesis System (Invitrogen^TM^, United States) using 1 μg of total RNA. Reactions of RT-qPCR were performed in Micro Amp Optical 96-well reaction plate (Applied Biosystems, United States) using a Mastercyclerep Realplex^4^ (Eppendorf, Germany) and SYBR Green to monitor the template amplification. Each reaction contained 1X SYBR GreenER^TM^qPCR Super Mix Universal (Invitrogen^TM^, United States) and 150 nM of each gene-specific primers in a final volume of 10 μl. PCR conditions were 50°C 2 min for uracil DNA glycosylase incubation, 95°C 15 min, then 40 cycles of 95°C 15 sec., 60°C 20 sec. and 72°C 20 sec., finally one cycle of 72°C 2 min. Non-inoculated samples were used as “calibrator” and the inoculated samples were used as “experiment”. RT-qPCR analysis was performed using three independent biological samples collected from the SB. All the Ct values were transformed into relative expression by applying the Pfaffl method (2001). The relative expression of three biological replicates were analyzed with geNorm v 3.5 [26] and Normfinder [27] tools, as described in each manual, for stability determination of the reference genes. The normalization factor used in the calculation was based on a geometric mean of the quantities of the most stable reference genes which include the elongation factor-1a (primer forward: CGGAGCGTGAAAGAGGAAT, primer reverse R: ACCAGCTTCAAAACCACCAG), the β-tubulin (primer forward: TGTTGCATCCTGGTACTGCT, primer reverse: GGCTTTCTTGCACTGGTACAC), the ribosomal protein L2 (primer forward: AGGGTTCATAGCCACACCAC, primer reverse: CCGAACTGAGAAGCCCCTAC), and the actin (primer forward: GAGAAGATACAGTGTCTGGA, primer reverse: ATTACCATCGAAATATTAAAAG) according to Podevin *et al*. [28].

Four different genes were selected based on different characteristics including novelty, carbon and starch metabolisms, and pathogen resistance related. The primers used for the four different genes (S1 Table) were: 1E3: SSH1-E3F (TGTTGATCCTGACCCAGTGCG), SSH1-E3R (TGGACTGAACCCTCCCAAAG); 1C2: SSH1-C2F (TTGGAGATCTCTGATGGAGCTG), SSH1-C2R (GGAGCAGCCCAAATTACACG); 1D8: SSH1-D8F (AAGAGGCACGGAAGAAGACG), SSH1-D8R (AGCAGATCGTTGCCGAGAG); SSH1-G5F (GTCCGAATACGCTGGTGAATG), SSH1-G5R (CTTAAACAACCCGCCTACCG).

Real-time qPCR efficiency was calculated for each gene by using the slope of a linear regression model of the dilution series [E = 10(-1/slope)] [28]. A correlation coefficient R^2^ above 0.98 was observed for all PCR reactions. To verify the specificity of the amplicon for each primer pair, a melting curve was produced from 55 to 95°C at the end of each RT-qPCR program.

## Results

### Greenhouse pathogen inoculation

**Fig 1:**
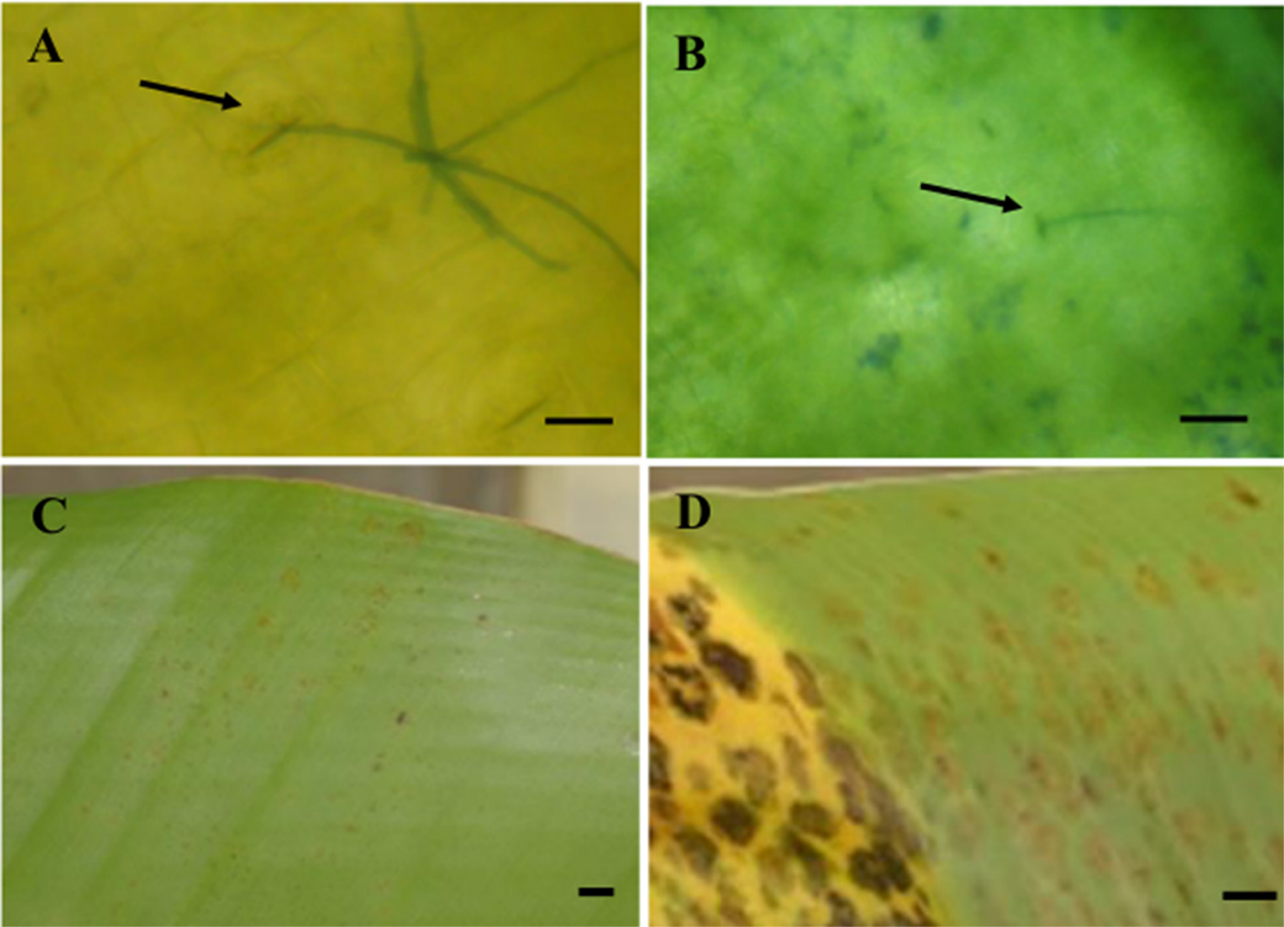
Inoculation of *M*. *fijiensis* in ‘Williams’ and ‘Calcutta-4’. The first bioassay was performed on six months old banana plants grown in soil in bags in the greenhouse. *M*. *fijiensis* hyphae (arrow) penetrating banana leaf through stomata after 6 days post inoculation in the resistant accession ‘Calcutta-4’, bar indicates 15 μM (A) and the susceptible banana cultivar ‘Williams’, bar indicates 45 μM (B). ‘Calcutta-4’, 28 days post inoculation (DPI), bar indicates 5 mm (C). ‘Williams’, 28 days post inoculation (DPI), bar indicates 5 mm (D).

Greenhouse inoculation assay for the SSH library generation was performed in the Black Sigatoka-natural resistant accession ‘Calcutta-4’. To ensure success of the assay, the susceptible cultivar ‘Williams’ (dessert banana, AAA) was used as control for the assay. Furthermore, stomatal penetration of *M*. *fijiensis* hyphae was detected on the susceptible and resistant variety after 2, 4 and 6 days of *M*. *fijiensis* inoculation under microscope observation (Fig 1A and 1B). Symptom development was obtained in the susceptible cultivar. At 28 DPI, cleared symptoms of grade 4 was detected in the susceptible cultivar ‘Williams’, while in ‘Calcutta-4’ symptoms were increased up to grade 1 or 2 (Fig 1C and 1D).

**Fig 2:**
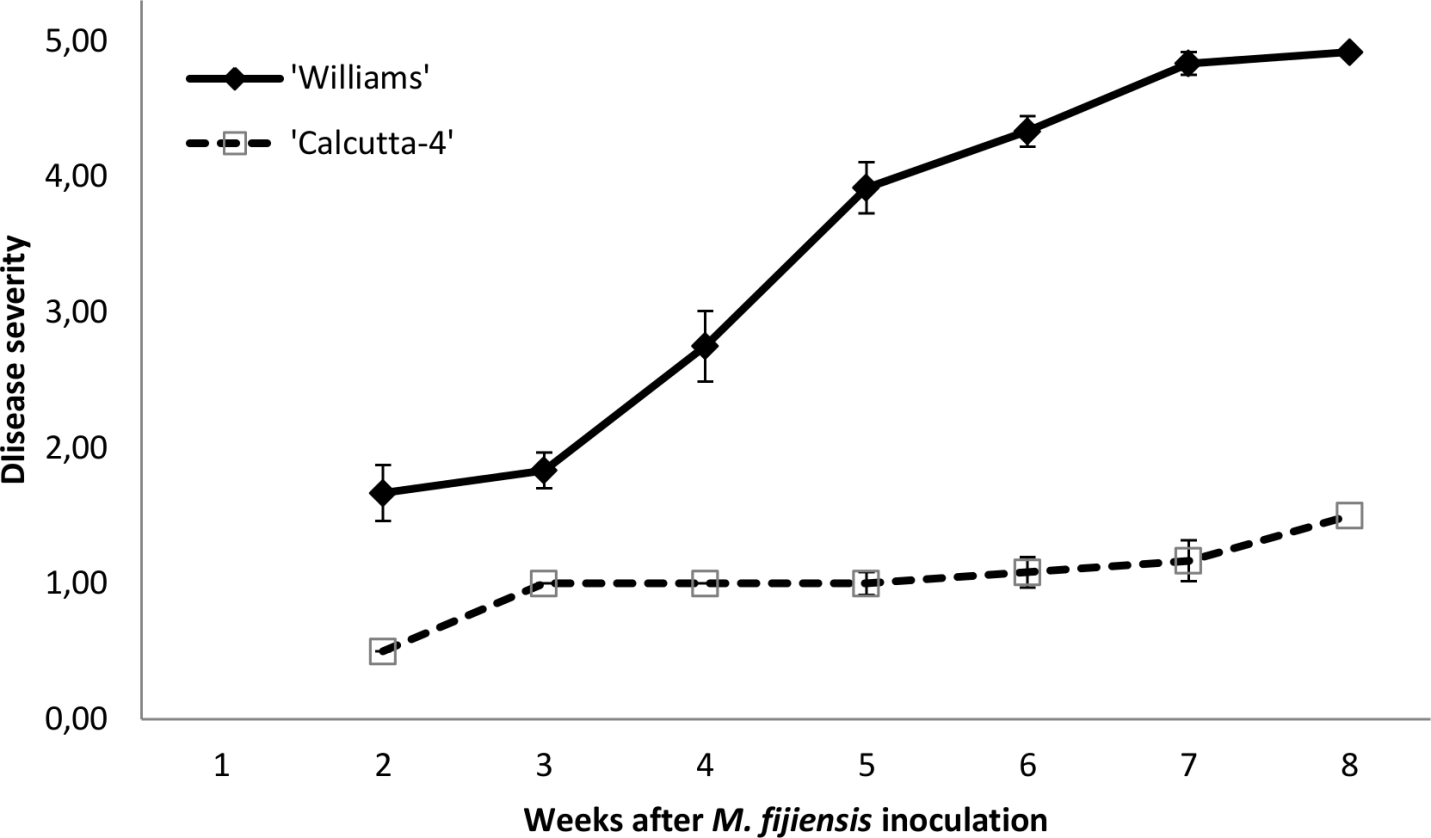
Disease development of Black Sigatoka in the susceptible cultivar ‘Williams’ and the resistant accession ‘Calcutta-4’. Disease development recorded after 15 days post inoculation (DPI) and assessed weekly until 57 DPI. Bars indicate standard error of 12 plants assessed.

For the bioassay of gene expression analysis with RT-qPCR, symptom development was normally obtained up to grade 5 in the susceptible cultivar ‘Williams’ after 7 weeks, while the accession ‘Calcutta-4’ was obtained up to grade 1 or 2 of severity, corresponding to an hypersensitive response (Fig 2).

### Analysis of sequences generated in the subtracted cDNA library

**Fig 3:**
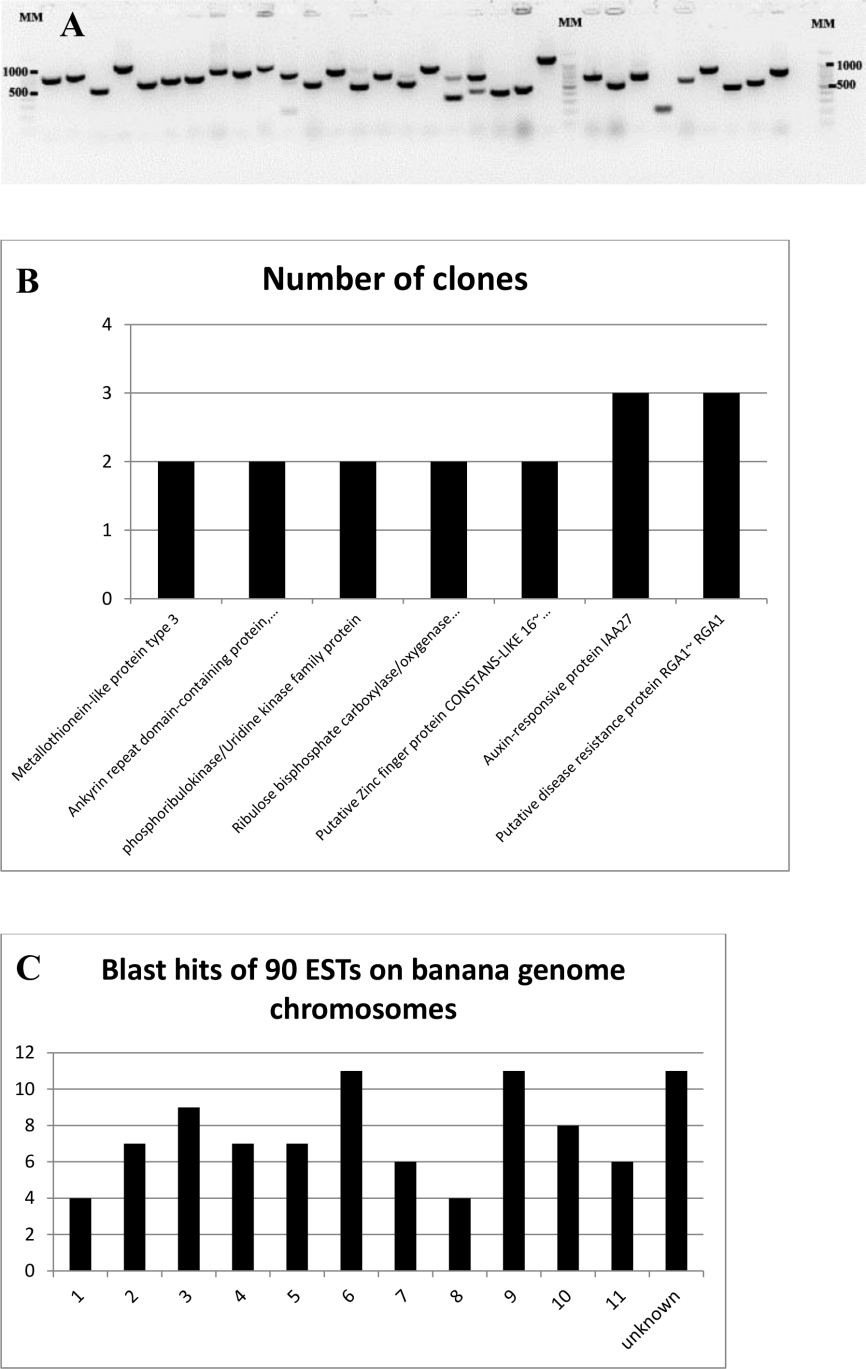
Suppression subtractive library generation. Colony PCR of antibiotic resistant *E*. *coli* using primers M13F and M13R. MM: molecular marker of 1000 bp Ladder was used (A). ESTs redundancy in SSH library, number of clones containing similar sequences (B). Blast hits of ETS on the banana genome chromosomes, location of 90 ESTs on the banana genome (C).

A subtracted cDNA library was generated by pooling total ARN from leaf number three of different ‘Calcutta-4’ greenhouse plants inoculated with *M*. *fijiensis* conidia after 6, 9 and 12 days of inoculation. Therefore, the subtracted library should contain genes up-regulated after penetration of pathogen through stomata, which is important in the early stages of disease development in an incompatible *M*. *fijiensis*-banana interaction. Subtractive sequences expressed during *M*. *fijiensis* infection were cloned in pGEM vector and multiplied in *E*. *coli*. The plasmids of a total of 135 random colonies were selected after colony-PCR (Fig 3A). Ninety-nine showed to be good quality sequences. Redundancy was obtained in seven sequences (Fig 3B) obtaining 90 unigenes. Bioinformatic analysis revealed that seven sequences were similar to unannotated regions of the banana genome ‘DH Pahang’. Out of the seven sequences, four correspond to non-coding RNA including chloroplastic 23S ribosomal RNA and mitochondrial 26S ribosomal RNA (S1 Table). Bioinformatic analysis revealed that most of the ESTs are annotated in the banana genome (S1 Table). Sequences were submitted at the GenBank and expecting for publication (sequences available in Supporting Information, S1 File). Similarities of one sequence reveal a gene that may be involved in pathogen resistant like the putative disease resistance protein RGA1 (RGA1) (GSMUA_Achr3G22310_001~ Putative disease resistance RPP13-like protein 1~ RGA1~ missing completeness). Three ESTs showed similarities to this gene in the banana genome (Fig 3B). The location of the ESTs on the banana genome chromosomes is indicated, with the highest numbers in chromosome (chr) 6 (11 sequences), chr 9 (11 sequences), unknown chromosome (9 sequences), chr3 (9 sequences) and chr10 (8 sequences) (Fig 3C).

### Functional classification of the expressed genes

**Fig 4:**
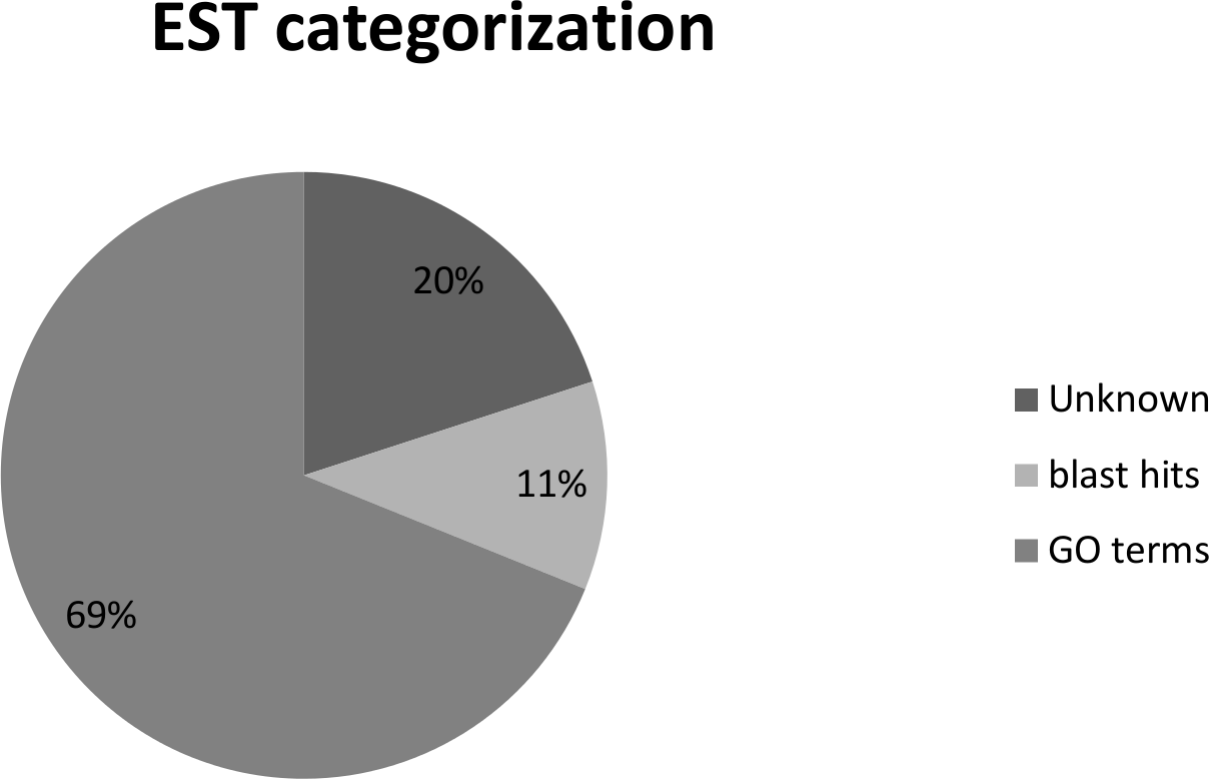
Categorization of ESTs by blast2Go. General classification of sequences according to GO annotation.

**Fig 5:**
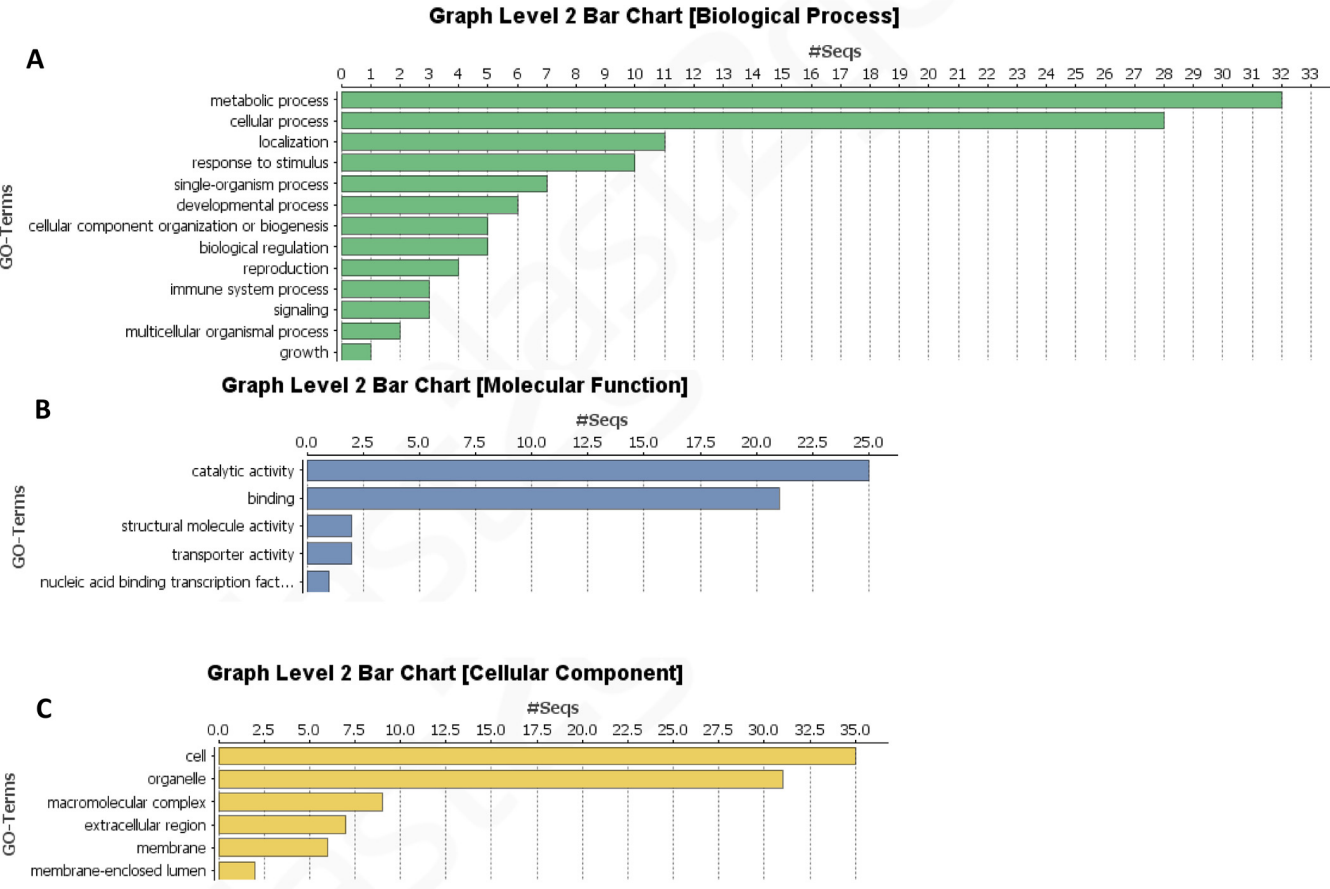
Categorization of ESTs by blast2Go level 2. Classification of Biological Process (A), Molecular Function (B) and Cellular Component (C) of ESTs. Several sequences could be categorized in different GO IDs.

Blast2go analysis revealed that 31% of the sequences could not be categorized and, according to the Biological Process Category, 32 and 28 ESTs are related to general metabolic and cellular processes, respectively; while 10 ESTs response to stimulus (Fig 4 and Fig 5A). Most of the sequences were categorized in molecular function as catalytic activity and binding (Fig 5B); while in cellular component the majority were cell and organelle (Fig 5C). After the Blast2go analysis, six KEGG pathways presented a higher rate of encountered genes (Table 1).

**Table 1:** The six KEGG pathways with most genes encountered in the SSH library using Blast2go analysis.

KEGG pathways	No. Sequences	No. Enzymes	Enzymes
Glycolisis/Gluconeogenesis	4	4	ec:5.1.3.3–1-epimerase, ec:1.1.1.1 –dehydrogenase, ec:1.2.1.12—dehydrogenase (phosphorylating), ec:4.1.2.13- aldolase
Drug metabolism -cytochrome P450	3	2	ec:2.5.1.18 –transferase, ec:1.1.1.1—dehydrogenase
Metabolism of xenobiotics by cytochrome P450	3	2	ec:2.5.1.18 –transferase, ec:1.1.1.1—dehydrogenase
Thiamine metabolism	3	1	ec:3.6.1.15
Purine metabolism	3	2	ec:3.6.1.3 –adenylpyrophosphatase, ec:3,.6.1.15
Carbon fixation in photosynthetic organisms	3	3	ec:2.7.1.19 –phosphopentokinase, ec:1.2.1.12—dehydrogenase (phosphorylating), ec:4.1.2.13—aldolase

### Quantitative expression analysis of selected ESTs during the infection process

**Fig 6:**
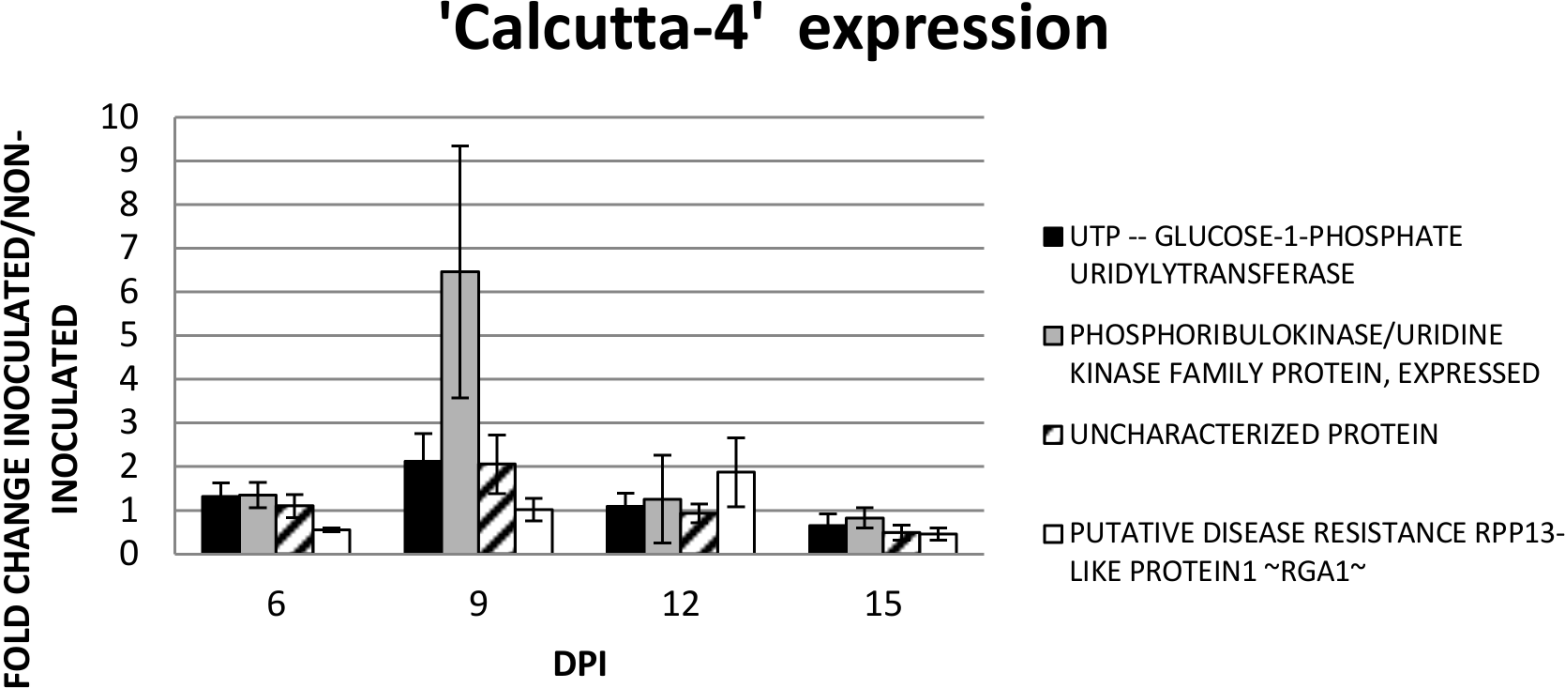
Gene expression analysis in a second greenhouse bioassay. Relative quantification was performed using RT-qPCR in three biological replications independently. Fold changes is the ratio of gene expression of the inoculated ‘Calcutta-4’ compared to the mock inoculations.

Four sequences were selected for RT-qPCR analysis based on the hypothetical biological significance in plant defense response of ‘Calcutta-4’ to *M*. *fijiensis*, which belong to different kind of gene functions. Individual analysis of gene expression was performed at early stages of disease development including 6, 9, 12 and 15 days post inoculation, compared to mock inoculation samples (Tables 2 and 3; Fig 6).

**Table 2:** Similarity search using blastn of selected ESTs for RT-qPCR analysis at the banana and plantain genome database.

Clone	BANANA GENOME ('DH PAHANG')				PLANTAIN GENOME ('PKW')			
	E-value	% Identity	*Locus*	Description/function	E-value	% Identity	Locus	Description/function
1E3	e-156	99%	GSMUA_Achr5P03850_001	Uncharacterized protein At1g03900	e-155	98%	ITC1587_Bchr5_P11991	serine threonine-protein kinase cbk1-like
1C2	e-135	98%	GSMUA_Achr9P23930_001	UTP—glucose-1-phosphate uridylyltransferase	e-108	95%	ITC1587_Bchr9_P27698	udp-glucose pyrophosphorylase
1D8	e-130	98%	GSMUA_Achr5P03660_001	Phosphoribulokinase/Uridine kinase family protein, expressed	e-130	98%	ITC1587_Bchr5_P11976	phosphoribulokinase
1G5	2e-82	86%	GSMUA_Achr3G22310_001	Putative disease resistance protein RGA1	2e-54	85%	ITC1587_Bchr3_P07503	disease resistance protein at3g14460-like

**Table 3:** KEGG and banana genome metabolic pathways of selected ESTs for RT-qPCR analysis.

Clone	KEGG				METABOLIC PATHWAY BANANA GENOME		
	blastx	E-value	KEGG code	KEGG PATHWAY	GO Biological process	GO Molecular function	GO Cellular component
1E3	mus:103983864 uncharacterized protein At1g03900-like	2e-95			GO:0006897—endocytosis		GO:0016020—membrane
1C2	mus:103998808 UTP—glucose-1-phosphate uridylyltransferase	3e-08	K00963	mus00040 Pentose and glucuronate interconversionsmus00052 Galactose metabolismmus00500 Starch and sucrose metabolismmus00520 Amino sugar and nucleotide sugar metabolismmus01100 Metabolic pathways mus01110 Biosynthesis of secondary metabolites	GO:0008152—metabolic process	GO:0016779 –nucleotidyl transferase activity	
1D8	mus:103983846 phosphoribulokinase, chloroplastic-like	3e-61	K00855	mus00710 Carbon fixation in photosynthetic organisms mus01100 Metabolic pathways mus01200 Carbon metabolism	GO:0005975—carbohydrate metabolic processGO:0008152—metabolic process	GO:0005524—ATP bindingGO:0008974—phosphoribulokinase activityGO:0016301—kinase activity	
1G5	mus:103975156 putative disease resistance RPP13-like protein 1	9e-80			GO:0006915—apoptosis	GO:0005524—ATP binding	

The peak of gene expression induction was detected at 9 DPI in three of the four genes analyzed, except for the *RGA1*, showing the highest up regulation after 12 DPI (Fig 6). Induction of gene expression up to six fold in average was detected in the *phosphoribulokinase/Uridine kinase family protein* gene (*PUK*) at 9 DPI. The *RGA1* gene showed an up-regulated expression of 2-fold only at 12 DPI and a stable expression was detected at 9 DPI in the treated and mock inoculated plants. The *UTP—glucose-1-phosphate uridylyltransferase* gene (*UGP*) showed an up-regulation at 9 DPI in banana, while the uncharacterized protein (*UCP*) only showed up regulation at 12 DPI. The *UCP* showed a more stable expression for 6, 9 and 12 DPI. Down regulation of the four genes was observed at 15 DPI.

**Fig 7:**
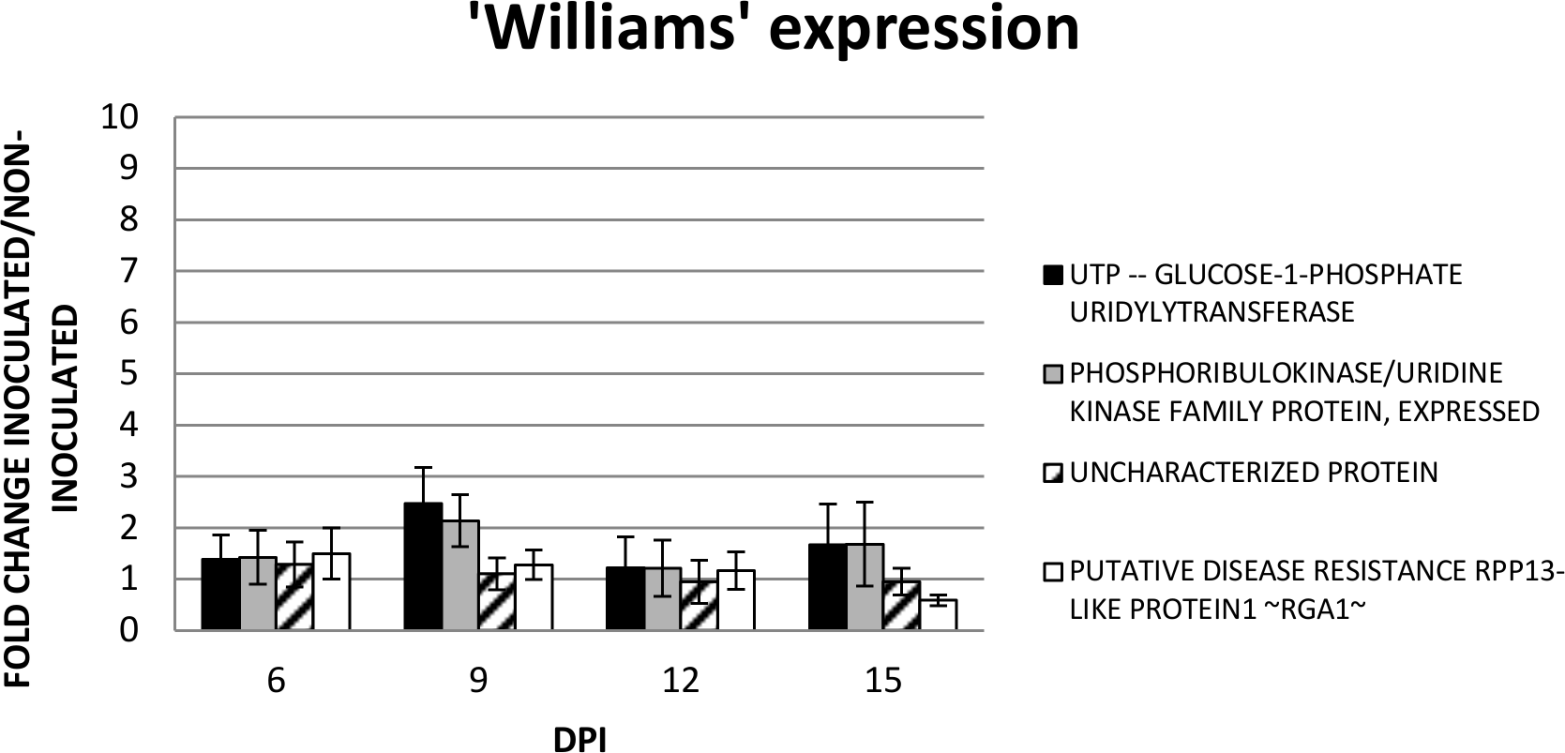
Gene expression analysis in a second greenhouse bioassay. Relative quantification was performed using RT-qPCR in three biological replications independently. Fold changes is the ratio of gene expression of the inoculated ‘Williams’ compared to the mock inoculations.

Expression analysis was also followed in the susceptible banana cultivar ‘Williams’ between inoculated and mock inoculated samples (Fig 7). The highest induction of expression were observed in the genes *UGP* and *PUK* at 9 DPI of up to 2.5 fold induction, followed for an up-regulation of 1.5 fold of the same genes at 15 DPI. The *UCP* showed a more stable expression during the four time points analyzed, similar to the *RGA1* gene; although a down-regulation was observed at 15 DPI.

**Fig 8:**
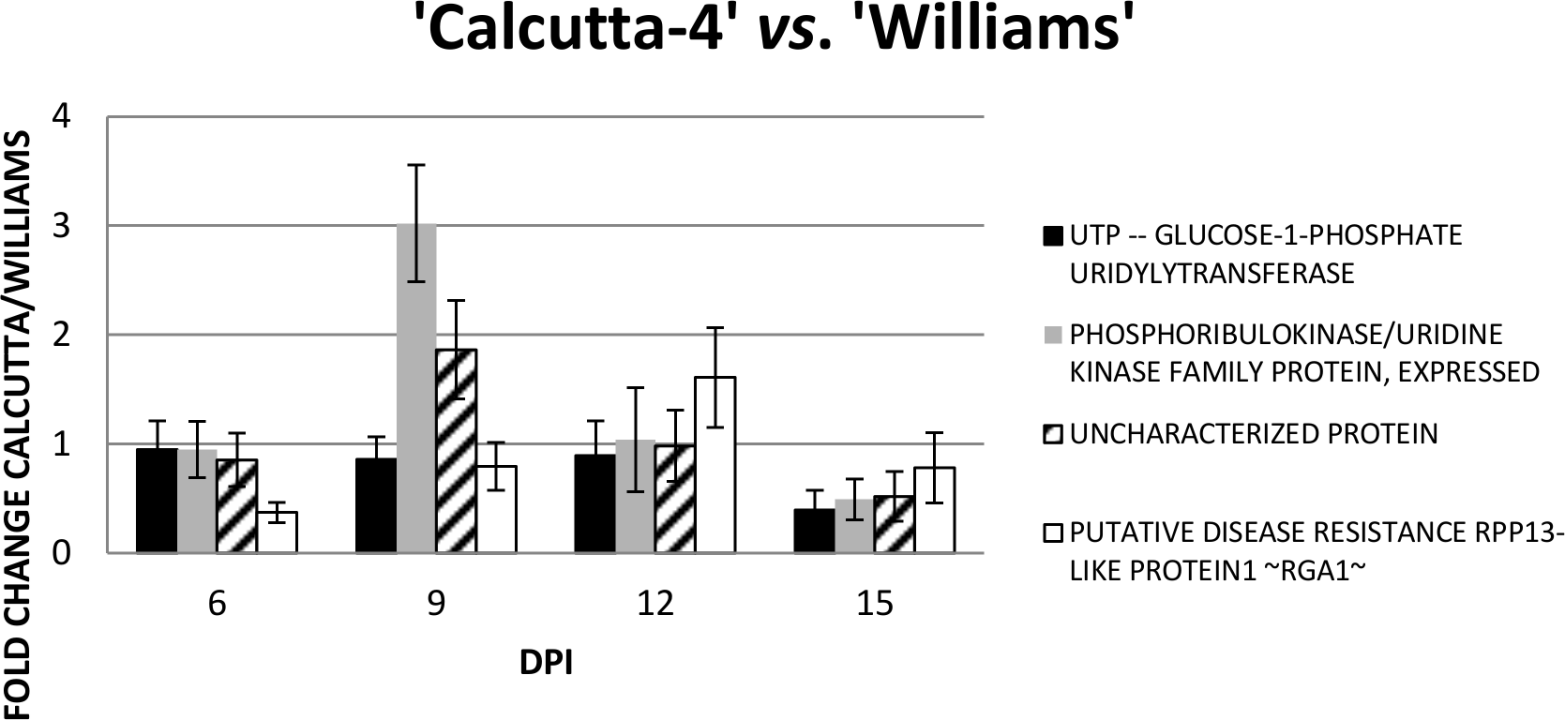
Gene expression analysis in a second greenhouse bioassay. Relative quantification was performed using RT-qPCR in three biological replications independently. Fold changes is the ratio of gene expression of the inoculated ‘Calcutta-4’ and ‘Williams’ compared to the gene expression of the inoculated ‘Williams’.

For the analysis of differential gene expression of the four genes between the resistant ‘Calcutta-4’ and the susceptible ‘Williams’, fold change was calculated (Fig 8). Major differences were observed in the *PUK* and the *UCP* showing an up regulation in ‘Calcutta-4’ of three and two fold induction at 9 DPI. The *RGA1* gene was more expressed in ‘Williams’ at 6 and 9 DPI; however, in ‘Calcutta-4’ the expression was more at 12 DPI. The four genes were more expressed in ‘Williams’ than in ‘Calcutta-4’ at 15 DPI.

## Discussion

### Bioassay

Black Sigatoka is the major disease in bananas and plantains cultivation areas. Wild *Musa* relatives including ‘Calcutta-4’ could be a major source of candidate resistance genes for BSD and for the elucidation at the molecular level of the resistance response. As a first step of a genetic improvement program for Ecuadorian banana and plantain cultivars, the identification of candidate resistance genes was performed using a SSH strategy on ‘Calcutta-4’. Plant-pathogen interaction was studied on lived plants on bags with at least six months on greenhouse from vitroplantlets, showing expected disease development in the susceptible banana ‘Williams’ and a resistant response in ‘Calcutta-4’, diminishing the probability to obtain inconsistent results that might be more frequent in other bioassays like detached leafs, as discussed by Passos *et al*. [14].

Early biotrophic phase during infection of *M*. *fijiensis* in banana includes germ tube penetration through stomata from 3 to 6 DPI [29]. Although different bioassays conditions may imply different DPI for penetration of *M*. *fijiensis* hyphae in banana leaves, stomatal penetration determination was done in this study showing that between 2–6 days after conidia inoculation in greenhouse plants, stomatal penetration of the fungus occurred. Therefore, differential gene expression between *M*. *fijiensis* inoculated and mock inoculated ‘Calcutta-4’ plants after this period is expected.

### Gene annotation and analysis

The banana and plantain genome sequences makes the mapping and full-length characterization of the ESTs straight-forward; although, there were few sequences that showed similarities to non-coding regions of the banana genome. Not only genes involved in pathogen resistant were found, but differentially expressed genes showed different functions from general metabolic process, transcriptional regulations, and with unknown specific process, suggesting that different processes in the plant metabolism are affected upon infection of pathogen in an incompatible interaction. Different plant metabolic pathways are affected in pathogen resistant response including synthesis or degradation of carbohydrates, amino acids and lipids [30]. Gene expression induction up to 6-fold was detected in selected genes, suggesting a fine regulated gene expression in early process of disease development.

Different pathways are affected during the early response of *M*. *fijiensis* in ‘Calcutta-4’ (Table 1). The pathway of glycolysis/gluconeogenesis contained the most genes (four) of the SSH library involved. Different plant pathogen interactions affect the glycolysis/gluconeogenesis pathway including soybean-*Meloidogyne incognita* [31], Arabidopsis-*Pseudomonas syringae* [32], and wheat-*Puccinia triticina* [33]. This pathway contains enzymes important in other pathways like drug metabolism-cytochrome P450, metabolism of xenobiotics by cytochrome P450 (ec:1.1.1.1—dehydrogenase), and carbon fixation in photosynthetic organisms (ec:1.2.1.12 –dehydrogenase, phosphorylating; and ec:4.1.2.13 –aldolase), encountered in this study (Table 1). The first two pathways also share the ec:2.5.1.18 –transferase. This pathways may be important for processing the fungal compounds after infection. The P450 pathways are also affected in plant-pathogen interaction like banana-*Fusarium oxysporum* f. sp. *Cubense* tropical race 4 [12]. Cytochrome P450 could be classified in two functions: biosynthesis and detoxification. Boddu *et al*. [34] suggest that the synthesis of P450 genes might play a crucial role in protecting barley from toxic effects of trichocenes. Furthermore, P450 is involved in the synthesis of a variety of phenylpropanoid pathway compounds important for plant protection including flavonoids, phytoalexins, and lignin [34].

### Genes related to different stresses in plant

Metallothioneins are involved in reactive oxygen species (ROS) scavenging, which is expected in a hypersensitive response presented in ‘Calcutta-4’. Down-regulation of metallothionein is observed during oxidative burst in treated cells. Therefore, expression of metallothionein genes is important in defense mechanism pathways [35, 36]. In pine trees, the metallothionein expression was induced by 1 DPI in susceptible trees infected with the pine wood nematode, providing evidence for rapid induction of defense response genes such as those encoding pathogenesis related proteins in susceptible trees [37]. The hypersensitive response in the resistant accession ‘Calcutta-4’ occurs during *M*. *fijiensis* infection; therefore, the expression of metallothionein genes may play an important role in this interaction. Different studies in plant pathogen interaction in banana revealed the expression of Metallothioneins in interaction with *M*. *fijiensis* [38], *M*. *musicola* [14] and *Fusarium oxysporum* [39].

Proteins containing zinc finger domains display considerable versatility in binding modes of metals, DNA/RNA, proteins or lipid substrates, and could be involved in different cellular functions including transcription and translation. The cotton *CCCH-type tandem zinc finger* gene defined as *GhTZF1* was expressed in different vegetative and reproductive tissues [40]. Furthermore, this gene was up-regulated in different abiotic stresses including salt and polyethylene glycol in cotton. Enhanced oxidative stress tolerance was found after overexpression, suggesting that the *GhTZF1* functions as a modulator of ROS for regulation of drought tolerance and leaf senescence [40]. The banana *MusaSAP1* gene, which code for an A20/AN1 zinc finger domain, is up-regulated by drought, salt, cold, heat and oxidative stress as well as by treatment with abscisic acid [41]. Wounding and methyl jasmonate treatment also induced *MusaSAP1* suggesting involvement in biotic stress. The banana *MaCOL1* contained two B-box zinc finger motifs and a CCT domain. This CONSTANS-like *MaCOL1* banana gene is also involved in abiotic and biotic stress such as chilling and pathogen *Colletotrichum musae* infection [42].

The uncharacterized banana protein gene which showed only an up regulation after 12 DPI with *M*. *fijiensis* in ‘Calcutta-4’ (Fig 6) is similar to an adaptin ear-binding coat associated protein NECAP-1 according to a search in the Kyoto Encyclopaedia of Genes and Genomes (KEGG) database (Table 3) and in InterPro. This gene is poorly studied in plants remaining its function unknown in Arabidopsis according to the TAIR database (gene model AT3G58600.1). The NECAP-1 directs binding to the globular ear domain of the α-adaptin subunit (α-ear) of the adaptor protein 2 (AP-2) complex [43]. The NECAP-1 is involved in endocytosis and is a membrane protein. In ‘Williams’ the expression is more stable during the four time points (Fig 7). Therefore, at 9 DPI ‘Calcutta-4’ shows an induction when compared with ‘Williams’, while at 15 DPI is down-regulated (Fig 8).

The *PUK* showed up-regulation at 9 DPI of *M*. *fijiensis* in ‘Calcutta-4’, while at 6, 12 and 15 DPI is more stable (Fig 6). In ‘Williams’ the expression is more stable although a 2-fold up-regulation was observed at 9 DPI (Fig 7). However, at 9 DPI ‘Calcutta-4’ shows an induction when compared with ‘Williams’, while at 15 DPI is down-regulated (Fig 8). The function of the gene includes adenyl atecyclase activity, kinase activity, phosphotransferase activity, alcohol group as acceptor, and ATP binding. The *PUK* is involved in biosynthetic process, cAMP biosynthetic process, and metabolic process in *Arabidopsis thaliana* (TAIR, gene model AT1G26190.1). This gene is expressed under abscisic acid (ABA) application. Furthermore, ABA is a stress related hormone that could be used for drought tolerance [44].

The *UGP* showed up regulation at 9 DPI of *M*. *fijiensis* in ‘Calcutta-4’ (>2- and >1- fold induction), while at 6 and 12 DPI showed a stable expression while at 15 DPI is down-regulated, respectively (Fig 6). In ‘Williams’, the pattern of expression is similar to the PUK. Therefore, when compared the pattern of expression in ‘Calcutta-4’ with ‘Williams’ is similar, although at 15 DPI is down-regulated. The enzyme plays a central role as a glucosyl donor in cellular metabolic pathways. In Arabidopsis, the gene is down-regulated at drought stress (TAIR, locus AT3G03250.1). In populous, *UGPs* are differentially expressed at the tissue level and in response to metabolic feedback (sucrose) and cold stress [45]. However, two UGPs might be controlled at the postranscriptional/translational level leading to distinct roles.

Nucleotide-binding site leucine-rich repeat (NBS-LRR) encoding genes are present in diverse plant species and some prove to be R- genes [46, 47]. In this study, ‘Calcutta-4’ showed one NBS-LRR gene that was expressed (RGA1). However, NBS-LRR genes are also present in susceptible banana cultivars to Black Sigatoka including ‘Grand Naine’ [48]. Although, the expression of NBS-LRR-like genes in ‘Calcutta-4’ upon infection of *M*. *fijiensis*, may be indicative of functionality for resistant, further studies need to be performed. Furthermore, the most abundant class of plant disease resistant R-gene families expressed in the *Musa* spp.—*M*. *musicola* interaction were the NBS-LRR [14]. A total of 14 expressed NBS-LRR genes were identified in *M*. *musicola* inoculated and mock inoculated ‘Calcutta-4’ plants, while 25 were identified in ‘Grande Naine’ [14]. Additionally, the NBS-LRR EST expressed in ‘Calcutta-4’ in this study is present in the ‘Calcutta-4’ and ‘Grand Naine’ transcriptome in the *Musa* spp.—*M*. *musicola* interaction [14]. The up-regulation of the *RGA1* is only observed at 12 DPI when compared with ‘Williams’ while at the other time points the gene is down-regulated in the resistant than in the susceptible banana (Fig 8).

### Pattern of expression in a hypersensitive response

The level of expression of selected ESTs may revealed a pattern that is similar to that encountered by Cavalcante *et al*. [49] in ‘Calcutta-4’ after analysis for the presence of H_2_O_2_ in stomata, and peroxidise activity in guard and subsidiary cells, where a peak was encountered at 10 DPI of *M*. *fijiensis* on detached leaves. The followed days, a decreasing of the activity was found. This pattern is also consistent in the ‘Calcutta-4’ and *M*. *musicola* study where abundance of transcripts of genes involved in ROS detoxification and hypersensitive response was encountered [14].

## Conclusions

The genes identified in early disease development in a plant-pathogen interaction may be relevant for the resistant response of ‘Calcutta-4’ to Black Sigatoka. Genes with different functions may play a role in plant response to the disease. The published reference genome of ‘DH-Pahang’ facilitated the annotation and mapping of identified gene and common sequences were encountered in other banana—*M*. *musicola* interaction. As with other studies, this SSH library contributes to the elucidation of *Musa*-pathogen interaction in susceptible and resistant plants. The present study suggests a fine up regulation of these genes might be needed to perform an incompatible interaction. Further gene functional studies need to be performed to validate their use as candidate resistance genes in susceptible banana cultivars.

## Supporting Information

S1 File EST sequences. (PDF)

S1 Table Sequence similarity search of the ESTs obtained from the cDNA subtracted library at the banana genome sequence using blastn [22]. (PDF)

## Abbreviations

BSD: Black Sigatoka Disease
SSH: Suppression subtractive hybridization
DPI: Days post inoculation
chr: Chromosome
RGA1: Putative disease resistance protein RGA1
PUK: Phosphoribulokinase/Uridine kinase family protein gene
UGP: UTP—glucose-1-phosphate uridylyltransferase gene
UCP: Uncharacterized protein
ROS: Reactive oxygen species
NBS-LRR: Nucleotide-binding site leucine-rich repeat
INIBAP: International Network for the Improvement of Banana and Plantain
FB: First bioassay
SB: Second bioassay
KEGG: Kyoto Encyclopaedia of Genes and Genomes

## References

[1] FAOSTAT [Internet]. Rome, Economic and Social Development Department; [cited 2014 Nov 21]. Available from: http://faostat3.fao.org/home/E

[2] UNCTAD [Internet]. Geneva, Special Unit on Commodities–UNCTAD; [upadated 2012 Sep 5; cited 2014 Nov 1]. Available from http://www.unctad.info/en/Infocomm/AACP-Products/COMMODITY-PROFILE—Banana/.

[3] Churchill ACL. *Mycosphaerella fijiensis*, the black leaf streak pathogen of banana: progress towards understanding pathogen biology and detection, disease development, and the challenges of control. Mol Plant Pathol. 2011;12:307–328. 10.1111/j.1364-3703.2010.00672.x 21453427 PMC6640443

[4] Ploetz R. Black Sigatoka. Pesticide Outlook 2000;11:19–23.

[5] Lapeyre de Bellaire L, Fouré E, Abadie C, Carlier J. Black leaf streak disease is challenging the banana industry. Fruits 2010;65:327–342.

[6] Lepoivre P, Busogoro JP, Etame JJ, El-Hadrami A, Carlier J, Harelimana G, et al. Banana-Mycosphaerella fijiensis interactions. In: Jacome L, Lepoivre P, Marin D, Ortiz R, Romero R, Escalant JV, editors. Mycosphaerella leaf spot diseases of bananas: present status and outlook: Proceeding of the 2nd International workshop on Mycosphaerella leaf spot diseases; 2002 May 20–23; San Jose, Costa Rica. Montpellier: The International Network for the Improvement of Banana and Plantain; 2003. p. 151–159.

[7] Quinones W, Escobar G, Echeverri F, Torres F, Rosero Y, Arango V, et al Synthesis and antifungal activity of *Musa* phytoalexins and structural analogs. Molecules 2000; 5: 974–980.

[8] Hoss R, Helbig J, Bochow H. Function host and fungal metabolites in resistance response of banana and plantain in the black sigatoka disease pathosystem (*Musa* spp. -*Mycosphaerella fijiensis*). J Phytopathol. 2000,148:387–394.

[9] Ortiz R, Vuylsteke D. Inheritance of black sigatoka resistance in plantain-banana (Musa spp.) hybrids. Theor Appl Genet.1994; 89: 146–152. 10.1007/BF00225134 24177821

[10] Van Den Berg N, Crampton BG, Hein I, Birch PRJ, Berger DK. High-throughput screening of suppression subtractive hybridisation cDNA libraries using DNA microarray analysis. Biotechniques 2004; 37: 818–824. 15560137 10.2144/04375RR02

[11] Li CY, Deng GM, Yang J, Viljoen A, Jin Y, Kuang RB, et al Transcriptome profiling of resistant and susceptible Cavendish banana roots following inoculation with *Fusarium oxysporum* f. sp. *cubense* tropical race 4. BMC Genomics. 2012; 13:374 10.1186/1471-2164-13-374 22863187 PMC3473311

[12] Wang Z, Zhang JB, Jia CH, Liu JH, Li YQ, Yin XM, et al De Novo characterization of the banana root transcriptome and analysis of gene expression under *Fusarium oxysporum* f. sp. *Cubense* tropical race 4 infection. BMC Genomics. 2012; 13:650 10.1186/1471-2164-13-650 23170772 PMC3534498

[13] Swarupa V, Ravishankar KV, Rekha A. Characterization of tolerance to *Fusarium oxysporum* f.sp., *cubense* infection in banana using suppression subtractive hybridization and gene expression analysis. Physiol Mol Plant Pathol. 2013; 83:1–7.

[14] Passos MAN, de Cruz VO, Emediato FL, de Teixeira CC, Azevedo VCR, Brasileiro ACM, et al Analysis of the leaf transcriptome of Musa acuminata during interaction with *Mycosphaerella musicola*: Gene assembly, annotation and marker development. BMC Genomics. 2013; 14:78 10.1186/1471-2164-14-78 23379821 PMC3635893

[15] Portal O, Izquierdo Y, De Vleesschauwer D, Sánchez-Rodríguez A, Mendoza-Rodríguez M, Acosta-Suárez M, et al Analysis of expressed sequence tags derived from a compatible *Mycosphaerella fijiensis*-banana interaction. Plant Cell Rep. 2011; 30: 913–928. 10.1007/s00299-011-1008-z 21279642

[16] Passos MAN, de Cruz VO, Emediato FL, Teixeira CC, Souza MT Jr, Matsumoto T, et al Development of expressed sequence tag and expressed sequence tag–simple sequence repeat marker resources for *Musa acuminata*. AoB Plants. 2012; pls030. 10.1093/aobpla/pls030 PMC3521319 23240072

[17] Diatchenko L, Lau YF, Campbell AP, Chenchik A, Moqadam F, Huang B, et al Suppression subtractive hybridization: a method for generating differentially regulated or tissue-specific cDNA probes and libraries. Proc Natl Acad Sci U S A. 1996; 93: 6025–6030. 8650213 10.1073/pnas.93.12.6025 PMC39182

[18] Ji W, Wright MB, Cai L, Flament A, Lindpaintner K. Efficacy of SSH PCR in isolating differentially expressed genes. BMC Genomics. 2002; 3:12 12033988 10.1186/1471-2164-3-12 PMC115870

[19] Jiménez M, Van der Veken I, Neirynck H, Rodriguez H, Ruiz O, Swennen R. Organic banana production in Ecuador: its implications on black Sigatoka development and plant-soil nutritional status. Renew Agric Food Syst. 2007; 22:297–306.

[20] Alvarado-Capó Y, Leiva-Mora M, Dita-Rodríquez MA, Acosta M, Cruz M, Portal N, et al. Early evaluation of black leaf streak resistance by using mycelial suspensions of *Mycosphaerella fijiensis* In: Jacome L, Lepoivre P, Marin D, Ortiz R, Romero R, Escalant JV, editors. *Mycosphaerella* leaf spot diseases of bananas: present status and outlook: Proceeding of the 2^nd^ International workshop on *Mycosphaerella* leaf spot diseases; 2002 May 20–23; San Jose, Costa Rica. Montpellier: The International Network for the Improvement of Banana and Plantain; 2003. p. 169–175.

[21] Wiame I, Remy S, Swennen R, Sági L. Irreversible DNase I heat inactivation without RNA degradation. Biotechniques. 2000; 29: 252–256. 10948426 10.2144/00292bm11

[22] The Banana Genome Hub [Internet]. Montpellier: Cirad, Bioversity International and supported by the South Green Bioinformatics platform; [cited 2015 Jan 25]. Available from: http://banana-genome.cirad.fr/blast.

[23] Genome Net [Internet]. Kyoto: Kyoto University Bioinformatics Center; [cited 2015 Jan 19]. Available from: http://www.genome.jp/tools/blast/.

[24] EMBL-EBI [Internet]. Hinxton: European Molecular Biology Laboratory; [cited 2015 Jan 19]. Available from: http://www.ebi.ac.uk/interpro/.

[25] The Banana Genome Hub [Internet]. Montpellier: Cirad, Bioversity International and supported by the South Green Bioinformatics platform; [cited 2015 Jan 25]. Available from http://banana-genome.cirad.fr/musacyc.

[26] Vandesompele J, De Preter K, Pattyn F, Poppe B, Van Roy N, De Paepe A, et al Accurate normalization of real-time quantitative RT-PCR data by geometric averaging of multiple internal control genes. Genome Biol. 2002; 18: 7. 10.1186/gb-2002-3-7-research0034 PMC126239 12184808

[27] Andersen C L, Jensen JL, Ørntoft TF. Normalization of real-time quantitative reverse transcription-PCR data: a model-based variance estimation approach to identify genes suited for normalization, applied to bladder and colon cancer data sets. Cancer Res. 2004; 64: 5245–5250. 15289330 10.1158/0008-5472.CAN-04-0496

[28] Podevin N, Krauss A, Henry I, Swennen R, Remy S. Selection and validation of reference genes for quantitative RT-PCR expression studies of the non-model crop. Mol Breed. 2012; 30: 1237–1252. 23024595 10.1007/s11032-012-9711-1 PMC3460175

[29] Beveraggi A, Mourichon X, Salle G. Etude des interactions hôte-parasite chez des bananiers sensibles et résistants inoculés par *Cercospora fijiensis* (*Mycosphaerella fijiensis*) responsable de la maladie des raies noires. Can J Bot. 1995; 73: 1328–1337.

[30] Rojas CM, Senthil-Kumar M, Tzin V, Mysore KS. Regulation of primary plant metabolism during plant-pathogen interactions and its contribution to plant defense. Front Plant Sci. 2014; 5:17 10.3389/fpls.2014.00017 24575102 PMC3919437

[31] Ibrahim HMM, Hosseini P, Alkharouf NW, Hussein EHA, Gamal El-Din AEKY, Aly MAM, et al Analysis of Gene expression in soybean (*Glycine max*) roots in response to the root knot nematode *Meloidogyne incognita* using microarrays and KEGG pathways. BMC Genomics. 2011; 12: 220 10.1186/1471-2164-12-220 21569240 PMC3225080

[32] Jones AME, Thomas V, Bennett MH, Mansfield J, Grant M. Modifications to the Arabidopsis defense proteome occur prior to significant transcriptional change in response to inoculation with *Pseudomonas syringae*. Plant Physiol. 2006; 142: 1603–1620. 17028151 10.1104/pp.106.086231 PMC1676056

[33] Kumar S, Wang Z, Banks TW, Jordan MC, McCallum BD, Cloutier S. Lr1-mediated leaf rust resistance pathways of transgenic wheat lines revealed by a gene expression study using the Affymetrix GeneChip® Wheat Genome Array. Mol Breed. 2014; 34: 127–141.

[34] Boddu J, Cho S, Muehlbauer GJ. Transcriptome analysis of trichothecene-induced gene expression in barley. Mol Plant Microbe Interact. 2007; 20: 1364–1375. 17977148 10.1094/MPMI-20-11-1364

[35] Wong HL, Sakamoto T, Kawasaki T, Umemura K, Shimamoto K. Down-regulation of metallothionein, a reactive oxygen scavenger, by the small GTPase OsRac1 in rice. Plant Physiol. 2004; 135: 1447–1456. 15220467 10.1104/pp.103.036384 PMC519061

[36] Akimoto-Tomiyama C, Sakata K, Yazaki J, Nakamura K, Fujii F, Shimbo K, et al Rice gene expression in response to N-acetylchitooligosaccharide elicitor: comprehensive analysis by DNA microarray with randomly selected ESTs. Plant Mol Biol. 2003; 52:537–551. 12956525 10.1023/a:1024890601888

[37] Hirao T, Fukatsu E, Watanabe A. Characterization of resistance to pine wood nematode infection in *Pinus thunbergii* using suppression subtractive hybridization. BMC Plant Biol. 2012; 12: 13 10.1186/1471-2229-12-13 22272988 PMC3398268

[38] Miller RNG, Bertioli DJ, Baurens FC, Quirino BF, Ciampi AY, Santos CMR, et al Understanding plant responses to biotic stress: Ongoing research in *Musa*. Acta Hortic. 2009; 828: 255–272.

[39] Van Den Berg N, Berger DK, Hein I, Birch PRJ, Wingfield MJ, Viljoen A. Genes up-regulated in tolerant cavendish banana roots in response to *Fusarium oxysporum* f. sp. *cubense* infection. Acta Hortic. 2009; 828: 273–282.

[40] Zhou T, Yang X, Wang L, Xu J, Zhang X. GhTZF1 regulates drought stress responses and delays leaf senescence by inhibiting reactive oxygen species accumulation in transgenic Arabidopsis. Plant Mol Biol. 2014; 85: 163–77. 10.1007/s11103-014-0175-z 24473898

[41] Sreedharan S, Shekhawat UKS, Ganapathi TR. MusaSAP1, a A20/AN1 zinc finger gene from banana functions as a positive regulator in different stress responses. Plant Mol Biol. 2012; 80: 503–517. 10.1007/s11103-012-9964-4 22961664

[42] Chen J, Chen JY, Wang JN, Kuang JF, Shan W, Lu WJ. Molecular characterization and expression profiles of MaCOL1, a CONSTANS-like gene in banana fruit. Gene. 2012; 496: 110–117. 22285923 10.1016/j.gene.2012.01.008

[43] Ritter B, Philie J, Girard M, Tung EC, Blondeau F, McPherson PS. Identification of a family of endocytic proteins that define a new α-adaptin ear-binding motif. EMBO Rep. 2003; 4: 1089–1095. 14555962 10.1038/sj.embor.7400004 PMC1326374

[44] Dias MC, Oliveira H, Costa A, Santos C. Improving elms performance under drought stress: The pretreatment with abscisic acid. Environ Exp Bot. 2014; 100: 64–73.

[45] Meng M, Geisler M, Johansson H, Mellerowicz EJ, Karpinski S, Kleczkowski LA. Differential tissue/organ-dependent expression of two sucrose- and cold-responsive genes for UDP-glucose pyrophosphorylase in Populus. Gene. 2007; 389: 186–195. 17196771 10.1016/j.gene.2006.11.006

[46] Meyers BC, Dickerman AW, Michelmore RW, Sivaramakrishnan S, Sobral BW, Young ND. Plant disease resistance genes encode members of an ancient and diverse protein family within the nucleotide-binding superfamily. Plant J. 1999; 20: 317–332. 10571892 10.1046/j.1365-313x.1999.t01-1-00606.x

[47] Van Der Biezen EA, Freddie CT, Kahn K, Parker JE, Jones JD. Arabidopsis RPP4 is a member of the RPP5 multigene family of TIR-NB-LRR genes and confers downy mildew resistance through multiple signalling components. Plant J. 2002; 29: 439–451. 11846877 10.1046/j.0960-7412.2001.01229.x

[48] Miller RNG, Bertioli DJ, Baurens FC, Santos CM, Alves PC, Martins NF, et al Analysis of non-TIR NBS-LRR resistance gene analogs in *Musa acuminata* Colla: Isolation, RFLP marker development, and physical mapping. BMC Plant Biol. 2008; 8:15 10.1186/1471-2229-8-15 18234103 PMC2262081

[49] Cavalcante MJB, Escoute J, Madeira JP, Romero RE, Nicole MR, Oliveira LC, et al Reactive oxygen species and cellular interactions between *Mycosphaerella fijiensi*s and banana. Tropical Plant Biol. 2011; 4: 134–143.

