# Supplementary material for: Identification of Differentially-Expressed Genes in Response to Mycosphaerella fijiensis in the Resistant Musa Accession ‘Calcutta-4’ Using Suppression Subtractive Hybridization
Source: PLoS One. 2016 Aug 3;11(8):e0160083. doi: 10.1371/journal.pone.0160083 (PMC4972352; doi:10.1371/journal.pone.0160083)
Supplement: S1 File — (PDF) [file pone.0160083.s001.pdf]

S1 File. EST sequences.

TYPE: EST

STATUS: New

CONT_NAME: Santos E

CITATION: Identification of differentially expressed genes in response to Mycosphaerella fijiensis in the resistant Musa accession Calcutta 4 using suppression subtractive hybridization.

LIBRARY: SSH Calcutta M. fijiensis library

EST#: 1A1 BMCIBE

DNA_TYPE: cDNA

PUBLIC:

COMMENT: Raw sequences were clear up, from vector and cloning adaptors, with the program CLC Genomics Workbench (CLC bio, Denmark).

SEQUENCE:

CGAAGCCGCCCTGGAGTCGGCGCTGCTCG

CTGCCGCCGCGGCGAGTTTCACTTCAGGATACATCTTCGCGTTGCACGCCTCTCCACCAA

CTACATCACAAGCCTCCACCGGAAACACAGAGGTGGAGGAGCTGTAATCGATGGTCGAGG

ACTCTGGTGCGGTGACGGCGACCACCCTGGACTTGTGGAAGGTCCTGCGTGGCTTGGATG

ACCTGGGCGCGATTGCCTTGGTGCTCCTGAGGGGC

||

TYPE: EST

STATUS: New

CONT_NAME: Santos E

CITATION: Identification of differentially expressed genes in response to Mycosphaerella fijiensis in the resistant Musa accession Calcutta 4 using suppression subtractive hybridization.

LIBRARY: SSH Calcutta M. fijiensis library

EST#: 1A3 BMCIBE

DNA_TYPE: cDNA

PUBLIC:

COMMENT: Raw sequences were clear up, from vector and cloning adaptors, with the program CLC Genomics Workbench (CLC bio, Denmark).

SEQUENCE:

GGAGGCAGCCGCGAAGAGGAAGATGATACTGAGGCTGGACTACGAGGCTGTCATCGCCGC

TTGCTCTTCTAATGTGTCGTCGCTGTGGATAGATGGGGTGAGACCGGATTTCGATCCCAA

CGATTCATGGCCGGACGTCCCAGCGATGGCAAGAGGAGCAGCAGCGATGAAGGAGATGCA

TTATCAACAGGAGGTGGTTCACGCGGCGGACGAAGGAAGGGAGGCGAGGGTGTCGAGGT

||

TYPE: EST

STATUS: New

CONT_NAME: Santos E

CITATION: Identification of differentially expressed genes in response to Mycosphaerella fijiensis in the resistant Musa accession Calcutta-4 using suppression subtractive hybridization.

LIBRARY: SSH Calcutta M. fijiensis library

EST#: 1A7 BMCIBE

DNA_TYPE: cDNA

PUBLIC:

COMMENT: Raw sequences were clear up, from vector and cloning adaptors, with the program CLC Genomics Workbench (CLC bio, Denmark).

SEQUENCE:

ACGATGCCCGGCCTTCCAACTCGACCTTGTTTCTTCGACATTGACATTGATATCGCTACG

GGGAAAGTCCTCGGCCTCTCCTAGAATGTCATGTAAGTTTATATTTCCCGATTGTTGTTT

CCTTAGTTCAAGTCCCAGCGTAAAAAGAGTCCAAGAAAGTTGAAGAACATTCGCTTCATT

AAACTTGGGATGCGTCAAATAACTGCTACAATGT

||

TYPE: EST

STATUS: New

CONT_NAME: Santos E

CITATION: Identification of differentially expressed genes in response to Mycosphaerella fijiensis in the resistant Musa accession Calcutta 4 using suppression subtractive hybridization.

LIBRARY: SSH Calcutta M. fijiensis library

EST#: 1B3 BMCIBE

DNA_TYPE: cDNA

PUBLIC:

COMMENT: Raw sequences were clear up, from vector and cloning adaptors, with the program CLC Genomics Workbench (CLC bio, Denmark).

SEQUENCE:

CACCATATCCCCACTCATCCATGCCAACTTTTTGAGCACAAGCGTTTAGTGATGCCAGCA

TATACTTATCATAACACTGAAATTCTCCTAGCTCGGCTGCAGCCCTTGCTCGAATTAACT

TCTCAGATGGGCAAGAAGGATACCTAAGTTCACCAGCAGGGCCCATACCAACTTGAATTT

CCGTTATTACAACTCGTAAGAAGTCCCTGAATGTATCCCTGAAACTCCTCATAAAATCTG

AATAAGCCTGGATTGGAGATCGTCCCCTTAGAACAGGAAAAACATCACATCCCAAAGAGA

TGT

||

TYPE: EST

STATUS: New

CONT_NAME: Santos E

CITATION: Identification of differentially expressed genes in response to Mycosphaerella fijiensis in the resistant Musa accession Calcutta 4 using suppression subtractive hybridization.

LIBRARY: SSH Calcutta M. fijiensis library

EST#: 1B4 BMCIBE

DNA_TYPE: cDNA

PUBLIC:

COMMENT: Raw sequences were clear up, from vector and cloning adaptors, with the program CLC Genomics Workbench (CLC bio, Denmark).

SEQUENCE:

CAAGACATCATTTAGCAACCGGCACTGCTCATTGTGCCGAGTCCCTTGGTGAGGCTGAGA

TCCATTTTGCAGCGAGCCCTGTTGGCAATAGAAACACCATTGGTGCCTCAAGAGGTGATT

CAAGCCACATGGAAACATCCAAAATCGGATGGGACTTGTCGCTCCGATTGGGACTGCCTT

CACCTCCATCCTTCGAAGAAAGT

||

TYPE: EST

STATUS: New

CONT_NAME: Santos E

CITATION: Identification of differentially expressed genes in response to Mycosphaerella fijiensis in the resistant Musa accession Calcutta 4 using suppression subtractive hybridization.

LIBRARY: SSH Calcutta M. fijiensis library

EST#: 1B5 BMCIBE

DNA_TYPE: cDNA

PUBLIC:

COMMENT: Raw sequences were clear up, from vector and cloning adaptors, with the program CLC Genomics Workbench (CLC bio, Denmark).

SEQUENCE:

ACTACTACTGTATTCTCTTTGTGAGAGTATGATTGCGAGCAACTGATGATTTGATCAGGT

AATTTCTCCTTTCTTTCTATAAGCTTATGAGCATGCATTAATTGCTTGGATTTATATATT

GCTTATGTGAATTTTCACCTGCTTTATTTATGATGATATCATTCTCTTTGTGTTTCATCT

GCCTATATAGAGGAAGCTATTGATGCATGAAATAAATTAGAGAAGCAGGATATATGATAA

CATCATGAACAACTTTGTTTCACTATGAACATTCTCAAAGATGATAGTGTGATTGCTTTT

TCTTTCCCCAAAATACACCAAAAATGGGTTCAGATATGGAGAAATCATCATCCTGCCTAC

TTGATATGATCCAAACAAAAACTTGGCACCTTAGCTACTTTTTTGGAGTAGCTTGAAGTA

TCGGAAAAAGGCGAAAAACTACTATGAGTTTTCAGCTCTAGTTTAATTGATAGATCCCAT

GATCTACAGTTTGGTTATCCTCCTGGATGAACAATGTAATCTCTAACCTTCTCTCTATTC

TTGATAAGAATGCTTACAAGTCAGATTGTCAAACATGTGTATTGTGCTAAAAAAAAAAAA

AAAAAAAAAAAAAAAAAAAAAAAGCTTGT

||

TYPE: EST

STATUS: New

CONT_NAME: Santos E

CITATION: Identification of differentially expressed genes in response to Mycosphaerella fijiensis in the resistant Musa accession Calcutta 4 using suppression subtractive hybridization.

LIBRARY: SSH Calcutta M. fijiensis library

EST#: 1B9 BMCIBE

DNA_TYPE: cDNA

PUBLIC:

COMMENT: Raw sequences were clear up, from vector and cloning adaptors, with the program CLC Genomics Workbench (CLC bio, Denmark).

SEQUENCE:

ACTGAACCCTCCCTGATGCCGAGAGCCTGTCGAACAGTATCATTGTTTGCCCAGGCGTGG

GAGAGCAAATACCAATAATTCCTACACAAAGGATCATTAGTGTTCGACGCAGGGAGCTCG

GAAAGTGCTTCGAGTAACCTCCTCCTGTCGGCAGTGATTATGTTGGGCTTCGGGCTTTCA

GGGAAGCTGCACTGAGGCTCCAGAATCTGGGCAGCGTTAATCCCACTGAGATCCTGCATG

TTCATAATGTTCTCTGTTTTACTGTATCCACCCACCTTGCACACTC

||

TYPE: EST

STATUS: New

CONT_NAME: Santos E

CITATION: Identification of differentially expressed genes in response to Mycosphaerella fijiensis in the resistant Musa accession Calcutta 4 using suppression subtractive hybridization.

LIBRARY: SSH Calcutta M. fijiensis library

EST#: 1B10 BMCIBE

DNA_TYPE: cDNA

PUBLIC:

COMMENT: Raw sequences were clear up, from vector and cloning adaptors, with the program CLC Genomics Workbench (CLC bio, Denmark).

SEQUENCE:

AGAAATGCGACCATGCAAAAATGTATGAGAAAATTTCGGACGAGAACTTACAGCTGATGC

GTGAGAGGCTAATGGAGACCGTCATTTGGCCATCAGATGACACAAACATGGAGAAAATTG

ATTGATTCGCAACGTGTGTGTGTGTGTGTGCTCACCTTAGTTTGTAGTTTAGCAAAATCA

AATCTTTATGTCCCGTTCCGATGGTTTCTGTATATAATGTTTTACGATCTGATTATAGAA

ATATAGGTCTCGGTCATGCGACCTGTTTAAGCACGAAAAAAAAAAAAAAAAAAAAAAAAA

AAGCTTGT

||

TYPE: EST

STATUS: New

CONT_NAME: Santos E

CITATION: Identification of differentially expressed genes in response to Mycosphaerella fijiensis in the resistant Musa accession Calcutta 4 using suppression subtractive hybridization.

LIBRARY: SSH Calcutta M. fijiensis library

EST#: 1B12 BMCIBE

DNA_TYPE: cDNA

PUBLIC:

COMMENT: Raw sequences were clear up, from vector and cloning adaptors, with the program CLC Genomics Workbench (CLC bio, Denmark).

SEQUENCE:

ACTCCTCCTCATCAGATATCTCAAGCCTAGGCTTCTTAAACAAAGACCCACACTTGTTGC

CTCTGTAGAATCCTGTATCACTAGCTTGTGGAACAGGGAATTTGTTGGCTCTGTAGAAGC

CTGTGTTGCTTGCTTGTGTGGTGGGGAGTTCAGGGACTTGAGCTGCTGTTTGGGTCGCCT

TGGGCAGTTGGTCATGGAAAAGACCAATGTCTGCAAACCAATCCAACTCCTCATATCCTA

CAGGAGACCCCTTGTAACCAGTTTCTTGGTCATAAAGTTGAAGGAACTCATCAGCAGCCC

ATGCCGAGTCCATGAATGATGAAATGGTTTGTGGTGTTTCAGGAACTTTTGCGGCTGGTG

TG

||

TYPE: EST

STATUS: New

CONT_NAME: Santos E

CITATION: Identification of differentially expressed genes in response to Mycosphaerella fijiensis in the resistant Musa accession Calcutta 4 using suppression subtractive hybridization.

LIBRARY: SSH Calcutta M. fijiensis library

EST#: 1C2 BMCIBE

DNA_TYPE: cDNA

PUBLIC:

COMMENT: Raw sequences were clear up, from vector and cloning adaptors, with the program CLC Genomics Workbench (CLC bio, Denmark).

SEQUENCE:

ATGTGAGTATCGCTGCAAAGTCTGGAGTAAAGTTGGAGATCTCTGATGGAGCTGTCCTCG

AGAACAAGGTGATCAATGGCCCCGAAGATATCTGAGCATATTCACGAAATTTTGGCGTGT

AATTTGGGCTGCTCCATGAGAGGCTTTGGATTCCCTCTAGAGAGGTTTTGACTCTCACTC

TTGAGCATTTTACCCTCTTTGTTCTCAACTCATGAAATGTGAGCATGTTTTACTCAAAAC

AAAGAGAAAACTCCTGCATTTACATTGTTGTTTTCTCGCTGCCGTGAGATGCAGCATATA

ATGTTGTGAAACCAAATTTTTAAAAAAAAAAAAAATAAAAAAAAAAAAAAAAAAAAAAAA

AAAGCTTGT

||

TYPE: EST

STATUS: New

CONT_NAME: Santos E

CITATION: Identification of differentially expressed genes in response to Mycosphaerella fijiensis in the resistant Musa accession Calcutta 4 using suppression subtractive hybridization.

LIBRARY: SSH Calcutta M. fijiensis library

EST#: 1C3 BMCIBE

DNA_TYPE: cDNA

PUBLIC:

COMMENT: Raw sequences were clear up, from vector and cloning adaptors, with the program CLC Genomics Workbench (CLC bio, Denmark).

SEQUENCE:

CCTAGCTCACGTGAAAAATCTGAAGCATCTTCTACCCAACACCAAGCTGGTGGCCTAGGG

ACAACTTCTTTTCCNNCGTCAGGGTTTCCAGCGAATCCTTTTGATTTTTCTTCTATGCAG

AACCTCCTTAACGATCCTACAATTAAAGAAATGCCTGAGCANATTGCTAAGGACCCTGTA

TTCANTCAAATGGTTGAACAGCTTCAAAAGATTGTCCATAGCGTATGTCAAGAAAGTGTC

CCTCCATTGGATCCTCAACAATACGTTTCAACGATGCAACAAGTCATGCAAAATCCTCAG

TTCATGAATATGGATGAACGCCTTGTAAATGCTATCATGCATGATCNTGGAATGTCTTCA

ACGCTTGACATTTTGGCAAATCCTGCTCAAAAAGAACACATTGAAGAACTAATGGCTCGC

ATGAAGGATGATCCATCCCTGAANACAATTCTTGAAGANATACAAAGTGGAGGTCCCTCT

GCAATGATGAAGT

||

TYPE: EST

STATUS: New

CONT_NAME: Santos E

CITATION: Identification of differentially expressed genes in response to Mycosphaerella fijiensis in the resistant Musa accession Calcutta 4 using suppression subtractive hybridization.

LIBRARY: SSH Calcutta M. fijiensis library

EST#: 1C7 BMCIBE

DNA_TYPE: cDNA

PUBLIC:

COMMENT: Raw sequences were clear up, from vector and cloning adaptors, with the program CLC Genomics Workbench (CLC bio, Denmark).

SEQUENCE:

GGGCCTCGACGACGAGGAACGGGGTAGGAACAGCATCTGGGAGCCGATGCTGTTGACGGC

AGCCATCGATGGATAAGATGGAAGAAGGGATTCGCTCTCGGAAGAAAGGGCTGAGAGATC

GG

||

TYPE: EST

STATUS: New

CONT_NAME: Santos E

CITATION: Identification of differentially expressed genes in response to Mycosphaerella fijiensis in the resistant Musa accession Calcutta 4 using suppression subtractive hybridization.

LIBRARY: SSH Calcutta M. fijiensis library

EST#: 1C8 BMCIBE

DNA_TYPE: cDNA

PUBLIC:

COMMENT: Raw sequences were clear up, from vector and cloning adaptors, with the program CLC Genomics Workbench (CLC bio, Denmark).

SEQUENCE:

ACTATGCAACAACCAAATCTCTCCTCGATGATGTTCTTGCAAAGATAGGCTAGAGGGTCG

ATGTCTGAACTATTCTTCGGAATTCTTATCTCAAGAAAGATATCATCAGTGATGTAAGCT

TCCATCTTTGTCCAGTTTGCATCTTCGGGCAGCTCGAGACGCCTAGCAAATCCATACTCC

CACCAGCGGCCATTTTTCCAGTCCTTTGGATCAGTTTCTCGCCCTCTCCACCGCCCACTG

ATCTCGACCACCATTTCCTTCAGGTTGCATACATCTATATCACATTTTCTTGCACCAACT

GAAACAAACATGAAGCTCTTAAGATATTGTATAGCTTAATCAGATTCATGAACATTTAGA

CGGATCGGAGGCATCTGAATACTTGTTTGTCCTCTGTTAATCTTCAAGTAGAGAAGTTTT

TGGAGAAGGTTATCATCATACATGT

||

TYPE: EST

STATUS: New

CONT_NAME: Santos E

CITATION: Identification of differentially expressed genes in response to Mycosphaerella fijiensis in the resistant Musa accession Calcutta 4 using suppression subtractive hybridization.

LIBRARY: SSH Calcutta M. fijiensis library

EST#: 1C9 BMCIBE

DNA_TYPE: cDNA

PUBLIC:

COMMENT: Raw sequences were clear up, from vector and cloning adaptors, with the program CLC Genomics Workbench (CLC bio, Denmark).

SEQUENCE:

TTTTTTTTTTTCCAGCAATTTGATCCATATGAGAATTATTGAATAACTATACAAATCCTT

GTGCTTCGCAGTTGCTGACCTCATGTTATTTGCAGCAAAAGGTCCCTAACCTTACCTTAC

AAGATCGATCAAAACAACACATTATTAAGAATAAGAATTCAACAGCCATCAACCGATGAC

TCATGCCAGTAACGTGCCAAACAAATATTAGTGTGTGATCATAACGCTTAAAGGGAACAC

CTGTTTGTTTCCACAGTGACATATAGGGGCACGGAATATGACATAGCAATCATGTTTTCT

TAATCTGCCCGAGATTGACCAGCCCGAGAAGACAAGATGATTCCAACAATGAGACCATAG

AGAGCAAGGGCTTCCGCAAAAATGAAAATAAGAATCATGCCTACAAATAGCTTCGGCTGT

TGAGCGTTGGCCCTGACTCCGGCATCACCGACAATCCCAATCGCCATTCCGGCAGAGAGG

CCAGCGAGACCACAGGCGAGCCCCGAGGAGAGGTGCGCGT

||

TYPE: EST

STATUS: New

CONT_NAME: Santos E

CITATION: Identification of differentially expressed genes in response to Mycosphaerella fijiensis in the resistant Musa accession Calcutta 4 using suppression subtractive hybridization.

LIBRARY: SSH Calcutta M. fijiensis library

EST#: 1C10 BMCIBE

DNA_TYPE: cDNA

PUBLIC:

COMMENT: Raw sequences were clear up, from vector and cloning adaptors, with the program CLC Genomics Workbench (CLC bio, Denmark).

SEQUENCE:

ATCTATCTCACCCTGTTCCAGAACCGGATCTCATCCCACGGGATCCACTACCTGATGGCC

GGGCTGCTGCTCAGCAAGGCCCTCTACCTCGTCTTCGCCGCCGAGGACCAGCACTACATC

CGGCAGACCGGCACCCCGCACGGCTGGGACATCCCCTTCTACCTCTTCCAGTTCCTGAAA

GGCGTCCTCTTGTTCACCGTGATCGTCCTCATCGGCACCGGCTGGTCCTTCCTCAAGCCC

TTCCTCCAGGAGCGCGAGAAGAAGGTGCTAATGATCGTGATCCCACTTCAGATAATCGCC

AACATCGCCTCCGTGGTGATCGGCGAGACCGGACCCTTCATCAGGGATTGGGTCACCTGG

AACCAGGTGTTCCTCCTCATCGACATCATCTGCTGCTGCACCGTCCTGTTCCCCATCATC

TGGTCCATCAGATCTCTGCGTGAGACGTCAAAGACCGACGGAAAGGCCGCAAGGAACCTC

ATGAAGCTCACACTTTTCCGGCAATTCTACATCATTGTGATCGGATATTTGT

||

TYPE: EST

STATUS: New

CONT_NAME: Santos E

CITATION: Identification of differentially expressed genes in response to Mycosphaerella fijiensis in the resistant Musa accession Calcutta 4 using suppression subtractive hybridization.

LIBRARY: SSH Calcutta M. fijiensis library

EST#: 1D2 BMCIBE

DNA_TYPE: cDNA

PUBLIC:

COMMENT: Raw sequences were clear up, from vector and cloning adaptors, with the program CLC Genomics Workbench (CLC bio, Denmark).

SEQUENCE:

GCCGCGGCAACTAAGGTGTTTGCTCCCCGGTTGGCCCGCGGCGAGGCTGTTCTTGTGATT

CCATCCACAAATTTCTGCTGATAGCCTTCCAGCCCGAGCAGCTCGAGGTCGAAATCCAAC

TCTCTGGCACTCAATGACGCCTTGAGTCTGCTGCTCGGCAGCTGCAAGGCCGGAGGTGTC

ATCAAGCCGCCCGCTTGATTCATCCACGCCGCCGCAGCCCCTAAACCCGACGAAGCCGTA

GGGGACATCGGCGAGCCTGGCTGCTGCATCAGCGTCAAGGCCATCGCCATGTCCAAAGAG

GAAAGAGCCGGCGACGACGATCTTGGAGAGGACAACAACGTTCCCGCAACTGATGCCGCA

GAGGGGTTCACCGTCCGCAGCTCCTCCGGCTTGTGGGCAAAGAAGCAAACCCGGCGGTTG

CAGCATGNCTCATCCTTGCAGATCCG

||

TYPE: EST

STATUS: New

CONT_NAME: Santos E

CITATION: Identification of differentially expressed genes in response to Mycosphaerella fijiensis in the resistant Musa accession Calcutta 4 using suppression subtractive hybridization.

LIBRARY: SSH Calcutta M. fijiensis library

EST#: 1D8 BMCIBE

DNA_TYPE: cDNA

PUBLIC:

COMMENT: Raw sequences were clear up, from vector and cloning adaptors, with the program CLC Genomics Workbench (CLC bio, Denmark).

SEQUENCE:

ACAAGTGTAGGAGAATAAACATGCAAAGAGGCACGGAAGAAGACGATGACTGCTTTGGTA

AGGGAGTAGTGCAGTCTCAGACTTTGGCTGCCTCAGCAACAGCAGCTGCCCTCTCGGCAA

CGATCTGCTCATATAAGTCTCTTATCTTCAAACCGATAATGGTCTGGAAGAGGCCGGTGC

CGTTGTTGCTGCCGGGGAAGTCGGCGTGCTTCAGCATCTGCTGCGTCACCTCCCCGTAGA

ACTTAGTCGACAGGTTGCTCAGATGGCTCTCCACATAGATCAGCTCATCCAACCTGTCGA

ACTGCCCATCCATCTCCAACACCGAGACCTCGTTGGAGTAGTAGGTGTCCGGGCCGTAAG

AGAACTGGATGCCGGGGT

||

TYPE: EST

STATUS: New

CONT_NAME: Santos E

CITATION: Identification of differentially expressed genes in response to Mycosphaerella fijiensis in the resistant Musa accession Calcutta 4 using suppression subtractive hybridization.

LIBRARY: SSH Calcutta M. fijiensis library

EST#: 1D11 BMCIBE

DNA_TYPE: cDNA

PUBLIC:

COMMENT: Raw sequences were clear up, from vector and cloning adaptors, with the program CLC Genomics Workbench (CLC bio, Denmark).

SEQUENCE:

TCCCTGGTCCCCGGCCNCCTGGGCCGGGGAATTTTAAACCAACTCCTTTTGGGGCACCGT

GGTCGCCGCCAAGGTACTTGCTCCNGGGCCATGTATAAATGCAAGAACCGGAACTAGGGG

CCATCGCCGACCCTTCTATGAACTCAAACCTGAATCAGGTGCCTCCATATGGCCCTCCAA

AATGTTTAACAAGGCCACCGGNAACCGTCATATATCTACCATATTGCATAGTTTGCTACT

AAGACTCTGTTAGCTAAGATTCCCTANGATCATATAAGCTATTTATGATTGTGAATTTGG

TGTTTGTATGTCTGACTTGCTATTGTTGTATGTCTGAACATTTGGCTTGCTGTTTGCAAC

TGGTATAATTTAA

||

TYPE: EST

STATUS: New

CONT_NAME: Santos E

CITATION: Identification of differentially expressed genes in response to Mycosphaerella fijiensis in the resistant Musa accession Calcutta 4 using suppression subtractive hybridization.

LIBRARY: SSH Calcutta M. fijiensis library

EST#: 1E1 BMCIBE

DNA_TYPE: cDNA

PUBLIC:

COMMENT: Raw sequences were clear up, from vector and cloning adaptors, with the program CLC Genomics Workbench (CLC bio, Denmark).

SEQUENCE:

ACTTTGGTTGAAGAACCAAAACAAATCAGGGAAGCGCTGCAAAATTTACTGATGAGCATC

TCTTCTAAGGCAGAACCCACAAGTAGTGTGAATCCATTTTCTTTTCTTGACATCGTCCTT

TCAGTAAATATTGGCCCATAGCTATAACAAACACTTGGCTTTTGATGCTCGAAACCCATA

TAGACAAAAGGGGCCACAGGAATTTAGCTGGAGAGTGTTCTTGCAAGTGATGATAAATAC

TCAGAAAGAGGATCGTTCAAGTGGGGGCACAAATTCACCAAAATCTCCAATCTGCATGCC

CGGTGACCAATGTTGGCTTGTTGTATCAGGTCCTGTATGAGGAAGACCACGCATACCACC

AAAAACATCGTTATCAAAGTAAGGGATTGGATCAAGGATGTGATTCAGATTCTCCAAATC

GGGCCAATGACCAGATGGGTTTGGGGCAAATGGAGT

||

TYPE: EST

STATUS: New

CONT_NAME: Santos E

CITATION: Identification of differentially expressed genes in response to Mycosphaerella fijiensis in the resistant Musa accession Calcutta 4 using suppression subtractive hybridization.

LIBRARY: SSH Calcutta M. fijiensis library

EST#: 1E3 BMCIBE

DNA_TYPE: cDNA

PUBLIC:

COMMENT: Raw sequences were clear up, from vector and cloning adaptors, with the program CLC Genomics Workbench (CLC bio, Denmark).

SEQUENCE:

ACTATGGGCGATGCAAAACCTCCAACATTTGAGAAAAAAGGACAGTTTATAACTCATACA

TATTTCATCCATTTCGTATCTCTGCTGTAGATAAAGACCATATCCAATATGTTCTCAAAA

GCTCAAAATGCCGCCCACCCAGATGCTGTGCTCTTTGTTGATCCTGACCCAGTCGTCGAA

GGTAGGCTTCTCTCAATAGAAGAAAGATCTTGTAAAGAATCTCCAGGACGCCTTGCAGAG

TCTTTGGGAGGGTTCAGTCCAGAATTGTGACCTAAGGTCATCCTAGCAGTGGCAGGATCA

TTAGGTGGGGGTGGTAGTGGAGACCTTAACTTTCTTGCTCCACCAGGAGGAGGTGCAAGA

AATGTTGTTGCCTTATGTTTGCTAGAGACTCCTGCAGACAAGCCAGCAGCTGAAAGCATG

CCACCTCCACTAGCAGGTTTGTTTTTCACATTTATTCGAATGGTTTCGCCTTCCTTCAAC

CGATGATTGACAGCGGGATGGATCTCGATGTGGCTCTCATCAGTGTCGACGCTGGTTTCC

TTCTCGTGCTCCTTCCTCAGGT

||

TYPE: EST

STATUS: New

CONT_NAME: Santos E

CITATION: Identification of differentially expressed genes in response to Mycosphaerella fijiensis in the resistant Musa accession Calcutta 4 using suppression subtractive hybridization.

LIBRARY: SSH Calcutta M. fijiensis library

EST#: 1E6 BMCIBE

DNA_TYPE: cDNA

PUBLIC:

COMMENT: Raw sequences were clear up, from vector and cloning adaptors, with the program CLC Genomics Workbench (CLC bio, Denmark).

SEQUENCE:

CGGAGGGGCGGCTACGACGGGAGGTCGACGGTGCCAATGCTTTGGGACGCGGAGAGGAAG

GAAGTGGTGTGCAACGAGAGCTACGCGATCATCGAGTTCTTGAACTCCGCTGACTTCGGT

GGTGAGGTCCATGGTGGCTCAGGGTTGGACCTCTGCCCGTCGGAGCTCAAAAACGAGATC

GACGACTGGAATCGGGTGATCTATCCCAACGTTAACAATGGGGTTTATAGATGCGGGTTT

GCGCAGAGCCAAGAAGCATATGACACTGCAGTCAATGGATTGTTCAACACATTGGATATG

ATAGAATCTCATCTGTCTACCTCTCGCTACTTGTGTGGGGATGCACTGACTTTAGCTGAT

GTCTGCCTATTTACTACTTTGATCCGTTTCGACCTTGTCTACAACATCCTCTTCAAGTGC

ACAAAGAAGAAGCTACTTGAGT

||

TYPE: EST

STATUS: New

CONT_NAME: Santos E

CITATION: Identification of differentially expressed genes in response to Mycosphaerella fijiensis in the resistant Musa accession Calcutta 4 using suppression subtractive hybridization.

LIBRARY: SSH Calcutta M. fijiensis library

EST#: 1E7 BMCIBE

DNA_TYPE: cDNA

PUBLIC:

COMMENT: Raw sequences were clear up, from vector and cloning adaptors, with the program CLC Genomics Workbench (CLC bio, Denmark).

SEQUENCE:

ACTGTTCCTTCCAATTATCTCACTTGAAGCCACATGAACTTGTTGTTACAGCAGAGGACA

AAGAGGCAACAGAAATCTTCACGGGCAATCCTAACCCTGGAATGTCTGAATGATAGTGTT

GTGCCATGGGTCGGAAAGGTGCTACAGCAGGTTCTCGAATGGCCCTTTGCCAGTCACATT

GTGCTGGATGATGAACCCCAGGAAGGCCAACATCGCCAGTCTCCCGTTTGCGAGCTCCTT

CTCCTTGGCCTCCAGCG

||

TYPE: EST

STATUS: New

CONT_NAME: Santos E

CITATION: Identification of differentially expressed genes in response to Mycosphaerella fijiensis in the resistant Musa accession Calcutta 4 using suppression subtractive hybridization.

LIBRARY: SSH Calcutta M. fijiensis library

EST#: 1E8 BMCIBE

DNA_TYPE: cDNA

PUBLIC:

COMMENT: Raw sequences were clear up, from vector and cloning adaptors, with the program CLC Genomics Workbench (CLC bio, Denmark).

SEQUENCE:

ACGATTTGCTATTATTGTGCTCCCACAAACATCATCAAAGTAATATATCGAAAAGAAACG

TCTTGTCCGACAAATGCCACAGAAAAGGTTTTCCGTGAACGAGAAATATACGTAGCAACG

GAAGATGAACAAAGCCCGAGACAGCAGACCACAGGCGTAGCAGTTTTACGTTGAGAACTA

GATGACAGTTCCACTAGGGATCAGTGCATCTTTGATGACGGTGACGATGCCACTCTTGAT

GAAGTATCCGTCCGTCTCTCTTGCCGCTTCTTGCACGTTATCACAGTTGATGATCTTGAC

ATTCTCCCCAATGCGAGCATTCTTGTCGATGATGGCTCTTTTGACGTGAGAATTTCTTCC

GATGCCCATGGGAACACTGCCTTTTGCAGCTAACAGCCTCCTATCAGCATCAGTCTCATA

ATAATCTGCTCCCATCAGTAGAGTGTCTTCGAGGACTGCACCTTCTGATATACAAGAACG

AAGACCAATTACAGAATGGTGGATCTTGCAGTTCTTGATCACACATCCCTCGCCAATCAC

GCTATCAGTTACATCAGCATCCAGCATCTTGGAAGGAGGTAAATATCGGGGTTGTGTATA

AATTGGAGATGT

||

TYPE: EST

STATUS: New

CONT_NAME: Santos E

CITATION: Identification of differentially expressed genes in response to Mycosphaerella fijiensis in the resistant Musa accession Calcutta 4 using suppression subtractive hybridization.

LIBRARY: SSH Calcutta M. fijiensis library

EST#: 1F11 BMCIBE

DNA_TYPE: cDNA

PUBLIC:

COMMENT: Raw sequences were clear up, from vector and cloning adaptors, with the program CLC Genomics Workbench (CLC bio, Denmark).

SEQUENCE:

GGGAAAGGTCCCTAAGAACGACAGTTCCATCTCTTCCACCTTTTTGCCTCTTCTCTTCGA

GCATCGTGGCTTCATGCAGCATGTCGGCGGCATCGCTCTGCATGTCTAGCTTGGCCAGAG

CAACTGCTTGCATGTAGAATGCTGTCGGCCAATCTGGATAGACACATTGTGCTTGCATTG

CATCCCTCAGTGCAGCATCAGGTTGATCGCACAGGAGATGACACAGGCTTCGTCTTGCAT

ATATCGTAGGTGAGACCATAGTTCCAACATCTAAGAACTGAGAGTAACACTCAATAGCCG

TCTTAAAATCTTTATCACGAAAGGCAAAGTCCCCCCTCTTTCTGGCATCCAGTATATCCC

TCATCTGTTGCGTCCACTCTTGGAATGATAACTCATTGGTTCCTTCATCATCTCTGTAGT

GTGTCATTACTAAAATCTGATGGATGGCAGTGAGGTCCATTCTGGAGCAAGCTTCTCCCA

TAGGGGAAAGTGGGTGCGGTGGTGTGGCAGGTGCTTCCTCAGGCTTTTGTATTCCCAGCA

TAACATAAGACGGCT

||

TYPE: EST

STATUS: New

CONT_NAME: Santos E

CITATION: Identification of differentially expressed genes in response to Mycosphaerella fijiensis in the resistant Musa accession Calcutta 4 using suppression subtractive hybridization.

LIBRARY: SSH Calcutta M. fijiensis library

EST#: 1G5 BMCIBE

DNA_TYPE: cDNA

PUBLIC:

COMMENT: Raw sequences were clear up, from vector and cloning adaptors, with the program CLC Genomics Workbench (CLC bio, Denmark).

SEQUENCE:

AGGTACTTCCCGTCGCTGGACGACATCTGGAACGAGGACAGCGTGGAATGGNCAAGATTTTG

TGCACCATTAAGGTACAGANAACCACTTTCTTGGATTTTAGTTACCACTCGATCTAAAGA

TATTGCAAACGTGGTTGGGGATCCGTTCCCTCTTNNTGGTCTGGATGACACTGGCTATTG

GGAATTTTTCGGGCGGTGTACATTTGGTTCCGAATACGCTGGTGAATGTCCCCGGCTAGA

AATCGTAGGAAACCACATCGCTGGCTGGTTCAAGGGGTTGCCACTTGCGNCAAGGACGGT

AGGCGGGTTGTTTAAGACGCANATGAATGAGGAGCACTGGAGAATCATCGCATGGAGTGA

AATATGGCAACTACCGCAAAACCAAGAGGGTGTCCTGCCANTCCTCCNNCTGAGCTATCA

TTGTCTTCCCTCACACCTTAAGCGGTGTTTTGGTTTTAGTTCTATGTTCCCAAAAAATCA

TCTGNTTAAAAATCGGGACTTGAGACGGCATTGGATGGNACAAGGCTGCCCTGTGGTTCA

CGACGACTTGACGNTGGAGGATGCNNTAAGCCGCTACTTCTATGAAG

||

TYPE: EST

STATUS: New

CONT_NAME: Santos E

CITATION: Identification of differentially expressed genes in response to Mycosphaerella fijiensis in the resistant Musa accession Calcutta 4 using suppression subtractive hybridization.

LIBRARY: SSH Calcutta M. fijiensis library

EST#: 1G10 BMCIBE

DNA_TYPE: cDNA

PUBLIC:

COMMENT: Raw sequences were clear up, from vector and cloning adaptors, with the program CLC Genomics Workbench (CLC bio, Denmark).

SEQUENCE:

ACTCCATTTAGTCTCTCCTATTTTTGACATCACTTATATTTACTAGGTGTGGATTCTTTA

AGAAAAGATACTTGCATAATGTCCAGAATGACAGATAATGCTATAAATCAGAAGCACTAA

AGTGTTGTTTAATCTGCATCGAGCTTCTTGACATGTTTTTTAAACTCTCAAATTATCAGT

GAGTTAGCAACTGTTAGCTAGTAATTACCATCACAACAAGACCACAAACCTTTCGTATTC

ACTCAGTG

||

TYPE: EST

STATUS: New

CONT_NAME: Santos E

CITATION: Identification of differentially expressed genes in response to Mycosphaerella fijiensis in the resistant Musa accession Calcutta 4 using suppression subtractive hybridization.

LIBRARY: SSH Calcutta M. fijiensis library

EST#: 2A2 BMCIBE

DNA_TYPE: cDNA

PUBLIC:

COMMENT: Raw sequences were clear up, from vector and cloning adaptors, with the program CLC Genomics Workbench (CLC bio, Denmark).

SEQUENCE:

ACCAACCACAGCCACAGTGCCAATCAAAATGGCAGGACCCAATGATTTAAGTTTAAAGCA

GTCCTCCATCCCTAAACCATTTTGCATGACATCAACCCATTCGGGTCTCTCAAGCCCGAT

TTCAGTTGCATCTCACACAGTGGGTAGCTCAGGGAATGGTTCAAGCACCAATGATGATAC

AACAGGGCCTAAAGCTAGTAATCCACTGGTCCCAACAAACCGGAAAAATGTCACCAAAAC

TTTGTCTGCTGCTTTAGGCTCAGAAACTGTGGAAACTAGCACTCCTAAAGCTGT

||

TYPE: EST

STATUS: New

CONT_NAME: Santos E

CITATION: Identification of differentially expressed genes in response to Mycosphaerella fijiensis in the resistant Musa accession Calcutta 4 using suppression subtractive hybridization.

LIBRARY: SSH Calcutta M. fijiensis library

EST#: 2A4 BMCIBE

DNA_TYPE: cDNA

PUBLIC:

COMMENT: Raw sequences were clear up, from vector and cloning adaptors, with the program CLC Genomics Workbench (CLC bio, Denmark).

SEQUENCE:

GTATTCTGTGGAGCAAATATTAGAAGGTAGTGTTGAATGCGCCAAGAGGCTTGGAACATT

CGAAGCATGAATCTAATGTTGGTTCAACATGTCGCATGAAGAGCAAATAGAACATCTTCA

CATGCATGCTTGATGTCCCATACTGTATTTGTTGGTCATGAATCCATGAACACAAGGCTT

GTTGAAAAAAAAAAAAAAAAAAAAAAAAAAAAAAAGCTTG

||

TYPE: EST

STATUS: New

CONT_NAME: Santos E

CITATION: Identification of differentially expressed genes in response to Mycosphaerella fijiensis in the resistant Musa accession Calcutta 4 using suppression subtractive hybridization.

LIBRARY: SSH Calcutta M. fijiensis library

EST#: 2A6 BMCIBE

DNA_TYPE: cDNA

PUBLIC:

COMMENT: Raw sequences were clear up, from vector and cloning adaptors, with the program CLC Genomics Workbench (CLC bio, Denmark).

SEQUENCE:

ACAATACCTTATCAATGATACCATATTCAACTGCTTCACTAGGACTGAAATATTTCGGAC

GTCTGATATCTTCCTCGATTTGCTCACGAGATTTTCCTATATGCTTCGAGTATAAGCCAA

CCAATTCAGCCTTCACATTTCTTATTTCTTTTCTTGCAAGTTCAACATCTGTAGCTTGAC

CTTGAAATCTTGCAATAGGCTGCTTTATCATTATTGTTGATGATGGTAGAGCAGAACGGT

TTCCTTTAGAACCAGCAGCCAAGAGTAGTGCAGCTTCTCCCCAAGCATTGCCAACACACA

GTGTGAAGATTGGTGGTTTGACATAGCTCATGACATCGTAAATTGCAAAAGCCTCTGTCT

CATAGCCCAATTTCTCCCCNCCCTTTGTTGTTCCAGTGGAATTTATGTATAGGTAAATCG

GCTTTTCTACATCTTCATACTGAAGGTAAAGAAATTCTGCCATCATCAACTCTGTCACTG

ATGGAACAAGAGACA

||

TYPE: EST

STATUS: New

CONT_NAME: Santos E

CITATION: Identification of differentially expressed genes in response to Mycosphaerella fijiensis in the resistant Musa accession Calcutta 4 using suppression subtractive hybridization.

LIBRARY: SSH Calcutta M. fijiensis library

EST#: 2A9 BMCIBE

DNA_TYPE: cDNA

PUBLIC:

COMMENT: Raw sequences were clear up, from vector and cloning adaptors, with the program CLC Genomics Workbench (CLC bio, Denmark).

SEQUENCE:

AAGAATAGGATACTACATCGGTTCCAGCCAAAAAAA

AATTCCAAAAAAAAAAAAAAATTGACTTACCATAAAGAATTTAAAATCAAACCAAGGTTT

TTATCCAAAAGTAAAAAAAACCA

||

TYPE: EST

STATUS: New

CONT_NAME: Santos E

CITATION: Identification of differentially expressed genes in response to Mycosphaerella fijiensis in the resistant Musa accession Calcutta 4 using suppression subtractive hybridization.

LIBRARY: SSH Calcutta M. fijiensis library

EST#: 2A10 BMCIBE

DNA_TYPE: cDNA

PUBLIC:

COMMENT: Raw sequences were clear up, from vector and cloning adaptors, with the program CLC Genomics Workbench (CLC bio, Denmark).

SEQUENCE:

TAAAAGGAAATCTTGAAGCCTTTTCATGCAAGCAAACTGGGACAGATAGAAAACTCTCTT

TCAGCTTAGAACAAGAGATTCTTGATTATCTCGAACAATCTTCTGATAGTGACTCAGCGC

CTATGCAGTATTTGACTAGTAGATCAAGTCGAAAGACACTGATCTATCTTGTTCTTACAC

TAAGCCAA

||

TYPE: EST

STATUS: New

CONT_NAME: Santos E

CITATION: Identification of differentially expressed genes in response to Mycosphaerella fijiensis in the resistant Musa accession Calcutta 4 using suppression subtractive hybridization.

LIBRARY: SSH Calcutta M. fijiensis library

EST#: 2A11 BMCIBE

DNA_TYPE: cDNA

PUBLIC:

COMMENT: Raw sequences were clear up, from vector and cloning adaptors, with the program CLC Genomics Workbench (CLC bio, Denmark).

SEQUENCE:

ACGAGCTCTCGCTAAGGGACATGGTGGAGGCGCCGAGGGTGGCGAAGACGGTGCAGGAGA

TGGTAGAGAAGAGGAGGACAGAATCGAAGGATCGAAGTAAAGAGAAGAGAAGGCTGTTGA

GGAAGGAGAGCATCGAGACCGGGGTGTTCCTTAAGATGTTCGTGCCGATTTCCATAAGAG

GAGGGAGAAGGAAGAGCTTTGGGGGGTCTAATACTTGCTCAAAGGTTTCACCGAGGCCCG

TCTTGGCGGAAGCAGAGAAGGGTGGGCTTGAGGTCACGGAGGGAGAGTGGTGGGAGAAGG

AGTTGGGTGGCAGAGGAAGCAGCAGCAGCAGCAGTAGCAGCTCCAAAAGCAGCAGCAGTA

GCAGCTCCAAAAGTAGCATCGGAAGCAGCACAAGTAGAAGCGGTAGCAGGAAGATGAACG

GCTGCTACGCTTTCTTCCTCACAAATAAAAGCAGCTCCAAGGAAATTTAGGGATGCCAAG

TATTTTGTTTTGCTGATCACTGGAAGAAGAGAATGCAGCATGACATGT

||

TYPE: EST

STATUS: New

CONT_NAME: Santos E

CITATION: Identification of differentially expressed genes in response to Mycosphaerella fijiensis in the resistant Musa accession Calcutta 4 using suppression subtractive hybridization.

LIBRARY: SSH Calcutta M. fijiensis library

EST#: 2B1 BMCIBE

DNA_TYPE: cDNA

PUBLIC:

COMMENT: Raw sequences were clear up, from vector and cloning adaptors, with the program CLC Genomics Workbench (CLC bio, Denmark).

SEQUENCE:

CCATCGACAACCTCTTCGCGCACCTTGCTGACCCCGG

CCATGCCACAGTTTTCTCTGCTTTCACCCCCAACTGAGCAAGGATTGGAGAAGCACACGA

TGCAGTCATGATAGATGTTGTAATCCTCGTGTGTTCTTGAGCTGTGCTTGTAACGCTTTT

TACCTTTCTGCTCATGTAATTATAAGCAGCAATGGACGGTTTACCAGTTGCTTCAAAAAA

AAAAAAAAAAAAAAAAAAAAGCTTGT

||

TYPE: EST

STATUS: New

CONT_NAME: Santos E

CITATION: Identification of differentially expressed genes in response to Mycosphaerella fijiensis in the resistant Musa accession Calcutta 4 using suppression subtractive hybridization.

LIBRARY: SSH Calcutta M. fijiensis library

EST#: 2B6 BMCIBE

DNA_TYPE: cDNA

PUBLIC:

COMMENT: Raw sequences were clear up, from vector and cloning adaptors, with the program CLC Genomics Workbench (CLC bio, Denmark).

SEQUENCE:

ACCTTCTTTGGAGCATAATAATGAGGTCAGGGGAGAGAGTCTGGACTTGATAAAGTATAT

TGATGCTCATTTTGAAGGGCCTGCACTGAAACCTGATGATCCTGCAAAGCAGCAGTTTGC

AGAAGAGTTATTGTCATACAGTGATTCCTTCAATATGGTTATGTTTAAAGCAACGGCCGC

TAAGGGGGATGTCAGTGGTGAACTTGATGCTGCCTATGAAAAAATAGAGGATGCTCTATC

AAAGTTCAGTGATGGGCCTTTCTTCCTTGGGCAATTCAGCCTGGTGGACATTGCATATGC

TCCATTTGTTGAGAGGTTCCAGACTCTCTTGCTGGATGTGAAGAACTATGACATCCTCAA

GGGCAGACCCAAACTGGCATTGTGGATTGAGGAGCTGAACAAGATCGAGGCTTACGCGCA

AACCCGAATTGATCCTCAAGAGCTGCTGTCCGCAACCAAGAAACGGTTTGGGTTGGCATG

AAGAATATGTCTTCCAGTTTCAGATCAAGCAGTAATAAAACCATCAGCAGCCTTTGTAAT

AAAAATGGGACTTTCTTGTGTTAATGCTGTGTTTTCCAATGTGTGATTCCATGTATTCTT

CAAACATTTCAAAGCAATCAAGATTGNGCTTATGTGCTAAAAAAAAAAAAAAAAAAAAAA

AAAAAAAAAGCTTGT

||

TYPE: EST

STATUS: New

CONT_NAME: Santos E

CITATION: Identification of differentially expressed genes in response to Mycosphaerella fijiensis in the resistant Musa accession Calcutta 4 using suppression subtractive hybridization.

LIBRARY: SSH Calcutta M. fijiensis library

EST#: 2B8 BMCIBE

DNA_TYPE: cDNA

PUBLIC:

COMMENT: Raw sequences were clear up, from vector and cloning adaptors, with the program CLC Genomics Workbench (CLC bio, Denmark).

SEQUENCE:

GGGTCTCCACTCTCTACGCGCCTCTCATTCGTGACGGCCGTATGGAGAAGTTCTACTGGG

CGCCGACCAGAGATGACCGCATCGGCGTCTGCACGGGCATCTTCAGGACCGACAACGTCC

CCATGGAGGACATCGTCAAGCTCGTAGACTCCTTCCCGGGGCAGTCCATCGACTTCTTTG

GTGCTCTTCGGGCCAGGGTCTACGATGACGAAGTGAGGAAGTGGGTGGGAGACATTGGAG

TGGACAAGGTTGGAAAGAAGCTGGTCAACTCGCTCGAAGGGCCACCAACCTTTGAGCAGC

CCAAGATGAGCTTGGATACGCTGATGGAGTATGGCAACATGCTGGTGAAGGAACAGGAGA

ACGTGAAGAGGGTGCAGCTGGCCGACAAGT

||

TYPE: EST

STATUS: New

CONT_NAME: Santos E

CITATION: Identification of differentially expressed genes in response to Mycosphaerella fijiensis in the resistant Musa accession Calcutta 4 using suppression subtractive hybridization.

LIBRARY: SSH Calcutta M. fijiensis library

EST#: 2B9 BMCIBE

DNA_TYPE: cDNA

PUBLIC:

COMMENT: Raw sequences were clear up, from vector and cloning adaptors, with the program CLC Genomics Workbench (CLC bio, Denmark).

SEQUENCE:

ACACGTCTGACATAAACATTGTATCAAATGCAAGCTGCACAACCAACTGTCTTGCTCCTC

TAGCCAAAGTCATCCATGACAGATTTGGAATAGTGGAGGGTTTGATGACTACAGTTCATT

CTATCACAGCCACTCAAAAGACTGTTGATGGACCATTTAGCAAGGACTGGAGAGGCGGAC

GTGCTGCAAGCTTTAACATCATTCCTAGCAGCACTGGTGCTGCCAAGGCTGTTGGAAAGG

TCCTTCCTTCTTTGAATGGAAAGTTAACCGGTATGTCTTTCCGTGTTCCGACTGTTGATG

TGTCTGTTGTAGATCTTACTGTCAGGCTTGAAAAGGCTGCCACCTATGATGAGATCAAGG

CTGCCATTAAGGAGGAATCCGAGGGAAAGCTTAAGGGCATTCTAGGATATGTGGAGGAGG

ACTTGGTCTCCACTGACTTTGTAGGAGACAGCAGGTCAAGCATCTTTGATGCCAAGGCAG

GAATTGCCTTGAATCCTAACTTTGCCAAGCTTGTGTCTTGGTATGATAACGAGTGGGGTT

ACAGCTCACGTGTAATTGACCTGATCCGCCACATTCACAAGACCAAAGTAAATTCGATTG

CTTCCCCGACTTGCTGTTCTCCGGCCGTTCTTCGGACCATTGCTTTCTCTTTATTTTCAT

CACGAATAAATGTTTTGATGCGACATCTTTTTCATAATTGCCTCCCAGACTCTAGCCTGG

GCATTACATACATGCCCAGTTTTTGTTTTAGATGTTAATTATTCAGCTGAATGTGANGCT

GAAACCGTGT

||

TYPE: EST

STATUS: New

CONT_NAME: Santos E

CITATION: Identification of differentially expressed genes in response to Mycosphaerella fijiensis in the resistant Musa accession Calcutta 4 using suppression subtractive hybridization.

LIBRARY: SSH Calcutta M. fijiensis library

EST#: 2B10 BMCIBE

DNA_TYPE: cDNA

PUBLIC:

COMMENT: Raw sequences were clear up, from vector and cloning adaptors, with the program CLC Genomics Workbench (CLC bio, Denmark).

SEQUENCE:

GCAGACCTTCATTTTGATTTGGGTTACTTTCCGCACAGATTGGAACAAAGAGGTGGAACA

AGCCATGAGAAGACTAGACAAATGGGAGGACAACAAGCAACCTCTGCTGAGCAATCTTGA

CTAGGAAACAGATGACAGAATCAGCCTTCCTAGTTATGGAACATCTCCATTGACATCACA

ATTTACGAGGTGTCATCCATTACCTATGACAACATTACCAGCAGCAAGCTAGTATCTGAA

GACAGAGAGACCCTCAAAGAATGAAAGGAAGAAAGCAAGAAAGTAGATGGGTTTCCTCAA

GTTTCTCCTAGAAATGGATCTATCAGTGTGTTAGCAGTTATGTCCTAATCTTGGGCATTA

CAAGCTAAACAAGT

||

TYPE: EST

STATUS: New

CONT_NAME: Santos E

CITATION: Identification of differentially expressed genes in response to Mycosphaerella fijiensis in the resistant Musa accession Calcutta 4 using suppression subtractive hybridization.

LIBRARY: SSH Calcutta M. fijiensis library

EST#: 2B11 BMCIBE

DNA_TYPE: cDNA

PUBLIC:

COMMENT: Raw sequences were clear up, from vector and cloning adaptors, with the program CLC Genomics Workbench (CLC bio, Denmark).

SEQUENCE:

ACTCCGGCTGCCAGATTGGTAACACAGTCTCGCAACCATTGTCGTCGGAGGTCACTACCT

TTCCCCGGTGTAATGGATCCATCTTCAAGATTAATCCCATCGCTATCCTGTGCAGGTTCT

TCTTTATAATTGACGTCGTTAGTCAAATCATACAACCGTGAAGCACATAATTTAACCGCT

TCTATCTCTTTGAACACTGCCACCTTCCCACTTCCAAACTGTCTCTTAAATTCCCCATTC

ACAACAGCTTGCAAGTCTCCGAACGCCTCAGACCAGAGGAACCCATCGGAAGCATGCACG

TCTATAGATGGCTTTGAGCAGCCTGACATTGTCGCCGAAAAGATGCCAAAAATAAATACA

GTCATAACTCTAAATCCGTACCTCGGCCG

||

TYPE: EST

STATUS: New

CONT_NAME: Santos E

CITATION: Identification of differentially expressed genes in response to Mycosphaerella fijiensis in the resistant Musa accession Calcutta 4 using suppression subtractive hybridization.

LIBRARY: SSH Calcutta M. fijiensis library

EST#: 2B12 BMCIBE

DNA_TYPE: cDNA

PUBLIC:

COMMENT: Raw sequences were clear up, from vector and cloning adaptors, with the program CLC Genomics Workbench (CLC bio, Denmark).

SEQUENCE:

ACCGGCTCGGCCCGAACTACCTGACGCTCCCGGTGAACGCGCCCAAGTGCGCTCACCACA

ACAATCACTACGATGGACTGATGAACGTCATGCACAGGGACGAGGAGGTCGATTACTTCC

CTTCAAGGCATGCTTCCCTCCGTCATGCAGAGAGATTCCCCATTCCAAATCGTATCGTCA

CTGGCAAGCGTGAGAAGAATGTGATTCCCAAGCAAAACGATTTCAAGCAACCCGGAGAGC

GTTACCGTTCCTGGGCACCTGATAGGCAAGAGCGTTTCGTCCGCCGTTGGGCCGAGCAAT

TAGCACACCCAAAGGTCAGCTATGAGCTCCGCAGCATCTGGATCTCGTTCCTGTCGAAGT

GCGACACATCGCTGGGACAGAAGGTGGCGAATCGCCTCAACATGAGAGCAAACATCTGAT

AAGGAGTATGCATGTAAAAGCCTGCAGTATATGCGT

||

TYPE: EST

STATUS: New

CONT_NAME: Santos E

CITATION: Identification of differentially expressed genes in response to Mycosphaerella fijiensis in the resistant Musa accession Calcutta 4 using suppression subtractive hybridization.

LIBRARY: SSH Calcutta M. fijiensis library

EST#: 2C1 BMCIBE

DNA_TYPE: cDNA

PUBLIC:

COMMENT: Raw sequences were clear up, from vector and cloning adaptors, with the program CLC Genomics Workbench (CLC bio, Denmark).

SEQUENCE:

TCTTGTATTCAAACTTCACATCTCTGTTATTANNATCAATTTCCAGTGCTTTCTTGATGT

CCCCTTCTGCCAAATCCAGATCAGCTAGCTGCATATAAGCTTGTGCCCTAT

||

TYPE: EST

STATUS: New

CONT_NAME: Santos E

CITATION: Identification of differentially expressed genes in response to Mycosphaerella fijiensis in the resistant Musa accession Calcutta 4 using suppression subtractive hybridization.

LIBRARY: SSH Calcutta M. fijiensis library

EST#: 2C5 BMCIBE

DNA_TYPE: cDNA

PUBLIC:

COMMENT: Raw sequences were clear up, from vector and cloning adaptors, with the program CLC Genomics Workbench (CLC bio, Denmark).

SEQUENCE:

CACTTGAGATGGGTCGCCTTGCTATACCCATCATCAATTGAAGATGTGAATTCAANGATT

ACTAGCTCCTATCAGTATAAGACACTCCAGAGAATCCTTGGATGAGTATGACA

||

TYPE: EST

STATUS: New

CONT_NAME: Santos E

CITATION: Identification of differentially expressed genes in response to Mycosphaerella fijiensis in the resistant Musa accession Calcutta 4 using suppression subtractive hybridization.

LIBRARY: SSH Calcutta M. fijiensis library

EST#: 2C7 BMCIBE

DNA_TYPE: cDNA

PUBLIC:

COMMENT: Raw sequences were clear up, from vector and cloning adaptors, with the program CLC Genomics Workbench (CLC bio, Denmark).

SEQUENCE:

ACTTGTCGTCCGGCACGCCAAACGCCACCGCCTGCGCGATGTCGGAATGCTCCAGCAGCA

CCGCGTCCACCTCTATCGGAGAGATCTTCTCGCCTCCGCGGTTGATGAGCTCCTTGATGC

GACCGACGAGGTGGAGGTAGCCATCGCTGTCCAGGAAGCCGACGTCGCCGGTGTGGAACC

ATCCGAAGGCAAAGGCCGCCTTGTTCGCCTCCGGATTGTTCTTGTAGCCCCGGGTCACGT

TGGGTCCGCG

||

TYPE: EST

STATUS: New

CONT_NAME: Santos E

CITATION: Identification of differentially expressed genes in response to Mycosphaerella fijiensis in the resistant Musa accession Calcutta 4 using suppression subtractive hybridization.

LIBRARY: SSH Calcutta M. fijiensis library

EST#: 2C8 BMCIBE

DNA_TYPE: cDNA

PUBLIC:

COMMENT: Raw sequences were clear up, from vector and cloning adaptors, with the program CLC Genomics Workbench (CLC bio, Denmark).

SEQUENCE:

AGTAATTAGGAAGAGTAGTAGTGGAGAAAGGTTCA

TCTGTTCAGCCTTTTGTGATCTACGAGGATGTTTTCCCTCTTGATGCATCAAGAAGAATC

TAACATCCATCTATTAACATTCTTGCAAGCAAACCTGGAGATTCTTCTCGGCAAATGTTC

TTGTTCATTACAATGAAGATCATCAAGTTCTAATGCTCCAAGAATATGCAACCCTGCAGA

TCTGATATTTGTAGGTAAGCCAATAAGATGTTCTGATCATGGAAAAATTACTACTCCTGT

ATTTGCAGGATTGAAACAATTTCTATATGGAATGCTTGACTAATTCGTGGAGTTCAGCTG

GCAAAACAAGATCATCAAACCATGT

||

TYPE: EST

STATUS: New

CONT_NAME: Santos E

CITATION: Identification of differentially expressed genes in response to Mycosphaerella fijiensis in the resistant Musa accession Calcutta 4 using suppression subtractive hybridization.

LIBRARY: SSH Calcutta M. fijiensis library

EST#: 2C9 BMCIBE

DNA_TYPE: cDNA

PUBLIC:

COMMENT: Raw sequences were clear up, from vector and cloning adaptors, with the program CLC Genomics Workbench (CLC bio, Denmark).

SEQUENCE:

ACCATGCAACAGCTACC

CGGCAATGACCGACTCAGATCGAAAATACATTACGTAGACATTAACCTGACTCCAGTGTG

ATGACAAGTCTCTCATACGTGGAAGACGAGATACCCCATGTAGAGAAGAAGACCTGCTAC

CGTCAAGGAGATGACGGTGACGCTCCTCGGCATCCTTCCACCATCGCTTCTCAGAAACAT

GGCCTCGTAGATAGGCCAGCAGTTGGCGGCGACGAAGCCCGACAGGAACAGCTGCGCGAA

CTGCTCGTCGAGGAACCCCTCCGTCGTCGCAGCCCTCGCGATCCCTACCACCAAGGAGCT

CAGGTTGACGGCGGCCACGGTTCCGAGTGCCACGAAGAACGGCGACTGCACTCCCAGGTC

GAGGACCCCTCTCTCGT

||

TYPE: EST

STATUS: New

CONT_NAME: Santos E

CITATION: Identification of differentially expressed genes in response to Mycosphaerella fijiensis in the resistant Musa accession Calcutta 4 using suppression subtractive hybridization.

LIBRARY: SSH Calcutta M. fijiensis library

EST#: 2C10 BMCIBE

DNA_TYPE: cDNA

PUBLIC:

COMMENT: Raw sequences were clear up, from vector and cloning adaptors, with the program CLC Genomics Workbench (CLC bio, Denmark).

SEQUENCE:

ACTCAACAGCATTTCTCTCGTGATAAAACAAATTTGGGATAGAGAAGAACACAAACAAAA

TATACATGCCCATTTCATGCATCAAAAGCCGAGGTAGTGTGTAGCTATTACTCTCAACAT

AAGAAAAAAAATATCCTAACATCTTATTAAATGGAAAAAAAATCCACTCCAGAAGCTTAC

GTTATGCTATTTACATTTTTAACAAAACCTTCCTTCTCCTCGTCTACCAAGGCATGGGGC

ACATCGGATTGTCTTTTCTTCTGGAGCGCTTCTGCTCATCGAGTA

||

TYPE: EST

STATUS: New

CONT_NAME: Santos E

CITATION: Identification of differentially expressed genes in response to Mycosphaerella fijiensis in the resistant Musa accession Calcutta 4 using suppression subtractive hybridization.

LIBRARY: SSH Calcutta M. fijiensis library

EST#:   2C11 BMCIBE

DNA_TYPE: cDNA

PUBLIC:

COMMENT: Raw sequences were clear up, from vector and cloning adaptors, with the program CLC Genomics Workbench (CLC bio, Denmark).

SEQUENCE:

ACAAGCTTTTTTTTTTTTTTTTTTTTTTTTTTTTACATGAAATAATAATAATAGATAAT

AATCTTTCTCATTTTAGTTTGTCTACACACATTAATTTGTCGAATCCAGCAAACTAAGAA

TATAATCACCCAACAACTTACTCATTTGGAGATTTCTTTGCCCCATGTAAGATGATATAT

ACAAGAAAATGAAGTTATTTTCTCTTGCTGATAATTTGTGATATGATCCTACCAAGTTCT

TCACCTGAGTCCTCCTGAACATGATGCCCTGCCATAGCGAGCTCCACCAGCTTGTGATTT

GAAGCTTTGCAAAATTCCTCGACACCATCATACCTTAGCCAACGATCTCTCATACCCCAG

CAAATTGTGGTGCGAGTTTTCCATGTCTCACTAGCTAGTGTTGTCCTCATGGCTTCAACA

TAAGCCTTCAGCTCCTTCTTCATGGCTCCACTTAATGCATTTAGAGCAAAACCAGAAGAA

CCAGAAGTGAGGTATGGCCTTCTGTAAACCATCGCATCTTCTTCTTTCATCATGTATGGT

CCACAACTGGTCAATGCCCTATCACTAGCACTGANAGGATCCTGGGAGATAATTAGACCC

AACAAAAAGTTGCTGAACAGAGCCNAGGATGAGGGCAGCTTCACATGATTCTCCATCA

||

TYPE: EST

STATUS: New

CONT_NAME: Santos E

CITATION: Identification of differentially expressed genes in response to Mycosphaerella fijiensis in the resistant Musa accession Calcutta 4 using suppression subtractive hybridization.

LIBRARY: SSH Calcutta M. fijiensis library

EST#:   2C12 BMCIBE

DNA_TYPE: cDNA

PUBLIC:

COMMENT: Raw sequences were clear up, from vector and cloning adaptors, with the program CLC Genomics Workbench (CLC bio, Denmark).

SEQUENCE:

ACATGTTGGATTCAACAGCCAAAGTCATAGGAGGCGAGTCCTTGTAGCTCCGGTCCATTA

AGTCTACTGTTGTTGTCAAAGCTCTGAAGAGGAAAGTTTGCAAATGGTCCATCAATTGGA

ATGGTTCCACAAAGATCATTGTTTGAAACATCCAAGACTTTGAGATTTGAAAGCAAAACA

AGCTCTCTGGGAATGGCTCCGGAAAGCCTGTTGTTGTCTAGCCGCAAGAATCTAAGTGAC

TTCAATTTGGCAAAGGACTTAGGAATCTCTCCTTGCAGTCGGTTCTCATACAAATCCATG

CTGATGAGGCTCTTCAAATTGCCCAACTCCGCAGGGATTTTGCCCTCGAAGTTGTTCCTG

TAGAGCTCCAGGTATTGCAGGTGCTCCAGGCGGCTAAGCTCGGAGGCGAGAGGGCCGGAG

ATGTTGGAATTTCCCAAATCTATGCGAATAACACGGTTCTGGGAGTCGCAGGTGACATGG

AACCAGGTGCAGGGGTTGACGAGGGTGGGATCCCAGCTCTGCAGCACGTTGGTGGGATCG

GTGAGCCGCGTCCTCCATGCGTGCAACGCCTCTCCCTCGGAGTTGCTGGAGGAGGCCGGC

GAGGCGACGAGGAGGAGGAGGAGGATGTAGGAGAGGCAGGGCAGCGCCACCGGAAGAAGA

GCCATCCGAGAAAGAGAGCAGGAATGGAGGAGGGCGGCAGGT

||

TYPE: EST

STATUS: New

CONT_NAME: Santos E

CITATION: Identification of differentially expressed genes in response to Mycosphaerella fijiensis in the resistant Musa accession Calcutta 4 using suppression subtractive hybridization.

LIBRARY: SSH Calcutta M. fijiensis library

EST#: 2D1 BMCIBE

DNA_TYPE: cDNA

PUBLIC:

COMMENT: Raw sequences were clear up, from vector and cloning adaptors, with the program CLC Genomics Workbench (CLC bio, Denmark).

SEQUENCE:

AGGTCCAAATTGAGGGAAACAGTGTGGGATTCCTCC

ACTGATAGGTTTCTTTCCATTGAACACAGCATCTGGCCGAACAAAGAGAAGATCCTTGCC

ATTCGAAACCTTCCAAGATGTAATGCAGCCTCCGAACAGATAAATCTCAGCCTCACTGCC

ATGAGGGGAAAGCAGGGAGACCTTGGGAAGCCCCCATTCCCCTCCGAAATCTTGATCCCC

TGGGCATAAGTCTTTTGTTCTAAGCTCGCAAACGCGATTCCTGACGATCTGAAACGGAGC

CGTCGATCTGCGCTCCATGGGGTTAGGGCTCTTCCGAGGGGCAGGGAGGCCATCGCCACG

CTCGTCGCCATTGCTCCACCAA

||

TYPE: EST

STATUS: New

CONT_NAME: Santos E

CITATION: Identification of differentially expressed genes in response to Mycosphaerella fijiensis in the resistant Musa accession Calcutta 4 using suppression subtractive hybridization.

LIBRARY: SSH Calcutta M. fijiensis library

EST#: 2D3 BMCIBE

DNA_TYPE: cDNA

PUBLIC:

COMMENT: Raw sequences were clear up, from vector and cloning adaptors, with the program CLC Genomics Workbench (CLC bio, Denmark).

SEQUENCE:

ACAGCATCATCTGAAGGAACTGGAGGGTCATCGGACGTGCCATTAGAAACTAGTTCCGTT

CCTTCTTGCACATTCATCTCACTGAAGGTTCTTTTCCTTGTCAACGATTCCAACATCTCT

GTCTCCTTGACCTTCTCCCCCTTTTTCTCTATAGTTTCTTTTTCCTTCTCTGGCACTTCA

GTATCACTTGTAGAAGGCTCAACCGGTGGTGGCAGCTCACGACCATTTAACCGGCAATAT

ACTCTTTCAACGGTTTCGCTGATCTCCTTCCCCAAGCCATTATTGTCAACAATTAGCTCC

CAAACAGCTCTAGATGCCTTCTCAAGCACCGGAGTTTCTAGTTCTCGCCGCAGTGCATCA

AACAACTCTCTCTTTGTCTGTTTCTCTGCACCAGGAGTATTAAGGACCTTACTTTGCTCG

ACCATCGTGATTGTGTTATTCTTAAGCTCTTCATTGGCCTTCAACTGGTTAATAATCTTC

AATCTAATGGCATCGATTGTCCCATCATTCATTAACGATTCGAGT

||

TYPE: EST

STATUS: New

CONT_NAME: Santos E

CITATION: Identification of differentially expressed genes in response to Mycosphaerella fijiensis in the resistant Musa accession Calcutta 4 using suppression subtractive hybridization.

LIBRARY: SSH Calcutta M. fijiensis library

EST#:   2D5 BMCIBE

DNA_TYPE: cDNA

PUBLIC:

COMMENT: Raw sequences were clear up, from vector and cloning adaptors, with the program CLC Genomics Workbench (CLC bio, Denmark).

SEQUENCE:

ACGAAGGGGCTTGTCTGATGGTCTCTTTGGTTCCTGGATCAGGTCCAGAGCCTCGAGAAG

GGTTGGGCCCTATACTGAG

||

TYPE: EST

STATUS: New

CONT_NAME: Santos E

CITATION: Identification of differentially expressed genes in response to Mycosphaerella fijiensis in the resistant Musa accession Calcutta 4 using suppression subtractive hybridization.

LIBRARY: SSH Calcutta M. fijiensis library

EST#:   2D6 BMCIBE

DNA_TYPE: cDNA

PUBLIC:

COMMENT: Raw sequences were clear up, from vector and cloning adaptors, with the program CLC Genomics Workbench (CLC bio, Denmark).

SEQUENCE:

ACAAGCTTTTTTTTCTTTTTTTTTTTTTTTTTTTGCTAATTTGTTTCACCTCAATACCAT

AAACATATCCAGTATCGTATGCTTCCCTTATTCAAAAAAGAAATACACAGGCACAAAAAC

TCCTAAACATGCATAAAAGATAAAAAGGACGCGAGCCAAATCATAAACTCGTCGAGTCGC

TTAGCTTCTGCTGATACGAGGCTTTAATCAACTTCCTCAATCTTTGGCCCTGCACCACTG

CTGCCTCCGGTGCTGGGAATGTCCTCGTCCATACCACCGCCCATTCCACCGCCAGCGCCC

TGATACATCTTTGCGATGATCGGGTTGCAGATGCTCTCCAGCTCCTTCATCCTATCCTCG

AACTCGTCGGCCTCGGCCAGCTGGTTGCCGTCCAACCAGCTGATCGCCTGCTCCACTGCG

TCTTCGATCTTCTTTTTGTCCTCCGCCGGCAGCTTCGCAGCTATCTTCTCGTCCCTAATG

GTGTTCCTCATGTTGTAGGAATAATTCTCCAAGGCGTTCTTCGCCTCGATTTTCTTCTTG

TGCTCCTCGTCCTCCGCCTTGT

||

TYPE: EST

STATUS: New

CONT_NAME: Santos E

CITATION: Identification of differentially expressed genes in response to Mycosphaerella fijiensis in the resistant Musa accession Calcutta 4 using suppression subtractive hybridization.

LIBRARY: SSH Calcutta M. fijiensis library

EST#: 2D7 BMCIBE

DNA_TYPE: cDNA

PUBLIC:

COMMENT: Raw sequences were clear up, from vector and cloning adaptors, with the program CLC Genomics Workbench (CLC bio, Denmark).

SEQUENCE:

ACTCAGCAACTTGCTTTGGTGTGGCTTTCTCTGGGCACTCAGCGCCCGGGGTCACCATGC

TCGGCTTCAGGAGGATGCCTTCGAACATGACATTGTTCTCGGCCAGGTAGAAGAAGACCT

CAGCCCATACTTTCTGTGCAACCTCGAAGGTCCTCTCGATTCCGTGGTCTCCGTCAAGCA

GGATTTCTGGCTCCACAATCGGCACCAACCCATTGTCTTGTGCGATGGCAGCGTATCGAG

CCAAGCCCCAGGCAGCTTCCTTGACTGCAAGAGTCGATGGGCCATTTGGAATGCTGACCA

CTGT

||

TYPE: EST

STATUS: New

CONT_NAME: Santos E

CITATION: Identification of differentially expressed genes in response to Mycosphaerella fijiensis in the resistant Musa accession Calcutta-4 using suppression subtractive hybridization.

LIBRARY: SSH Calcutta M. fijiensis library

EST#: 2D10 BMCIBE

DNA_TYPE: cDNA

PUBLIC:

COMMENT: Raw sequences were clear up, from vector and cloning adaptors, with the program CLC Genomics Workbench (CLC bio, Denmark).

SEQUENCE:

ACGGTCCTGACAGAAGAATTTTCTTGCCGGAAGGCCTCCTGGACCGATCTGATATTCCAG

AGTATCTCAATGGAGAAGTTCCGGGAGATTATGGCTATGATCCTTTTGGGCTGAGCAAGA

AACCTGAGGATTTTGCCAAATACCAAGCTTATGAGCTCATCCATGCAAGGTGGGCAATGC

TTGGTGCAGCTGGCTTCATCATCCCAGAGGCCTTCAACAAATTTGGTGCAAACTGTGGCC

CTGAGGCTGTCTGGTTCAAAACTGGTGCTCTTCTCCTGGATGGGAACACGCTGAACTACT

TTGGGAAGAGCATTCCAATCAATCTTGTGGTTGCTGTCATTGCCGAGATTGTGCTTGTTG

GAGGTGCTGAATACTACAGAATCATCAATGGACTGGATTTGGAGGACAAGCTGCACCCTG

GAGGTCCATTTGATCCACTGGGGCTGGCGGATGATCCAGACCAATTTGCATTGCTCAAGG

TGAAGGAGATCAAGAACGGGCGACTCGCGATGTTTGCGATGCTGGGGTTCTTCCTGCAGG

CCTATGTCACTGGGGAAGGACCAGTGGAGAACCTCACCAAACATCTGAGTGACCCATTTG

GAAACAATTTGCTCACTGTCATCTCTGGAGCAGCTGAAAGAGCTCCAACCCTGTGATCTC

CCAGCCGTGTTTCTTCTCTTCCATGATCTGCATGCCATGTTGACCACTTAGATCATTCAT

CGCATTGTAAAATGTTATTTGATACTTCAGTAATTCTTGTTTCAGCGGANAAAAAAAAAA

AAAAAAAAAAAAAAANGCTTGTACCTGC

||

TYPE: EST

STATUS: New

CONT_NAME: Santos E

CITATION: Identification of differentially expressed genes in response to Mycosphaerella fijiensis in the resistant Musa accession Calcutta 4 using suppression subtractive hybridization.

LIBRARY: SSH Calcutta M. fijiensis library

EST#: 2D11 BMCIBE

DNA_TYPE: cDNA

PUBLIC:

COMMENT: Raw sequences were clear up, from vector and cloning adaptors, with the program CLC Genomics Workbench (CLC bio, Denmark).

SEQUENCE:

ACCTGAAGATGTATCAGGTTTTCGGACACTCTTCTGAGAACAAATTATGGGCTGCAGAGG

TTCTTTAGGTCCTCCTCTTTTGCAAAGGAGGTTGGAATGATCCTTGAAGAATAAGGATAG

AGATCTTTGTAGGAGTTCCGGATGGTGCCTTCCGCTACTCCGGTTGCCAGGGATATGTCT

TTGAGAGGCTTCTTGTCATCTGATAACTGTGTTATCATGTAAATGATAGCCGCTGCAATT

GATATAGGACTTCTCCTTATATCAAGCTCTTCTGACTTCTGGACTGCTTCCTGAGCAGCT

TTAACTGCTTGATTTGTCATACCAAGATGTGAACAAAAACGTCTCAGAAAATCTCCAGCA

TGAATTGTTCCCATCTCCATTGACTGTCCCATCTCAACTTCAAGCTGTTTTACAATGAAC

TCCTTAGCCCGACCAATTTCTTTCTTTGCAGCTCCATTGGCAACAGAGCAAATTTCCTTC

ACAGTTCGAGGCCTGTCTTCCTGTCGGCAAGCAATGTATAGACAAGCAGCTAAAATTGCA

TCTTGAT

||

TYPE: EST

STATUS: New

CONT_NAME: Santos E

CITATION: Identification of differentially expressed genes in response to Mycosphaerella fijiensis in the resistant Musa accession Calcutta 4 using suppression subtractive hybridization.

LIBRARY: SSH Calcutta M. fijiensis library

EST#: 2D12 BMCIBE

DNA_TYPE: cDNA

PUBLIC:

COMMENT: Raw sequences were clear up, from vector and cloning adaptors, with the program CLC Genomics Workbench (CLC bio, Denmark).

SEQUENCE:

ACATACTTCACAAAGCAAGCAAAGCCAGTGATGACATTAGTGGATGATGCAGCACTTCTC

AGACACTTCCAAGTTAGTCACCGCTACATAGTCACACCATGACATACTTACACATATGCA

CACCATGACAGGAAGTAACACATTTGTTCTGACAGAAAAAGAACTAAAACTACAGGAGGT

CAAGCTTCGTATGCCGGAAATCCCGATTGAGAAGGTGTAGTCATGTCTAAAAAGAGGTAT

GCCATGCCAGCTTGCCCTTCGAAGAGTGAATAAGGATGATCACCACTGTGCATCTTCCCT

TCGGCAATCAATTTATTAGCTCTATCTAGCAGAAAGCAAGTAAAGGCTTTTGCTTGATAT

A

||

TYPE: EST

STATUS: New

CONT_NAME: Santos E

CITATION: Identification of differentially expressed genes in response to Mycosphaerella fijiensis in the resistant Musa accession Calcutta 4 using suppression subtractive hybridization.

LIBRARY: SSH Calcutta M. fijiensis library

EST#: 2E1 BMCIBE

DNA_TYPE: cDNA

PUBLIC:

COMMENT: Raw sequences were clear up, from vector and cloning adaptors, with the program CLC Genomics Workbench (CLC bio, Denmark).

SEQUENCE:

AGAGAGAGCCCATCGAAGTTCGCAGGCAGCGGAAGA

GGGAAAAGGGCGTTATGGAGGGAGGAAAGAAGCAGGACGCCGGTGCCCCCGCCAATTCGT

CGTCCTCCTCCTCCACTTCTTCTTCCTCGCCCTCCGGCTTCTTCAGCAGTGTCTTCCCTC

CTCCTTCCACGGTGATGGCTAAGGATTCATCGCAGAGTGACTTGT

||

TYPE: EST

STATUS: New

CONT_NAME: Santos E

CITATION: Identification of differentially expressed genes in response to Mycosphaerella fijiensis in the resistant Musa accession Calcutta 4 using suppression subtractive hybridization.

LIBRARY: SSH Calcutta M. fijiensis library

EST#: 2E2 BMCIBE

DNA_TYPE: cDNA

PUBLIC:

COMMENT: Raw sequences were clear up, from vector and cloning adaptors, with the program CLC Genomics Workbench (CLC bio, Denmark).

SEQUENCE:

ATGAATGATGTCAACAGCCAGAAAGCTCTGGAAGAG

TGGCGGATGCGGAAGATGGAACGAGCAAGACAACGGGAAATTGAGAAAAATGGAACATTG

ACATCTTAGTTTGGTGGTTGCATTTGAGATCTAAGAAGAATGCTACTAAAATGTATACGT

ATGTGCATCAAAGCAGCGGCAGCTCTTTGAAGTTTATTTATATTTACTGTCTGTCTGTCT

GTCTGTATTGTTAATTTGAGTGAACACAAATCAAGCTGTTATTGTAAATCTAAAGATAAG

GGACTTTATATGTAGTATATGTTCAACAGGACTTTATATAATTGTGCTGTAATTTCTTTT

GACTGCTAAAAAAAAA

||

TYPE: EST

STATUS: New

CONT_NAME: Santos E

CITATION: Identification of differentially expressed genes in response to Mycosphaerella fijiensis in the resistant Musa accession Calcutta 4 using suppression subtractive hybridization.

LIBRARY: SSH Calcutta M. fijiensis library

EST#: 2E3 BMCIBE

DNA_TYPE: cDNA

PUBLIC:

COMMENT: Raw sequences were clear up, from vector and cloning adaptors, with the program CLC Genomics Workbench (CLC bio, Denmark).

SEQUENCE:

CAAGCTTTTTTTTTTTTTTTTTTTTTTTTTTTTATGATATTAATCCAGTCACAAATGCCT

TTCATATGGATAGGATGTTACAAGGGATATATCTGTGGCAACAAAGCATGTCACGATGAG

CTGCAGCACCCTCAGTGGAACTTGAAGCTGGTCAAGATGTTGTTGTGCACAGGGTCGGCC

AAGTGATCCAACAGGTTCTGGAACGGCCCAACTCCGGTCAACGCCGCCTGCGCAAAGTAA

CCCAGGATGGCCAGCATGGCCAGCCTCCCATTCTTCACCTCCTTCAGCTTCAGATCGTTC

ATCGACTTCTCGTCCTTCCCGAAGCCGAAAGGGTTGAAAAAAGGTCCTCCAGGGTAGATA

GGGTTTCCCGACCCCCCCAGCCACTTCTCGAAACCTAAAAAGT

||

TYPE: EST

STATUS: New

CONT_NAME: Santos E

CITATION: Identification of differentially expressed genes in response to Mycosphaerella fijiensis in the resistant Musa accession Calcutta 4 using suppression subtractive hybridization.

LIBRARY: SSH Calcutta M. fijiensis library

EST#: 2E4 BMCIBE

DNA_TYPE: cDNA

PUBLIC:

COMMENT: Raw sequences were clear up, from vector and cloning adaptors, with the program CLC Genomics Workbench (CLC bio, Denmark).

SEQUENCE:

TGTTCCCGGCCAAGGTACCTCAGCTGATCTTCTCCTCTGAGGGGCTTTGA

TCCCAAAGAGNTCTCCAAACGATGGAACCAGATAAAACACGCTCTGTTGCCCACCCTGCT

GACTTCAACTTTGCGATGAACTCATG

||

TYPE: EST

STATUS: New

CONT_NAME: Santos E

CITATION: Identification of differentially expressed genes in response to Mycosphaerella fijiensis in the resistant Musa accession Calcutta 4 using suppression subtractive hybridization.

LIBRARY: SSH Calcutta M. fijiensis library

EST#: 2E6 BMCIBE

DNA_TYPE: cDNA

PUBLIC:

COMMENT: Raw sequences were clear up, from vector and cloning adaptors, with the program CLC Genomics Workbench (CLC bio, Denmark).

SEQUENCE:

AAGATTTCGGACTTCGGCATGGCCAGAATATTTGGAGGGGATGAGACGGAAGTAAATACC

ATGAGAGTGGTGGGAACATATGGTTACATGTCGCCCGAATACGCCATGGACGGAATCTTC

TCGGTGAAATCTGACGTGTTCAGCTTCGGCGTGCTGGTGCTCGAGATCGTAAGCGGCAAA

AAGAACCGCGGCGTTTATTACTCCGGTCGCCACCTAAATCTTCTGGCATACGTAAGTAGA

AGAAATTATAATCTTTACATACGATTGATTCCCTTTATGGTGATGATGCCATCGATACTT

GCGATTGATCTTAGACGTGGAGTCTCTGGAAAGAAGACAGAGTGCTGGAAATGGTGGACG

AATCGATCGGGGAGTCCTTCCCCACGGATGAAGTCTTGAGGTGCATAAAGGTCGGGCTTT

TATGCGTTCAAGAGATGCCAGAAGACAGACCGACGATGTCTTCAGTGGCGTTGATGTTGG

GGAGCGACGGTGCTCTCCTGCCGCAACCTGCTCACCCAGGTTTTGTGTCCGCCAGGTGCC

CGATCGAAAATGGATTCATCGATAAGTAAGCAGGAGTCGATGTCCATAAACNAACGTATC

GGTCACAATGTTCGAAGGTCGGTTAGATCAACTAAAAACGT

||

TYPE: EST

STATUS: New

CONT_NAME: Santos E

CITATION: Identification of differentially expressed genes in response to Mycosphaerella fijiensis in the resistant Musa accession Calcutta 4 using suppression subtractive hybridization.

LIBRARY: SSH Calcutta M. fijiensis library

EST#: 2E7 BMCIBE

DNA_TYPE: cDNA

PUBLIC:

COMMENT: Raw sequences were clear up, from vector and cloning adaptors, with the program CLC Genomics Workbench (CLC bio, Denmark).

SEQUENCE:

GCCAAACCACAACATCACAGTCTACTAATACTGCAA

GACCCAAAATGACGCCAGACAACAGACAAGTTCCAAACTTAAAGATAACCAAAGCAGCAG

ATAACTACGAAGCCCAGCCCTCCGATCGCGGCAAGCGAACGACCGCCTTAGCTCCTCCAG

TACCTCGGCCG

||

TYPE: EST

STATUS: New

CONT_NAME: Santos E

CITATION: Identification of differentially expressed genes in response to Mycosphaerella fijiensis in the resistant Musa accession Calcutta 4 using suppression subtractive hybridization.

LIBRARY: SSH Calcutta M. fijiensis library

EST#: 2E8 BMCIBE

DNA_TYPE: cDNA

PUBLIC:

COMMENT: Raw sequences were clear up, from vector and cloning adaptors, with the program CLC Genomics Workbench (CLC bio, Denmark).

SEQUENCE:

ACCTTTGGAGAAAGTTAAGGAGATGAGTAACTTATCTTTAATGAGAACAGTGGAGAGTTG

GCTAAGGAAGGAGAGGGATGAAGACAGGAGGAGGAGACAGGAGAGAAAGGCAATTGGCCT

TGCAGAACATGAAAAGCAACTCGAGGGGACACGGGTGACAGGGGATCCAGCCCGGAGTGC

AGTCCGAGTAATGAACAATGGTTGGGCAGCCGGGAGACTACATTCAACTGTAGCAGGTCA

GGAGAATTTTTTTCTGAGTTCAAATAGAGTCCTTAGGCAATAACAGCTTCTGCATGTTCT

TGAGTTTGGTGAATCTCGTCTGTTGTGTTTACGAGAACTAAAGAAAATGTTTGGTTATAG

GTGCTCATATTCCAAAGATAATTATTGTTGT

||

TYPE: EST

STATUS: New

CONT_NAME: Santos E

CITATION: Identification of differentially expressed genes in response to Mycosphaerella fijiensis in the resistant Musa accession Calcutta 4 using suppression subtractive hybridization.

LIBRARY: SSH Calcutta M. fijiensis library

EST#: 2E9 BMCIBE

DNA_TYPE: cDNA

PUBLIC:

COMMENT: Raw sequences were clear up, from vector and cloning adaptors, with the program CLC Genomics Workbench (CLC bio, Denmark).

SEQUENCE:

ACAAGCTTTTTTTTTTTTTTTTTTTTTTTTTTTTCTTTTTTTTAAACGGAACAAAGCGC

CTTATTAATTTAGTATTCTGGGAAGGAGGACAACAAAATTATTTACCGGCGATGGCGCTC

GAACAAAAAAGAGGAAAAACAGCGGGCAACATACGGCCATTACAGCACGGGGCACAAGAA

AAGCATCAACCACAACACACACTCATCGAACCGTCCAAGCCAAGCTGCCTACGTTGTTGA

CCAGCTGCTAGCCACCGACGACGACGTGCTCTCACAGGAAACGGTTCAGGAATTCGTCAA

CGGGGGTGT

||

TYPE: EST

STATUS: New

CONT_NAME: Santos E

CITATION: Identification of differentially expressed genes in response to Mycosphaerella fijiensis in the resistant Musa accession Calcutta 4 using suppression subtractive hybridization.

LIBRARY: SSH Calcutta M. fijiensis library

EST#: 2E10 BMCIBE

DNA_TYPE: cDNA

PUBLIC:

COMMENT: Raw sequences were clear up, from vector and cloning adaptors, with the program CLC Genomics Workbench (CLC bio, Denmark).

SEQUENCE:

ACTCGTGGGCTTGCCCGGTGGCGTGGACGCTGTATGGGCTCGTTGCCTCACAGTTCGGCG

ACAACCAAACTATAATGGAGGGTGGTGAGTCGGTGGAGGAATACGTCAGGCGATTCTTCG

GCTTCCGGCATGACTTCCTGGGCGTCGTGGCTGTTGCAGTGGTCGGCTTCACCGTGCTCT

TTGCTTTCGTCTTCGCCTTCTCGATCAAGGTGTTCAACTTCCAGAGAAGATGAGCCACCA

CATTCTTCTTCCAAATCCACCGAGCAATGTCCATATCACGAGATATATATGGAATAATCC

ATCTTTCTTTTTGTGTATAGGATAATTATCCTCCCTAAACATGTATTCCTTTGTTGTCAA

TCCACAAATGT

||

TYPE: EST

STATUS: New

CONT_NAME: Santos E

CITATION: Identification of differentially expressed genes in response to Mycosphaerella fijiensis in the resistant Musa accession Calcutta 4 using suppression subtractive hybridization.

LIBRARY: SSH Calcutta M. fijiensis library

EST#: 2E11 BMCIBE

DNA_TYPE: cDNA

PUBLIC:

COMMENT: Raw sequences were clear up, from vector and cloning adaptors, with the program CLC Genomics Workbench (CLC bio, Denmark).

SEQUENCE:

GAGGAACTTCGGACAATTCCTGGGAGAACTGCACTAGAACTTCGTGCAGAGGCAAGTATA

TAGCCTTTTTCTTTTTCAGCAGTTCTGCATAGCTGAAATATGTTATACTTGAATTTTCCA

ATTTGCAGGTGGCTCCTATGACATCTAGTTTGAAAAATCAGAGGCTTGAACTGAATGAAA

GAGTCACCAAAATATCAGAACTTGGGATTCGAGTTTGATAATAAAGTATTTT

||

TYPE: EST

STATUS: New

CONT_NAME: Santos E

CITATION: Identification of differentially expressed genes in response to Mycosphaerella fijiensis in the resistant Musa accession Calcutta 4 using suppression subtractive hybridization.

LIBRARY: SSH Calcutta M. fijiensis library

EST#: 2F1 BMCIBE

DNA_TYPE: cDNA

PUBLIC:

COMMENT: Raw sequences were clear up, from vector and cloning adaptors, with the program CLC Genomics Workbench (CLC bio, Denmark).

SEQUENCE:

ACCCGCTTAGTATAATAAGTGGCGGCACAACTGCGAAGATTATGGTGGCAGCAAAAGATA

ACTTTCTTGGAAAATATATCTACAAAGACACCATTGCTCGAAATATTGCAGCTGTTATAT

ATAGAGATGAGAAGGAAATACAAAAGACAGCAGTTAAACAATACCGTGTTTTGCGATCTG

CTACATCATTTAGATATGGTTACAAACTTGTTGAAAATAACAATTTGAGGGCTGCAATTT

CTACTTCCAATGTCATTGAACTTCCAACACAGGAAGAGCTTAAAACCGTCCTTGACAAGG

TGAAAGACTTCTTCGGCAATGCTACCAGTGATGCTAAAGAGTCATTTGGAAAGCTTACAT

CCTTGGGTTCATTAACTGATGAGGAACCAGAATCACAATCCGAAGTGAAGAGCTGAAGGA

ATATGGACTTCTGACATCAAGATGGCCAAAGATCTCTGTGAGCTGATATCTTTTCTGATG

TTATCAAATCTTTACCTTCGTTGTTCGT

||

TYPE: EST

STATUS: New

CONT_NAME: Santos E

CITATION: Identification of differentially expressed genes in response to Mycosphaerella fijiensis in the resistant Musa accession Calcutta 4 using suppression subtractive hybridization.

LIBRARY: SSH Calcutta M. fijiensis library

EST#: 2F2 BMCIBE

DNA_TYPE: cDNA

PUBLIC:

COMMENT: Raw sequences were clear up, from vector and cloning adaptors, with the program CLC Genomics Workbench (CLC bio, Denmark).

SEQUENCE:

CACCGCCGAGGAGCAGGGGATTCTCGGTCCCAAAA

TTCACAATCCCATCGAGGAGACCTCCCACATCGAAGTCGAGGGAGGAGCTGCCGACGGTC

TGGTCGAGAGCTTGCTCGTAGGTCAAGGCCTTGGCGAGGCCACCAGCACCAAAAATCGGG

GAGAGGGCTGCAAGCTTGGGGAGCCTGAATTGGGGGAGCCGCCTAGGCTCGGGTCTCCTC

TCGAGGGAGGGAGTGGCCTTGTGGAGTGAGGCTACTGGTGCCAGGCCTGCTGCATTGAGG

ACTTGCATGGCATAATTATC

||

TYPE: EST

STATUS: New

CONT_NAME: Santos E

CITATION: Identification of differentially expressed genes in response to Mycosphaerella fijiensis in the resistant Musa accession Calcutta 4 using suppression subtractive hybridization.

LIBRARY: SSH Calcutta M. fijiensis library

EST#: 2F3 BMCIBE

DNA_TYPE: cDNA

PUBLIC:

COMMENT: Raw sequences were clear up, from vector and cloning adaptors, with the program CLC Genomics Workbench (CLC bio, Denmark).

SEQUENCE:

ACGTGAACTCTCCAGGAGGATCAGTTACAGCTGGCATGGCTGTATTTGACACAATGCGAC

ACATTAGGCCTGATGTTTCTACTGTTTGTGTTGGTCTGGCAGCCAGTATGGGTGCTTTCA

TTTTGAGCTCTGGCACAAAGGGGAAAAGATACAGCTTACCAAACTCCAGGATTATGATTC

ATCAACCTCTTGGAGGAGCTCAGGGTGGGCAAACCGATATAGACATCCAGGCAAATGAAA

TGCTTCATCACAAGGCCAATTTGAATGGATACCTGGCATACCACACTGGTCAAAGCTTTG

AAAGGATCAACCAAGACACTGATCGGGACTACTTCATGAGTGCCAAAGAAGCTAAAGAAT

ATGGTTTGATCGATGGTGTGATCATGAATCCTCTCAAAGCTCTGCAGCCACTACCAGCCT

CGCAAGAGTGATTGCCAATCAGCTAAATGCCTTGTTAGATGTTACTTCTGTGGCCATAAG

CGAGGAAGGATGATTCTACGAACAAATTGCCCAAGAGAGCAGTGTTTTTGTTATTTGTGT

TGCAAAGT

||

TYPE: EST

STATUS: New

CONT_NAME: Santos E

CITATION: Identification of differentially expressed genes in response to Mycosphaerella fijiensis in the resistant Musa accession Calcutta 4 using suppression subtractive hybridization.

LIBRARY: SSH Calcutta M. fijiensis library

EST#: 2F4 BMCIBE

DNA_TYPE: cDNA

PUBLIC:

COMMENT: Raw sequences were clear up, from vector and cloning adaptors, with the program CLC Genomics Workbench (CLC bio, Denmark).

SEQUENCE:

ACAGCAACACCCCAACCATCATGGACACATTCAAATGCAGATATCATGGCACTTGTGCTG

CCAGAACATTCGATGCTGCGATCGACTCCACCGTTGGTCATTTCAGCAAGCACCTCTTGA

ACTGGTCTGTCATAGTCTGCTGGATTCACAAACTCGGTCACACCAAACTTCTTAGCTTCC

TCAAATCTCTTAGGATTAATATCAACACCAATTATTCTTGATGCCCCTGAGGCCCTTGCC

CCTTCAGCTGCTGCAAGGCCTACAGCTCCAAGGCCAAAAACAGCCACAGTTGAACCCTTA

GGTGGTTTTGCAACATTCACAGCAGCACCATAACCTGTTGAAATGCCACAGCTGATGACA

CAAACTTTGTCGAGAGGAGCCAAAGGGTTGATCTTGGCAACACAGCCGACATGGACGACG

GTGT

||

TYPE: EST

STATUS: New

CONT_NAME: Santos E

CITATION: Identification of differentially expressed genes in response to Mycosphaerella fijiensis in the resistant Musa accession Calcutta 4 using suppression subtractive hybridization.

LIBRARY: SSH Calcutta M. fijiensis library

EST#: 2F7 BMCIBE

DNA_TYPE: cDNA

PUBLIC:

COMMENT: Raw sequences were clear up, from vector and cloning adaptors, with the program CLC Genomics Workbench (CLC bio, Denmark).

SEQUENCE:

ACAACAGGGAGACCTTATGTCTGCCACCGCCTGTCGAGCACCCAGTTCTGCACCCAGACA

CCAGATACAGTGCCTATGGACTGACAAAGACGACCAGGACACATACTAAGACAGCAAATC

ACTCCAAATAATATTAACGCAGAATTCTCCTCCCTTCATTTTTTCTTTTGGGCTGCTTTG

GTAACCTTGGCTCCTGTGGGATCCTTCTTCTCCACGCTTTTGATAACACCCACGGCAACC

GTCTGTCTCATGTCTCTAACAGCAAACCTACCCAGCGGCGAGT

||

TYPE: EST

STATUS: New

CONT_NAME: Santos E

CITATION: Identification of differentially expressed genes in response to Mycosphaerella fijiensis in the resistant Musa accession Calcutta 4 using suppression subtractive hybridization.

LIBRARY: SSH Calcutta M. fijiensis library

EST#: 2F8 BMCIBE

DNA_TYPE: cDNA

PUBLIC:

COMMENT: Raw sequences were clear up, from vector and cloning adaptors, with the program CLC Genomics Workbench (CLC bio, Denmark).

SEQUENCE:

CGTTGTGCTTGTTGCCTCTCGACATGTCGACCTGCGGCAACTGCGACTGCGCTGACAAGA

GCCAGTGCGTGAAGAAGGGAAACAGCTACGCTACCGAGACTGTTGCGACCGAGAAGAGGT

ATTATTGATCTTGTCCGCATGATGAAATTCCACACAACATGACTCAGCAAACATGATCCT

TTTATTCTTGAAAAAACAACTCAAAAAAGAAGTAAAAAAAACAGAGAATATATCTGCGAT

TATTTCTTTTTGAGTGATGTGGAACTCCATGCCATAGCTTAAAACTATTTTCAAAGTAAT

GATCAAACCTACCGATGGGCGGCGTATCCCGTTGT

||

TYPE: EST

STATUS: New

CONT_NAME: Santos E

CITATION: Identification of differentially expressed genes in response to Mycosphaerella fijiensis in the resistant Musa accession Calcutta 4 using suppression subtractive hybridization.

LIBRARY: SSH Calcutta M. fijiensis library

EST#: 2G1 BMCIBE

DNA_TYPE: cDNA

PUBLIC:

COMMENT: Raw sequences were clear up, from vector and cloning adaptors, with the program CLC Genomics Workbench (CLC bio, Denmark).

SEQUENCE:

CTCTGTTCTTCGACGGTTGTCCCCGACTCCCTCTTTTCCCACCGTGCCATTCCCCTGCCC

GATCCCAAAAACCCTTGCTGTAGCCGGCGAAAGCAAGGAGAAAGCGAGATAGATCTAGGA

AAGCGAAAGCAATAATCAAGCATCCTTTGGTAATCTGAGGTAGTGCGAGTTCGTTACGAA

GCCATTTCCTGTAGAATCTGGAGGTTGCTAGGGGGGAGAGGGGCTGTTGTTGCTGTTGCG

GAGATGAGCTACGGAAGCAGCTCGATTGCTTCAGGTGCTAAGACTGCTGCAAGGGCGTTT

GACTTTGGAAGGACCCATGTGGTCAGACCTAAGGGGAGGTACCTGGCCACTGTTGTGTGG

CTTCATGGCCTAGGTGATAATGGAGCAAGCTGGTCCCAACTCCTGGAAACCCTCCCTCTT

CCAAATATTAAGTGGATATGCCCAACTGCCCCTACCAGGCCTGTAGCAGTTTTTGGTGGA

TTTCCTTCAACTGCTTGGTTTGACGTTGGGGATCTGTCAGAGGATGGCCCTGATGATGTT

GAAGGAATGGATGCATCAGCTGCACATGTTGCAAATTTATTGTCGACAGAGCCTGCTGAT

ATCAAACTTGGCGTTGGTGGCTTTAGTATGGGTGCTGCCA

||

TYPE: EST

STATUS: New

CONT_NAME: Santos E

CITATION: Identification of differentially expressed genes in response to Mycosphaerella fijiensis in the resistant Musa accession Calcutta 4 using suppression subtractive hybridization.

LIBRARY: SSH Calcutta M. fijiensis library

EST#: 2G2 BMCIBE

DNA_TYPE: cDNA

PUBLIC:

COMMENT: Raw sequences were clear up, from vector and cloning adaptors, with the program CLC Genomics Workbench (CLC bio, Denmark).

SEQUENCE:

GGAATTTTTGAATCACATTATCAAGTCCACAAACATGAAGTGCCTCACACCACTCTCGGC

CTTGGATGGGGAGTGTGGGTTTCTTGCTGCTAACCTATACGCAAGGAGTGTCTTTGGAGA

GGACGCTCTGGTCAATGTGAGTGTCGAGAAGCAGGCAGATGGAAAGCTCAGTGGTTACAT

CCGGATACGGAGCAAGACACAAGGAATTGCTCTGAGTTTAGGGGATAAGATCACTCTCAA

ACAGAAGGGTGGTAGTTAGTAAGAACCATATCTAGTCTATTCGCTCCAACAT

||

TYPE: EST

STATUS: New

CONT_NAME: Santos E

CITATION: Identification of differentially expressed genes in response to Mycosphaerella fijiensis in the resistant Musa accession Calcutta 4 using suppression subtractive hybridization.

LIBRARY: SSH Calcutta M. fijiensis library

EST#: 2G3 BMCIBE

DNA_TYPE: cDNA

PUBLIC:

COMMENT: Raw sequences were clear up, from vector and cloning adaptors, with the program CLC Genomics Workbench (CLC bio, Denmark).

SEQUENCE:

GCCGGGGACGCCTAGGATGATGAGGCCGCACGGCTCCGGGCCGAGAACCTCCGTCTGAGA

GCCATACTCGCCGACAATCTTGCAATCCTGCAAACCATCTATGATTCGCCGACTCTCTCC

AGCGATTGCCCTCCCGATTTGCATTCCCGCCTTTTAGCTGCAGTTGGGAACTCGAGCTTT

CTGGGAGAACTTGAATTGCTTCGTAAAGGGTCAAAAGACATTCCGAATTGTG

||

TYPE: EST

STATUS: New

CONT_NAME: Santos E

CITATION: Identification of differentially expressed genes in response to Mycosphaerella fijiensis in the resistant Musa accession Calcutta 4 using suppression subtractive hybridization.

LIBRARY: SSH Calcutta M. fijiensis library

EST#: 2G4 BMCIBE

DNA_TYPE: cDNA

PUBLIC:

COMMENT: Raw sequences were clear up, from vector and cloning adaptors, with the program CLC Genomics Workbench (CLC bio, Denmark).

SEQUENCE:

CCACAGTCGTTGCGGTGGAATTAAGGGGCTTATGTCTATCAAAGAAGATGGCACCAGAAG

CAGGTAAGAAACATCACCTCTGTTTTCATCTTTCAGTAATTGGCAGCATTAGTGTTGACT

CTTTTTTCTTGAAAGATGAAATAGATTGGAATTTCCTCCAAATTTCTTGGGAATTCCGAC

CAAATGATCCTCCTTATCTGGTTGTGATCAGTGCCTTCATAGAGGACTGGGTGAAGGTCT

GCTTGCCAGCATTGGAGAAGGTGAAAGCAAATCACTCAGCCTTGCCCTTCGAGGATCAAT

GCACCCATTGCGAAAAGGTGTTCTTCTCTTCCTTTTTTACAATGATATTTTCTAGTTCAT

ACAAGTAAGCAATGTGCAAAGGTTACATTCACCTTCTCTCAAGCTAATTTTAGCAGATGT

AAAATTGCTACTGCTTCTTGGCTCTGAGGATAGTGGAATATGATGATTACTGTCAACAGC

AGCAGAGAAGACTTTTTTTGGTTGAAATGTTTTGAGCTGTTGATATCGGAAAGAGAAGTT

TGCTTTTGGCTATTGTGTAGCTGACTGATGCCATTCTCTTCGACCCCTGGCAGGAGGCAG

TGAACGTCTCTCTCCACAACCCTGAAGACGTATCCCTTCGTCAAAGATGGACTGGAGAAN

AAAACACTGAAACTGATCGGGGCACATTTACAACTTCGTTGCCGGCAGCTTTGACATCTG

GGAGGTATGAACGGAGGCTTAAAGGANCTCTCATTGTTGTTGT

||

TYPE: EST

STATUS: New

CONT_NAME: Santos E

CITATION: Identification of differentially expressed genes in response to Mycosphaerella fijiensis in the resistant Musa accession Calcutta 4 using suppression subtractive hybridization.

LIBRARY: SSH Calcutta M. fijiensis library

EST#: 2G5 BMCIBE

DNA_TYPE: cDNA

PUBLIC:

COMMENT: Raw sequences were clear up, from vector and cloning adaptors, with the program CLC Genomics Workbench (CLC bio, Denmark).

SEQUENCE:

ACCGATGCTAAAATGTCAGCTTTTATGAGATTCCAAAGTCCTACCAAGCATCTCCTGTTT

GGTTCTTTTCACAACCGCCCTTCATGCTTTATTTCTGAAGGATCTAAAAGACCTTCCAAG

ACAACAAGCTGATCCAACAAAGCCAGCTTCTCATTTTCCAGAGTTTTCACTTCTGCATCA

GCTTCGCTGAGTTGGCTGTCCAGTGAGAAGCTTTCTTCCTCCAGCAACAGATACTTGTGT

CTTAA

||

TYPE: EST

STATUS: New

CONT_NAME: Santos E

CITATION: Identification of differentially expressed genes in response to Mycosphaerella fijiensis in the resistant Musa accession Calcutta 4 using suppression subtractive hybridization.

LIBRARY: SSH Calcutta M. fijiensis library

EST#: 2G6 BMCIBE

DNA_TYPE: cDNA

PUBLIC:

COMMENT: Raw sequences were clear up, from vector and cloning adaptors, with the program CLC Genomics Workbench (CLC bio, Denmark).

SEQUENCE:

GTTCATACTAATGGACCTATATTAGATGAAATTATACTTTCTGATCCTGAGATATCCAAT

GCAATTGAGAATGAAAAAGAGGTCAATAAAACTGTAAAGATTTATAATGTTGATCGTGCT

GTTTGTGGTCGCATAGCTGGTGTTATAGCCAAGAAGTATGGAGATGTAGGTTTTGCGGGG

CAGCTTAATCTAACATTTATTGGAAGTGCTGGACAATCCTTTGCTTGCTTCTTGACTCCT

GGAATGAATATTCGACTAGTTGGGGAAGCTAATGATTATGTTGGAAAGGGTATGGCTGGT

GGAGAGCTGGTAGTAACACCTGTTGATGACACTGGTTTCTGCCCGGAGGATGCTACAATA

GTAGGTAACACCTGTCTATATGGTGCAACTGGTGGTCAAATCTTTGTGAGAGGGAAAGCA

GGTGAGCGATTTGCCGTTAGAAATTCTCTTGTGGAAGCTGTGGTTGAGGGCACTGGGGAT

CATTGTTGTGAGT

||

TYPE: EST

STATUS: New

CONT_NAME: Santos E

CITATION: Identification of differentially expressed genes in response to Mycosphaerella fijiensis in the resistant Musa accession Calcutta 4 using suppression subtractive hybridization.

LIBRARY: SSH Calcutta M. fijiensis library

EST#: 2G7 BMCIBE

DNA_TYPE: cDNA

PUBLIC:

COMMENT: Raw sequences were clear up, from vector and cloning adaptors, with the program CLC Genomics Workbench (CLC bio, Denmark).

SEQUENCE:

TGCTAGTGCACGCCTTCGACTGGAGCCTTCCCGACG

GCGAGGAGCTCAACATGGACGAGAAGTTTGGCCTGGCTCTTCCCAAGGCTGTGCCTCTCA

AAGTTTTTCTGCGCCCGCGCCTGTCGCCGGCGGCCTACGCCTGATCTCCCTCTCTATCGA

TCTATCTATCTATCTCTCGT

||

TYPE: EST

STATUS: New

CONT_NAME: Santos E

CITATION: Identification of differentially expressed genes in response to Mycosphaerella fijiensis in the resistant Musa accession Calcutta 4 using suppression subtractive hybridization.

LIBRARY: SSH Calcutta M. fijiensis library

EST#: 2G12 BMCIBE

DNA_TYPE: cDNA

PUBLIC:

COMMENT: Raw sequences were clear up, from vector and cloning adaptors, with the program CLC Genomics Workbench (CLC bio, Denmark).

SEQUENCE:

ACCCATATGCAAAGAAGTATTTGCATCAATTAATTGCCACTGAATGAGAATTACAGCAAA

TGCCTGCTTGCAACAACATCATCTCTAGTTCACTAGCATGCAGTCATAAGAACCTCTCCT

GTGGTCTCTTCCAGTTTACAAGGTGAGCTTTTCCATCTCCCTCCTCAAGGCGGTGGCTCT

CACGAGGCTGCCGATGGTGTTCACGGCGAGCTTGAGGTCGTCGAGGGGGTTCCCCGGCAC

GACCTTCTTGT

||

TYPE: EST

STATUS: New

CONT_NAME: Santos E

CITATION: Identification of differentially expressed genes in response to Mycosphaerella fijiensis in the resistant Musa accession Calcutta 4 using suppression subtractive hybridization.

LIBRARY: SSH Calcutta M. fijiensis library

EST#: 2H2 BMCIBE

DNA_TYPE: cDNA

PUBLIC:

COMMENT: Raw sequences were clear up, from vector and cloning adaptors, with the program CLC Genomics Workbench (CLC bio, Denmark).

SEQUENCE:

ACACGATCTCAGTAATCTCTCCATCAATGGACATCTCCACCTTCTTCTTCGTTGGGCAAT

CGGGATAAGTGCACTTGTAATAGCCGACCGGGTTCTCGCTTCCCTTAATCTGTTTCTGGC

CATACTTCCTCCAGTTGTATCCATCATCTGATCTCCTCTGCCCTCTCACGGCCTGAGAAG

AACTTGCACGAATTGGGACAACATCTTCAACTTTGACTTCGGGTTTTCGTTCTCCTCCCT

GGGTTTGGAAACAGAAGTCGGAGTAGGCTCCATCCTGTGAATTAGCAGAGAAGCTCCAGT

TCAGATGCTGTGCAGGGAGAGAACCAGTGGTTGGAGAAGGCAACATAGAGGAAGAGGGGA

GGAGGAGTGGAGATTCCAAGAGCTCGGCAAGGCTGAGGCTGGTTGGGAAAGAGAAGGAAG

CAGATGGAGGAGGAGGGGAGAGAGGCAGGAAGGGAGGAGGTGCAGACTTGAACTTGGATG

TGCCACCTGTATCACCCCCTGCCAATTCCCGGCTCATCTCA

||

TYPE: EST

STATUS: New

CONT_NAME: Santos E

CITATION: Identification of differentially expressed genes in response to Mycosphaerella fijiensis in the resistant Musa accession Calcutta 4 using suppression subtractive hybridization.

LIBRARY: SSH Calcutta M. fijiensis library

EST#: 2H5 BMCIBE

DNA_TYPE: cDNA

PUBLIC:

COMMENT: Raw sequences were clear up, from vector and cloning adaptors, with the program CLC Genomics Workbench (CLC bio, Denmark).

SEQUENCE:

ACAATAAGATTATGCCTGAACTCACTCTGACCAGAAAACACAAGCTAGGGAGGCAAAAAA

TGCGAGTCTGTTTTGCTTTCAGCCAATGACATTGGTTCACAAGCCTCACACCCGCTCGAA

TGGAAAACACCTAAAACGAATACCTCATTCAAAATCTGAGACCTGGCTGCCTTATGTCTC

CCTCCTAGATCTCCTGGGGCGCGGGGCTGATGCATCATCTTTCTCGTTCTCCTCTCTCTC

CTCGCTGCCTCCTGTAGCTTCAGTCTCAGCAGCTCCAGAGTTGTTAGTTTCAGTGGTTGG

TGCAACTGGGGCCTGAGGCTTCTTCCCGCCAAAAAGAATCCTGAATATGACAGTAAGAAT

TACCACCAGAATGGATGCCAGGATCCCAATAGTGAGGTTAGGTTGTTTCTCTGCCTTCTC

AATGACATCAATAATTTTGATCTTGTGGGACTCCAAGAAGGGAATGTCTGCAAACTTGTA

AAGAACATCAAATACTTTCTTCTGGAAACCTGAAAGGCCATCTGAAGATACGGAAGCATC

TTCAGCCTTCTGCTTTTCTTTCTCAACTTCATATTTAGGCTTCCATGTCT

||

TYPE: EST

STATUS: New

CONT_NAME: Santos E

CITATION: Identification of differentially expressed genes in response to Mycosphaerella fijiensis in the resistant Musa accession Calcutta 4 using suppression subtractive hybridization.

LIBRARY: SSH Calcutta M. fijiensis library

EST#: 2H6 BMCIBE

DNA_TYPE: cDNA

PUBLIC:

COMMENT: Raw sequences were clear up, from vector and cloning adaptors, with the program CLC Genomics Workbench (CLC bio, Denmark).

SEQUENCE:

ACAAGCTTTTTTTTTTTTTTTTTTTTTTTTTTTTTTTTAGGAAAAATGACCGAAAAAAAT

AACTTTCATCAATGTTACGGTTCCACTGACAATCAAAATTGTTACCTAAACATAACCAAA

CCATTAAAAAGAATTAGTCTTCTCCAACACAGATGAATAAAACTTTTAGGAAATGACTTC

AAGATCGAAAGACAACATCAAAGAGGATTGTTAAGCTCATCGCTTCTTAAAGACACCAAC

AGTCTTTCCCCTCCTGCCAGTTGTCTTGGTGTGCTGACCGCGAACTCGAAAACCCCAGAA

GTGACGGAAACCACGGGGGTTCCTTATCTTCTTCAGTCTCTCAAGGTCATCCCTCAGCTT

CATGTCTAAGGCATTTGAAACAACCTGAGAATATCGTCCATCCTTGTAGTCTTTCTTCCT

GTTCAAGAACCAATCCGGGATCTTGAACTGGCGCGGATTGGCAACAATTGTCATCAAGTT

CTCGAGTTCGGCAGCCGAAATCTCACCAGCCCTCTTATTCATGTCGACGTCGGCCTTCTT

GCAGACGATGTTCGCGAAGCGGCGGCCGATACCCTTGATAGAGGTGAGAGCGAACATGAT

CTTCTGCTTCCCATCGACGTTCGTGTTCAGAACACGAAGAATGTGCTGGAAATCCTCGTT

CGCGACCAGCGACATGGCAGCGGCTAANGTTTCGCTGTCCGCCCTACGTCTGATCTCGCT

TCTCTCCCTATG

||

TYPE: EST

STATUS: New

CONT_NAME: Santos E

CITATION: Identification of differentially expressed genes in response to Mycosphaerella fijiensis in the resistant Musa accession Calcutta 4 using suppression subtractive hybridization.

LIBRARY: SSH Calcutta M. fijiensis library

EST#: 2H7 BMCIBE

DNA_TYPE: cDNA

PUBLIC:

COMMENT: Raw sequences were clear up, from vector and cloning adaptors, with the program CLC Genomics Workbench (CLC bio, Denmark).

SEQUENCE:

ACCCACCAAGAGTAGCAAACATCGGCAAACTTCTCAGGACGGCCATTCAATCCTCCATCT

TTACATTGCCGCTCACACAACCACCAACCAAGCAGGTCCCTGTCGACATGGTGCAGGGAA

CCCGTGATTGCAAGCGCACCGACACAACAGAAGATCTGGCCAGCATGTGACTCCCCACCG

GGTATAGACCCAAATCCACCGTCTAGGTTTTTGCAGCTGACAATGTAATTTACAGCCTTT

TCCACATTGATTTTACTTAGAC

||

TYPE: EST

STATUS: New

CONT_NAME: Santos E

CITATION: Identification of differentially expressed genes in response to Mycosphaerella fijiensis in the resistant Musa accession Calcutta 4 using suppression subtractive hybridization.

LIBRARY: SSH Calcutta M. fijiensis library

EST#: 2H10 BMCIBE

DNA_TYPE: cDNA

PUBLIC:

COMMENT: Raw sequences were clear up, from vector and cloning adaptors, with the program CLC Genomics Workbench (CLC bio, Denmark).

SEQUENCE:

ACCAGGGGCCCGCCCGACGTGCCTCTTCCTCACCTAC

TCCAATGAACCATTATGTCCTGTTTTTTTCCCCTGTGCTGCCGCTTGTCGTGTACCATCC

CACGCCATGAATTGTTTCTTCGAGGAGCTGGGCTTGACTTACCAGGAAATCCTGTTTCTC

TTGCTGCATGCTGATGAACTCAAAGGGGTTACATCATTGATGCAGCTGAACGTCATGCCC

CCCTTTGGGCTCATAATGGAAGGCCCAGGGCTTGACGGCATTGTCGGGGCCACTCCGGAN

CCAATAAGGAAGAGGATCGATGGTTACGTTCTTCTTGGCCCTCCCTTTTATGGTCCACGA

CTGGGAAATCCCTGCCGATACCCCCCTCCACCCTGTTCCTTGCTCTCCC

||

TYPE: EST

STATUS: New

CONT_NAME: Santos E

CITATION: Identification of differentially expressed genes in response to Mycosphaerella fijiensis in the resistant Musa accession Calcutta 4 using suppression subtractive hybridization.

LIBRARY: SSH Calcutta M. fijiensis library

EST#: 2H11 BMCIBE

DNA_TYPE: cDNA

PUBLIC:

COMMENT: Raw sequences were clear up, from vector and cloning adaptors, with the program CLC Genomics Workbench (CLC bio, Denmark).

SEQUENCE:

TCACAACCAGAGGTCATGGAGCTGTCATAAATATTT

ACAGATTATCTCCATTTCAGTTCCCTGCTTTCAATTTGGGTGACTATTTTATTGATAAAA

TTACTCCAGAATCTGGATGATAGCAGCTACCTTCCTACCATCTCGTTCCCCTAGCAGCTT

CTTGGTCACAGATGACATTGCATGTTGCAAGGT

||

TYPE: EST

STATUS: New

CONT_NAME: Santos E

CITATION: Identification of differentially expressed genes in response to Mycosphaerella fijiensis in the resistant Musa accession Calcutta 4 using suppression subtractive hybridization.

LIBRARY: SSH Calcutta M. fijiensis library

EST#: 2H12 BMCIBE

DNA_TYPE: cDNA

PUBLIC:

COMMENT: Raw sequences were clear up, from vector and cloning adaptors, with the program CLC Genomics Workbench (CLC bio, Denmark).

SEQUENCE:

CCCTCAACAACTGGGCCACCCACCTGAGCGACCCAC

TCCACACCACCATCATCGACAACGTCTTCTTCGCCTCCTCCTCCTAAGTGCGACAAGACA

TCGATCGGCTTCACCTTTCCCCTCCCTCCCATGGCGATCTCCTTGTGTGGGATTCCTGTG

GTGGGGGTTAATTTCCTGTGT

||
