# Supplementary material for: Identification of Differentially-Expressed Genes in Response to Mycosphaerella fijiensis in the Resistant Musa Accession ‘Calcutta-4’ Using Suppression Subtractive Hybridization
Source: PLoS One. 2016 Aug 3;11(8):e0160083. doi: 10.1371/journal.pone.0160083 (PMC4972352; doi:10.1371/journal.pone.0160083)
Supplement: S1 Table — (PDF) [file pone.0160083.s002.pdf]

S1 Table. Sequence similarity search of the ESTs obtained from the cDNA subtracted library to the banana genome sequence using blastn (http://banana-genome.cirad.fr/).

Clone	BANANA GENOME							
	blastn						Coding protein in the homology region of the query in the banana genome using gbrowse	
	Chromosome	Start	End	Strand	Evalue	% Identity	Name	Description/function
1A1	chr6	28983573	28983928	-	1E-78	100	GSMUA_Achr6P28670_00	GSMUA_Achr6T28670_001~ Light-regulated protein~ LIR1~ modules
1A3	chr10	30215884	30216201	+	2E-70	98,6	GSMUA_Achr10P26880_001	GSMUA_Achr10T26880_001~ Putative Zinc finger protein CONSTANS-LIKE 16~ COL16~ complete
1A7	chr10	32034433	32034646	-	1E-115	99,53	GSMUA_Achr10P29540_001	GSMUA_Achr10T29540_001~ Formate--tetrahydrofolate ligase~ THFS~ complete
1B3	chr7	8858019	8858520	+	6E-62	100	GSMUA_Achr7P11100_001	Beta-amylase 3, chloroplastic~ BAM3~ complete
1B4	chr6	15073662	15073864	+	4E-99	97,54	GSMUA_Achr6P20490_001	GSMUA_Achr6T20490_001~ Putative uncharacterized protein~ EP300~ fragment
1B5	chr7	3434854	3435440	+	0	98,3		
1B9	chr11	745843	746191	-	2E-92	99,43	GSMUA_Achr11P01090_001	GSMUA_Achr11T01090_001~ Serine carboxypeptidase-like 18~ SCPL18~ complete
1B10	chr4	21424882	21425154	-	1E-115	92,67	GSMUA_Achr4P20880_001	GSMUA_Achr4T20880_001~ expressed protein~ TCHHL1~ complete
1C3	chrUn_random	3933750	3934193	-	4E-89	94,74	GSMUA_AchrUn_randomP01190_001	PREDICTED FUNCTION: Ankyrin repeat domain-containing protein, chloroplastic
1C7	chr4	26380220	26380391	+	8e-56	97,67	GSMUA_Achr4G27630_001	GSMUA_Achr4T27630_001~ Putative Protein SYM1~ SYM1~ modules

1C8	chr9	29437197	29438438	-	0	97,44	GSMUA_Achr9P24740_001	GSMUA_Achr9T24740_001~ 22.3 kDa class VI heat shock protein~ HSP22.3~ modules; GSMUA_Achr9T24750_001~ Oxygen-evolving enhancer protein 3-2, chloroplastic~ PSBQ2~ complete
1C9	chr8	5439390	5439812	+	0	98,35	GSMUA_Achr8P08070_001	GSMUA_Achr8T08070_001~ V-type proton ATPase 16 kDa proteolipid subunit c1/c3/c5~ VHA-C1~ complete
1C10	chr5	6132897	6133428	-	0	99,62	GSMUA_Achr5P08410_001	GSMUA_Achr5T08410_001~ Putative Protein GPR107~ Gpr107~ fragment
1D2	chr11	24130202	24130640	-	0	97,72	GSMUA_Achr11P24730_001	GSMUA_Achr11T24730_001~ Zinc finger CCCH domain-containing protein 33~ Os05g0128200~ complete
1E1	chr7	25585417	25585986	+	1E-158	99,31	GSMUA_Achr7P23330_001	GSMUA_Achr7T23330_001~ no apical meristem protein, putative, expressed~ NAC078~ complete
1E3	chr5	2701715	2702692	+	1E-156	99,65	GSMUA_Achr5P03850_001	GSMUA_Achr5T03850_001~ Uncharacterized protein At1g03900~ At1g03900~ complete
1E6	chr9	12689047	12689910	+	1E-127	100	GSMUA_Achr9P18120_001	GSMUA_Achr9T18120_001~ Putative Uncharacterized protein yqjG~ yqjG~ modules
1E7	chr2	16494498	16494722	+	1E-119	99,11	GSMUA_Achr2P14960_001	GSMUA_Achr2T14960_001~ Chlorophyll a-b binding protein 4, chloroplastic~ LHCA4~ complete
1E8	chr1	7231159	7232974	-	1E-159	98,99	GSMUA_Achr1P09400_001	GSMUA_Achr1T09400_001~ Glucose-1-phosphate adenylyltransferase small subunit, chloroplastic~ AGPS1~ complete
1F11	chr11	22726181	22727063	+	1E-131	99,18	GSMUA_Achr11P22440_001	GSMUA_Achr11T22440_001~ Probable serine/threonine-protein kinase At4g35230~ At4g35230~ fragment
1G10	chr4	1175651	1175797	-	1E-28	97,26	GSMUA_Achr4P01380_001	GSMUA_Achr4T01380_001~ Malate dehydrogenase, glyoxysomal~ MDHG~ complete

1B12	chr6	17015937	17016383	+	1E-135	99,21	GSMUA_Achr6P21320_001	GSMUA_Achr6T21320_001~ Salt tolerance protein~ STO~ complete
1C2	chr9	28759261	28759665	-	1E-134	98,82	GSMUA_Achr9P23930_001	GSMUA_Achr9T23930_001~ UTP--glucose-1-phosphate uridylyltransferase~ UGPA~ complete
1D8	chr5	2552022	2552414	-	1E-130	98,05	GSMUA_Achr5P03660_001	GSMUA_Achr5T03660_001~ phosphoribulokinase/Uridine kinase family protein, expressed~ At1g32060~ complete
1D11	chr7	8794936	8795220	+	1E-127	95,09	GSMUA_Achr7P10930_001	GSMUA_Achr7T10930_001~ Auxin-responsive protein IAA27~ IAA27~ complete
1E4	chrUn_random	70954961	70955197	+	1E-114	97,05	*	
1F8	chrUn_random	123816337	123816711	+	0	100	**	
1G5	chr3	23245517	23245189	-	2E-82	86,32	GSMUA_Achr3P22310_001	GSMUA_Achr3G22310_001~ Putative disease resistance RPP13-like protein 1~ RGA1~ missing_completeness
2A2	chr9	10334989	10335278	+	1E-160	99,66	GSMUA_Achr9P15630_001	GSMUA_Achr9T15630_001~ Putative Protein TIFY 6B~ TIFY6B~ complete
2A4	chrUn_random	101014989	101015172	-	3E-97	99,46	GSMUA_AchrUn_randomP20820_001	GSMUA_AchrUn_randomP20820_001
2A6	chr9	29262330	29264458	+	6E-60	100	GSMUA_Achr9P24480_001	GSMUA_Achr9T24480_001~ ATP-dependent Clp protease proteolytic subunit-related protein 4, chloroplastic~ CLPR4~ complete
2A9	Chr4	15050206	15050330	+	4e-30	89,76		
2A10	chr3	6915216	6915410	+	6E-27	96,05	GSMUA_Achr3P09560_001	GSMUA_Achr3T09560_001~ Putative Repressor of RNA polymerase III

								transcription MAF1 homolog~ MAF1~ fragment
2A11	chr6	8595226	8595826	+	1E-174	99,37	GSMUA_Achr6P12860_001	GSMUA_Achr6T12850_001~ Hypothetical protein~ Ndel1~ missing_functional_completeness; GSMUA_Achr6T12860_001~ Hypothetical protein~ unknown_gene~ missing_functional_completeness
2B1	chr9	1101616	1101910	-	3E-82	100	GSMUA_Achr9P01470_001	GSMUA_Achr9T01470_001~ Chlorophyll a-b binding protein 7, chloroplastic~ CAB7~ complete
2B4	chrUn_random	123816337	123816721	-	0	100	***	
2B6	chr6	32312511	32314046	+	9E-69	94,77	GSMUA_Achr6P33160_001	GSMUA_Achr6T33160_001~ Protein IN2-1 homolog B~ GSTZ5~ complete
2B8	chr11	23925951	23926418	+	1E-121	100	GSMUA_Achr11P24320_001	GSMUA_Achr11T24320_001~ Ribulose bisphosphate carboxylase/oxygenase activase 2, chloroplastic~ RCA2~ complete
2B9	chr11	20339360	20341217	-	2E-76	100	GSMUA_Achr11P19110_001	Glyceraldehyde-3-phosphate dehydrogenase, cytosolic 3~ GAPC3~ modules
2B10	chr9	7550185	7550930	+	1E-173	98,77	GSMUA_Achr9P11610_001	GSMUA_Achr9T11610_001~ Putative Protein TRANSPARENT TESTA 12~ TT12~ complete
2B11	chr5	9797297	9797677	-	0	99,48	GSMUA_Achr5P13610_001	GSMUA_Achr5T13610_001~ UPF0496 protein 4~ OsI_033149~ fragment

2B12	chr1	13052506	13053311	-	1E-54	100	GSMUA_Achr1P17550_001	GSMUA_Achr1T17550_001~ Catalase isozyme 2~ CAT2~ fragment
2C1	chrUn_random	12130474	12130559	-	8E-31	94,19		
2C5	chr6	26856514	26856625	-	2E-50	97,32	GSMUA_Achr6P25880_001	GSMUA_Achr6T25880_001~ Putative NAD-dependent ADP-ribosyltransferase sirtuin-4~ SIRT4~ modules
2C7	chrUn_random	42512360	42512704	-	4E-84	100	GSMUA_AchrUn_randomP09150_001	GSMUA_AchrUn_randomT09150_001~ Putative peroxisomal-coenzyme A synthetase~ SPCC1827.03c~ complete
2C8	chr11	14193136	14193501	-	0	97,81	GSMUA_Achr11P13450_001	GSMUA_Achr11T13450_001~ Hypothetical protein~ AF_0788~ missing_functional_completeness
2C9	chrUn_random	117571845	117572238	-	0	99,24	GSMUA_AchrUn_randomT23890_001	GSMUA_AchrUn_randomT23890_001~ Putative Cellulose synthase-like protein G3~ CSLG3~ fragment
2C10	chr2	19078834	19079103	-	E-135	92,65	GSMUA_Achr2G18820_001	GSMUA_Achr2T18820_001~ Putative Protein RIC1 homolog~ Kiaa1432~ fragment
2C11	chr10	16215253	16216324	-	e-111	95,77	GSMUA_Achr10P05740_001	GSMUA_Achr10T05740_001~ Putative Haloalkane dehalogenase 2~ dhmA2~ modules
2C12	chr3	20217521	20220433	-	2E-73	99,31	GSMUA_Achr3P18840_001	GSMUA_Achr3T18840_001~ Somatic embryogenesis receptor kinase 1~ SERK1~ fragment
2D1	chr7	27168512	27169617	+	1E-57	99,15	GSMUA_Achr7P25580_001	GSMUA_Achr7T25580_001~ Putative glucose-6-phosphate 1-epimerase~ YMR099C~ modules

2D3	chr6	33541360	33541672	+	e-171	97,44	GSMUA_Achr6P35290_001	GSMUA_Achr6T35290_001~ expressed protein~ mx~ complete
2D5	chr10	21970294	21970364	+	1E-25	95,77	GSMUA_Achr10P13230_001	GSMUA_Achr10T13230_001~ Elongation factor 1-alpha~ TEF1~ fragment
2D6	chr2	17229995	17230522	-	0	99,52	GSMUA_Achr2P16250_001	GSMUA_Achr2T16250_001~ Heat shock cognate 70 kDa protein~ HSP70~ fragment
2D7	chr8	16727860	16728300	-	1E-117	99,54	GSMUA_Achr8P17280_001	GSMUA_Achr8T17280_001~ Fructose-bisphosphate aldolase, chloroplastic~ Os11g0171300~ complete
2D10	chr3	1817893	1819721	+	0	99,35	GSMUA_Achr3P02780_001	GSMUA_Achr3T02780_001~ Chlorophyll a-b binding protein CP26, chloroplastic~ LHCB5~ complete
2D11	chr1	16090193	16091595	-	2E-94	99,44	GSMUA_Achr1P21370_001	GSMUA_Achr1T21370_001~ Transcription initiation factor IIB~ TFIIB~ complete
2D12	chr5	8718524	8718883	-	1E-171	96,4	GSMUA_Achr5P12030_001	GSMUA_Achr5T12030_001~ Putative LanC-like protein 2~ Lancl2~ complete
2E1	chr10	1199115	1199619	+	9e-88	98,02	GSMUA_Achr10P00610_001	GSMUA_Achr10T00610_001~ Putative expressed protein~ unknown_gene~ complete
2E2	chrUn_random	17934166	17934481	-	e-164	97,5	GSMUA_AchrUn_randomP04190_001	GSMUA_AchrUn_randomT04190_001~ expressed protein~ pheT~ complete
2E3	chr3	2617161	2617502	+	e-179	96,78	GSMUA_Achr3P04110_001	GSMUA_Achr3T04110_001~ Chlorophyll a-b binding protein 8, chloroplastic~ CAB8~ complete

2E4	chr2	11068977	11069082	+	8E-42	95,28	GSMUA_Achr2P06190_001	GSMUA_Achr2T06190_001~ Putative UPF0420 protein C16orf58 homolog~ C16orf58~ fragment
2E6	chr6	396916	397630	+	0	99,1	GSMUA_Achr6P00600_001	GSMUA_Achr6T00600_001~ Putative serine/threonine-protein kinase receptor~ SRK6~ complete
2E7	chr4	10870225	10870384	-	4E-71	97,55	GSMUA_Achr4P14550_001	GSMUA_Achr4T14550_001~ Hypothetical protein~ GRP7~ missing_functional_completeness
2E8	chr3	5964051	5965393	+	e-171	96	GSMUA_Achr3P08390_001	GSMUA_Achr3T08390_001~ ABC transporter I family member 20~ ABCI20~ complete
2E9	chr2	16130033	16130294	+	1E-123	96,23	GSMUA_Achr2P14320_001	GSMUA_Achr2T14320_001~ Isoflavone reductase homolog~ IRL~ complete
2E10	chr5	4575829	4576202	+	0	98,66	GSMUA_Achr5P06180_001	GSMUA_Achr5T06180_001~ Putative pleiotropic drug resistance protein 7~ PDR7~ complete
2E11	chr3	19835729	19835972	+	1E-130	99,18	GSMUA_Achr3P18360_001	GSMUA_Achr3T18360_001~ expressed protein~ Dync2h1~ complete
2E12	chrUn_random	70974586	70974752	+	6e-92	100	****	
2F1	chr6	4620097	4620570	-	8E-75	100	GSMUA_Achr6P06810_001	GSMUA_Achr6T06810_001~ Predicted protein~ Glyctk~ complete
2F2	chr10	22718648	22718949	-	1E-165	99,34	GSMUA_Achr10P14480_001	GSMUA_Achr10T14480_001~ Uncharacterized protein At4g01050~ At4g01050~ complete

2F3	chr8	26943234	26944937	+	1E-169	98,44	GSMUA_Achr8P22320_001	GSMUA_Achr8T22320_001~ ATP-dependent Clp protease proteolytic subunit 5, chloroplastic~ CLPP5~ complete
2F4	chr9	5413959	5415449	-	3E-46	98,99	GSMUA_Achr9P08450_001	GSMUA_Achr9T08450_001~ Alcohol dehydrogenase 2~ ADH2~ complete
2F7	chr6	1307033	1307315	+	1E-156	99,65	GSMUA_Achr6P02020_001	GSMUA_Achr6T02020_001~ Elongation factor 1-alpha, putative, expressed~ EF1~ modules
2F8	chr6	27042062	27042533	-	6e-59	98,36	GSMUA_Achr6P26180_001	GSMUA_Achr6T26180_001~ Metallothionein-like protein type 3~ MT3A~ complete
2G1	chr2	19670104	19672708	+	3E-78	98,14	GSMUA_Achr2P19690_001	GSMUA_Achr2T19690_001~ Putative Acyl-protein thioesterase 2~ LYPLA2~ complete
2G2	chr7	12845913	12846386	-	1E-132	99,19	GSMUA_Achr7P15960_001	GSMUA_Achr7T15960_001~ Coatomer subunit beta-1~ Os11g0174000~ fragment
2G3	chr1	16053104	16053995	+	5E-65	97,83	GSMUA_Achr1P21330_001	GSMUA_Achr1T21330_001~ Putative expressed protein~ Pkhd1l1~ complete
2G4	chr4	11213343	11213954	-	0	97,88	GSMUA_Achr4P14900_001	GSMUA_Achr4T14900_001~ Hypothetical protein~ CA2~ missing_functional_completeness
2G5	chr10	23470756	23471006	+	1E-130	98,41	GSMUA_Achr10P15590_001	GSMUA_Achr10T15590_001~ Putative expressed protein~ YKL214C~ complete
2G6	chr2	11530055	11531710	+	1E-106	98,55	GSMUA_Achr2P06800_001	GSMUA_Achr2T06800_001~ Ferredoxin-dependent glutamate synthase, chloroplastic~ GLU~ complete

2G7	chr9	11832587	11832766	-	2e-97	100	GSMUA_Achr9P17300_001	GSMUA_Achr9T17300_001~ Flavonoid 3',5'-hydroxylase 1~ CYP75A1~ complete
2G12	chr8	28416325	28416575	-	1E-137	99,6	GSMUA_Achr8P24130_001	GSMUA_Achr8T24130_001~ Geranylgeranyl diphosphate reductase, chloroplastic~ CHLP~ complete
2H2	chr3	7213712	7214421	-	1E-128	99,17	GSMUA_Achr3P09940_001	GSMUA_Achr3T09940_001~ Probable WRKY transcription factor 26~ WRKY26~ complete
2H5	chr10	24606255	24607294	+	1E-160	97,78	GSMUA_Achr10P17330_001	GSMUA_Achr10T17330_001~ Calnexin homolog 1~ CNX1~ complete
2H6	chr4	21859446	21862267	-	e-117	94,72	GSMUA_Achr4P21360_001	GSMUA_Achr4T21360_001~ 40S ribosomal protein S18~ RPS18A~ modules
2H7	chr9	5771119	5771604	+	9E-64	97,79	GSMUA_Achr9P08930_001	GSMUA_Achr9T08930_001~ Geranylgeranyl transferase type-2 subunit beta~ RABGGTB~ complete
2H10	chr9	33539222	33539292	+	3E-15	90,14	GSMUA_Achr9P30140_001	GSMUA_Achr9T30140_001~ Thioredoxin-like protein CXXS1~ CXXS1~ complete
2H11	chr5	15110821	15111018	+	1E-103	98,99	GSMUA_Achr5P16630_001	GSMUA_Achr5T16630_001~Transcription factor TGA1~ TGA1~ complete
2H12	chrUn_random	136799407	136799589	-	4E-99	100	GSMUA_AchrUn_randomP28100_001	GSMUA_AchrUn_randomT28100_001~ Chlorophyll a-b binding protein CP29.3, chloroplastic~ LHCB4.2~ fragment

*Identity found to a gene coding for the chloroplast 23S ribosomal RNA (blastn, nr, GenBank).

**Identity found to a gene coding for the mitocondrial 26S ribosomal RNA (blastn, nr, GenBank).

*** Identity found to a gene coding for the mitocondrial 26S ribosomal RNA (blastn, nr, GenBank).

**** Identity found to a gene coding for the chloroplast 23S ribosomal RNA (blastn, nr, GenBank).
